# Transgenerational Epigenetic Programming of the Brain Transcriptome and Anxiety Behavior

**DOI:** 10.1371/journal.pone.0003745

**Published:** 2008-11-18

**Authors:** Michael K. Skinner, Matthew D. Anway, Marina I. Savenkova, Andrea C. Gore, David Crews

**Affiliations:** 1 Center for Reproductive Biology, School of Molecular Biosciences, Washington State University, Pullman, Washington, United States of America; 2 Division of Pharmacology and Toxicology, University of Texas, Austin, Texas, United States of America; 3 Section of Integrative Biology, University of Texas, Austin, Texas, United States of America; James Cook University, Australia

## Abstract

Embryonic exposure to the endocrine disruptor vinclozolin during gonadal sex determination promotes an epigenetic reprogramming of the male germ-line that is associated with transgenerational adult onset disease states. Further analysis of this transgenerational phenotype on the brain demonstrated reproducible changes in the brain transcriptome three generations (F3) removed from the exposure. The transgenerational alterations in the male and female brain transcriptomes were distinct. In the males, the expression of 92 genes in the hippocampus and 276 genes in the amygdala were transgenerationally altered. In the females, the expression of 1,301 genes in the hippocampus and 172 genes in the amygdala were transgenerationally altered. Analysis of specific gene sets demonstrated that several brain signaling pathways were influenced including those involved in axon guidance and long-term potentiation. An investigation of behavior demonstrated that the vinclozolin F3 generation males had a decrease in anxiety-like behavior, while the females had an increase in anxiety-like behavior. These observations demonstrate that an embryonic exposure to an environmental compound appears to promote a reprogramming of brain development that correlates with transgenerational sex-specific alterations in the brain transcriptomes and behavior. Observations are discussed in regards to environmental and transgenerational influences on the etiology of brain disease.

## Introduction

Environment-genome interactions are critical to understanding how health and disease are influenced by environmental exposures. A large number of environmental factors and compounds have been shown to influence a variety of diseases [1], but few have been shown to promote DNA sequence mutations [2]. In contrast, emerging evidence suggests epigenetics (e.g. DNA methylation, RNA associated silencing and histone modifications) is involved in the ability of environmental experiences to regulate the genome and to develop stable alterations in phenotype [3]. Since epigenetics can influence genome-wide gene expression profiles (i.e. transcriptomes) of most organs and cell types [4], environmental alterations in the epigenome appears to be a mechanism potentially involved in the abnormal transcriptomes associated with disease [5].

Previous observations have demonstrated that the embryonic exposure to endocrine disrupting chemicals (i.e. vinclozolin and methoxychlor) during gonadal sex determination induces a transgenerational effect on adult male reproduction and sperm production [6]. An extension of this study demonstrated that as vinclozolin generation animals age (i.e. 6–14 months), a variety of different disease states develop in a transgenerational manner (i.e. F1–F4), including breast tumors, prostate disease, kidney disease and immune abnormalities [7], [8]. This transgenerational phenotype is transmitted through the male germ-line and paternal allele [6], [7]. Although females do develop disease [7], [9], they do not transmit this phenotype to the next generation [6], [7]. Alterations in the male germ-line epigenome were identified following endocrine disruptor (i.e. vinclozolin) exposure [6]. This epigenetic reprogramming of the male germ-line appears to allow the disease phenotype to become transgenerational [3], [6], [7], [8].

The biological process involved in the transgenerational disease phenotype involves the epigenetic programming of the germ cells during the critical period of gonadal sex determination [10], [11]. As the primordial germ cells migrate to the genital ridge and colonize the bipotential gonad, their DNA becomes de-methylated [10], [11]. The germ cell DNA is then initiated to re-methylate at the onset of sex determination in a sex-specific manner [12]. The ability to induce permanent alterations in the germ-line DNA methylation pattern is hypothesized to in part allow the phenotype to become transgenerational [3], [6], [7]. The influence this altered germ-line epigenome subsequently has on the transcriptomes of developing organs was recently shown for the testis [13] and investigated in the current study using the brain.

Brain disorders such as autism, Rett Syndrome, Fragile X Syndrome, and Angelman's Syndrome appear to manifest during postnatal neural development and involve epigenetic mechanisms. Rett syndrome is the most common form of mental retardation in young girls and is due to a mutation of MeCP2, a methylated DNA binding protein that translates DNA methylation into gene repression [14], [15], [16], [17]. Abnormal genomic imprinting (i.e. parent of origin monoallelic gene expression) can promote several inherited syndromes including Angelman's, Prader-Willi and Beckwith-Wiedemann Syndromes [18], [19]. Fragile X Syndrome results from the hypermethylation of the Fmr1 promoter and loss of FMRP expression [20]. Autism has also been associated with mutations in a methylated CpG binding protein MBD1 [21]. These previous studies suggest epigenetics has a critical role in brain development and neural disorders.

The current study was designed to investigate the actions of the endocrine disruptor vinclozolin on a potential transgenerational brain phenotype. The brain transcriptome was used to assess on a molecular level transgenerational alterations in the brain, with a focus on the hippocampus and amygdala. Behavioral assays were also performed to assess potential alterations in brain function. This allows for both a molecular analysis of brain genome effects and a behavioral analysis to demonstrate how both processes are affected. The current observations demonstrate that the exposure of a gestating female during gonadal sex determination to the environmental compound vinclozolin can promote a transgenerational effect on the brain, three generations removed from the exposure, involving alterations in the brain transcriptome and behavior.

## Results

Gestating rats were exposed to a daily intraperitoneal (IP) injection of vinclozolin during embryonic (E) day E8-E14 of development corresponding to the period of gonadal sex determination [6], [22]. Litter mate sisters were generally used for the control (vehicle) and vinclozolin treatments. The F1 generation animals from different litters were bred to generate an F2 generation and then F2 generation animals bred to generate the F3 generation. Both the control and vinclozolin generations were bred in a similar manner, with no sibling breedings, and animals were housed, fed and maintained under similar conditions. The F1 generation embryo and F2 generation germ-line are directly exposed to vinclozolin, so will have direct toxicology effects [23]. Therefore, the F3 generation was selected to investigate transgenerational effects on the brain. The adult brains were collected for histological analysis and RNA preparation. As previously reported, no gross brain morphological abnormalities were observed [7]. In addition, no alterations in serum steroid levels (i.e. progesterone, estradiol, testosterone and corticosterone) were observed (data not shown).

**Figure 1:**
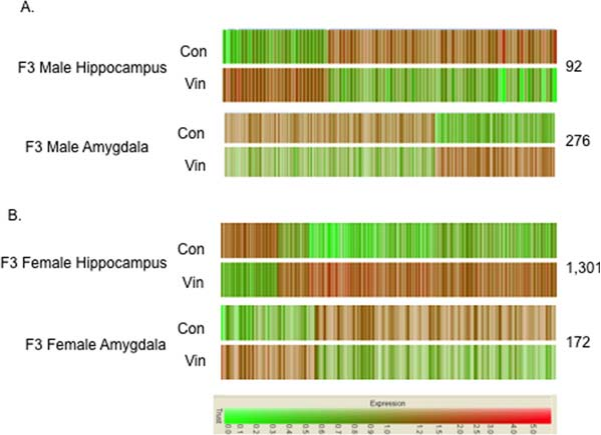
Brain transcriptome microarray analysis from F3 generation control (con) and vinclozolin (vin) animals. (A) Dendrogram for male whole brain, male hippocampus and male amygdala for statistically significant regulated genes with signal above 75. (B) Dendrogram for female hippocampus or female amygdala for statistically significant regulated genes. The scale at the bottom margin indicates an increase (red) and decrease (green) in expression. The number of regulated genes is listed at the right of each gene set. The whole male brain was from 6 month old animals, and amygdala and hippocampus from 12–15 month old animals.

The transgenerational changes in the brain transcriptomes were investigated with a microarray analysis of the adult (i.e. 12–15 months) hippocampus and amygdala. Differentially expressed genes (i.e. altered genes) were identified with a statistically significant (p<0.05) present call, a raw signal greater than 75, and a greater than 1.5-fold change in expression between control and vinclozolin generation brain samples. A dendrogram analysis is shown in Figure 1 and indicates an increase (red) or decrease (green) in gene expression between the control and vinclozolin F3 generation transcriptomes.

**Figure 2:**
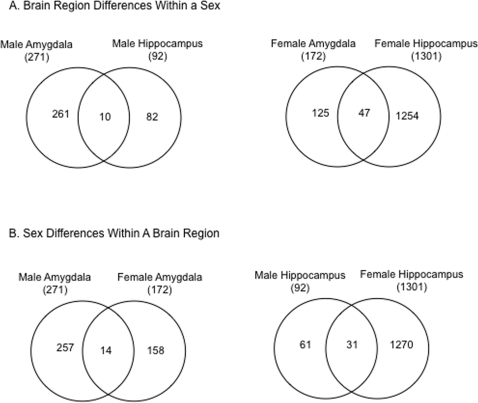
Comparison of the F3 generation regulated gene sets (i.e. transcriptomes). Venn diagram with total regulated (>1.5 fold-change between control and vinclozolin) genes listed and the overlap, (A) brain nucleus differences within a sex; (B) sex differences within a brain nucleus.

The transcriptomes of the amygdala and hippocampus in the male brain demonstrated 92 genes altered in the hippocampus and 276 genes altered in the amygdala, with the majority (70%) being decreased in expression (Figure 1). As a comparison, the F3 vinclozolin male whole brain from 6-month-old animals was analyzed and had 778 altered genes, Supplemental Table S1. Although the age of the whole brain used was different, the majority (90%) of the genes altered in the male whole brain had an increase in expression (Supplemental Table S1), suggesting numerous brain regions are likely affected. A comparison of F3 vinclozolin generation male brain transcriptomes demonstrated negligible overlap with each brain region (i.e. amygdala and hippocampus) (Figure 2). Therefore, the vinclozolin F3 generation male animals had distinct transgenerational changes in the different brain transcriptomes (Figure 2 and Supplemental Tables S2 and S3).

Analysis of the transgenerational transcriptome effects in the female hippocampus and amygdala were also assessed (Figure 1 & 2), and compared to the respective male transcriptomes. Interestingly, the differentially expressed (i.e. altered) genes in the hippocampus had the highest number of altered genes, 1,301 genes, with the majority (80%) being increased in expression, while in the amygdala 172 genes were predominately decreased (Figure 1B). The transgenerationally altered genes in the female brain are shown in Supplemental Tables S4 and S5. The altered genes in the female hippocampus and amygdala were distinct from each other and from those observed in the male (Figure 2B). A comparison of the altered genes in the female versus male amygdala or hippocampus demonstrated that the majority of the differentially expressed genes are region and sex-specific, (Figure 2 and Supplemental Tables S2, S3, S4 and S5).

**Figure 3:**
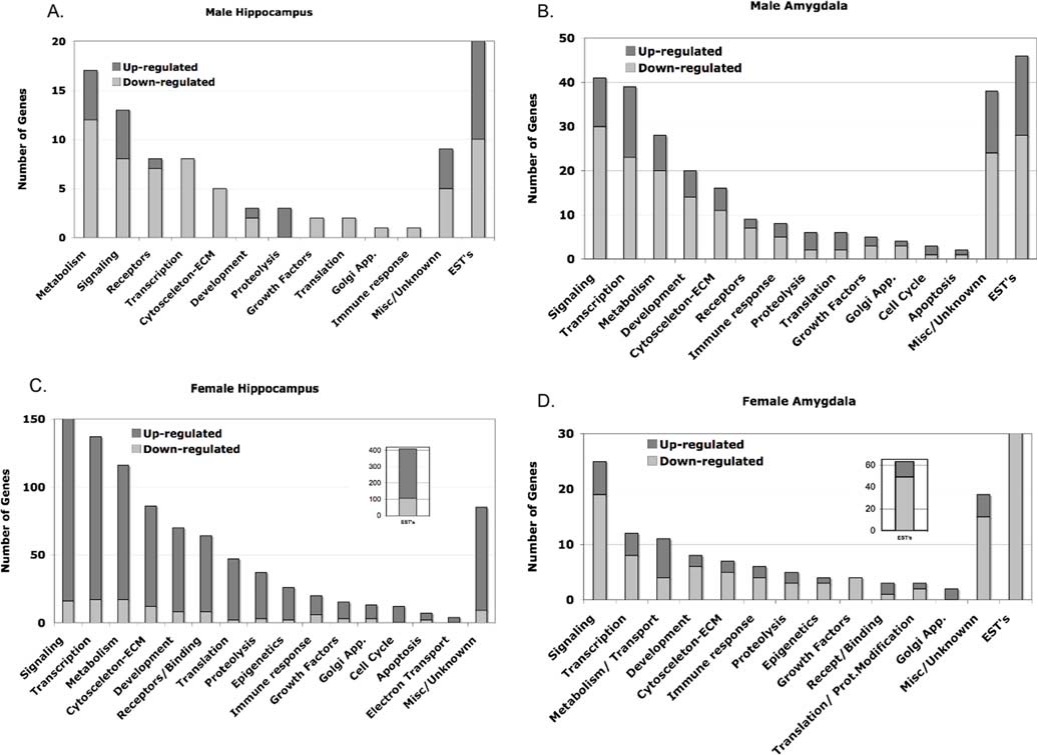
Categorization of genes into functionally related gene groups with the number of genes up-regulated or down-regulated. (A) male hippocampus regulated gene list; (B) male amygdala regulated gene list; (C) female hippocampus regulated gene list; and (D) female amygdala regulated gene list.

The transgenerationally altered genes in the F3 vinclozolin male and female generations were further analyzed. Cluster analysis of categories of genes demonstrated that genes involved in transcriptional regulation, signal transduction, and cytoskeleton were highly represented (Figure 3 and Supplemental Tables S1, S2, S3, S4 and S5). Other gene categories represented included those involved in metabolism, cell cycle, development, proteolysis and apoptosis. A mixture of up-regulated and down-regulated genes are represented in all microarray analysis (Figures 1 & 3). In addition to the full lists and categories of transgenerationally altered genes, the subsets of altered genes similar within the male and female amygdala and hippocampus are bolded in the respective Supplemental Tables S2, S3, S4 and S5. The genes similar between amygdala and hippocampus for the same sex are italicized (Tables S2, S3, S4 and S5).

Analysis of specific cellular processes and pathways within the different transgenerationally altered gene sets is shown in Table 1. The pathways containing the highest numbers of affected genes are listed. One pathway, mitogen-activated protein kinase (MAPK) signaling pathway, was affected in all of the altered gene sets. An illustration of the affected MAPK signaling pathway for the female hippocampus is presented in Supplemental Figure S1. A number of neural-pathway or neuro-processes were also affected. One commonly affected pathway was the Neuroactive Ligand-Receptor Interaction pathway. Other neural-pathways affected include axon guidance, long-term potentiation, olfactory transduction, gonadotropin releasing hormone (GnRH) signaling, melanogenesis and long-term depression. An illustration of the neuroactive ligand-receptor interaction pathway affected in the female hipppocampus is presented in Supplemental Figures S2. The female hippocampus was selected for the pathway examined in Figures S1 and S2 due to having the largest numbers of altered genes, but those genes altered in the other tissues are identified in the Figure S1 and S2 legends. Although some similarities exist, the group of affected pathways/processes within each gene set is distinct. Those pathways distinct to a specific brain region are indicated in bold in Table 1.

**Table 1:** Pathways/processes (KEGG) with the greatest number of affected genes along with any neural-pathway/processes (*) with multiple altered genes.

Altered Cellular Process/Pathway	Number Altered Genes	Score
**Male Amygdala**		
MAPK Signaling Pathway	6	1.68
Wnt/Calcium Signaling Pathways	6/4	4.0/1.17
*Neuroactive Ligand-Receptor Interactions	4	0.2
*Glioma	3	2.6
*Long-Term Potentiation	3	2.3
*Olfactory Transduction	3	5.05
**Male Hippocampus**		
Colerectal Cancer	2	2.53
**Cytokine Receptor Interactions**	2	1.65
**Jak-Stat Signaling Pathway**	2	2.03
MAPK Signaling Pathway	2	0.61
*Neuroactive Ligand-Receptor Interactions	2	0.4
**Female Amygdala**		
**Antigen Processing and Presentation**	2	3.35
**Cell Adhesion Molecules (CAMs)**	2	1.81
Colorectal Cancer	2	2.43
MAPK Signaling Pathway	2	0.64
**Nitrogen Metabolism**	2	6.46
**Female Hippocampus**		
MAPK Signaling Pathway	25	3.21
**Focal Adhesion**	19	3.02
Wnt Signaling Pathway	19	4.9
*Axon Guidance	16	3.97
*Neuroactive Ligand-Receptor Interaction	13	−1.06
*Long-Term Potentiation	11	3.87
*GnRH Signaling Pathway	10	2.29
*Melanogenesis	10	2.43
*Long-Term Depression	7	1.21

The statistical score (Score) is from a hypergeometric test with the larger the value from 0.0 (either + or −) being more significant. The bolded pathways are those specific to a brain region compared to the others.

To investigate important brain genes relevant to behavior and brain disease (e.g. schizophrenia), and to confirm the microarray analysis, a quantitative real-time PCR procedure was used to determine the expression of genes listed in Table 2, (e.g. examine catechol-o-methyltransferase (Comt) expression). The hippocampus and amygdala from control and vinclozolin F3 generation rats were collected, RNA isolated and analyzed. The ratio of gene expression between vinclozolin and control (V/C) samples is shown for both the microarray and quantitative PCR procedures. The analysis showed the same trends in expression for the majority of the genes with microarray or QPCR observations (Table 2), with slight changes in the magnitude of the effects. One gene Grik2 was found to be annotated incorrectly on the microarray chip using non-coding sequence oligonucleotides, so the QPCR procedure is accurate for this gene and not the microarray. For Vof16 female hippocampus the microarray had a greater than 10-fold increase, but for the QPCR a decrease. Further analysis demonstrated the control signal in the microarray was artificially low due to inaccurate normalization from the mismatch oligonucleotides. The Drd2 gene expression in male amygdala in the microarray had a decrease, while the QPCR had an increase, Table 2. The signal for the microarray was at the low limit of detection which may contribute to the difference. Therefore, the quantitative PCR procedure confirmed the majority of the microarray analysis, for the genes investigated that had brain disease relevance discussed below, but some differences were observed for various technical reasons.

**Table 2:** Genes related to mental health disorders that show a significant (in bold) alteration in expression in Vinclozolin-lineage rats.

Gene	Area	Cont	Vincl	V/C	Alteration	QPCR V/C
Comt (catechol-O-methyltransferase)	Male Amygdala	293	182	**0.62**	Decrease	**0.01**
	Female Amygdala	265	238	0.9	Decrease	**0.21**
	Male Hippocampus	324	149	**0.46**	Decrease	**0.60**
	Female Hippocampus	206	250	1.21	Increase	**1.60**
Grik2 (Glutamate receptor, ionotropic)	Female Amygdala	4	39	**9.84**	Increase	**5.62**
S100a4 (Calcium binding rpotein A4)	Male Amygdala	84	119	1.41	Increase	**1.28**
Bdnf (brain derived neurotropic factor)	Male Amygdala	72	113	**1.58**	Increase	**1.26**
	Female Hippocampus	137	236	**1.72**	Increase	**4.23**
Drd2 (D2 dopamine receptor)	Male Amygdala	65	37	0.56	Decrease	**2.28**
Vofl6 (Ischemia-related factor)	Male Hippocampus	43	95	2.21	Increase	**2,09**
	Female Hippocampus	7	122	**18**	Decrease	**0.88**

Listed are raw microarray signals, with V/C indicating the ratio of the signals. Although several of the V/C ratios are less than 1.5-fold changed, they are presented to allow comparison with the quantitative PCR (QPCR) results. The Real-time QPCR for the genes indicated regarding the vinclozolin/control (V/C) ratio is presented. All the microarray and QPCR ratios in **bold** were statistically significantly different (P<0.05) between control and vinclozolin values.

A correlation of several transgenerationally affected genes previously shown to be involved in brain disease or neurological disorders is shown in Table 2. We focused on genes identified by two or more studies in the literature as being likely candidate genes involved in mental disorders. The Comt gene [24], [25], [26] microarray data for male and female amygdala and hippocampus are presented. The S100 (calcium binding protein A4) [28] gene expression was increased in the male amygdala. The male amygdala and female hippocampus showed an increase in expression in brain derived neurotropic factor (BDNF) and the male amygdala had an increase in D2 dopamine receptor (DRD2) expression. There is an increase in the expression of ischemia-related factor Vof16 [28] in the male and female hippocampus. Finally, there is an increase in expression in the female amygdala of the glutamate receptor (Grik2), as determined with QPCR. Therefore, a number of genes previously shown to be involved in brain abnormalities and behavior were found to be transgenerationally altered, Table 2.

**Figure 4:**
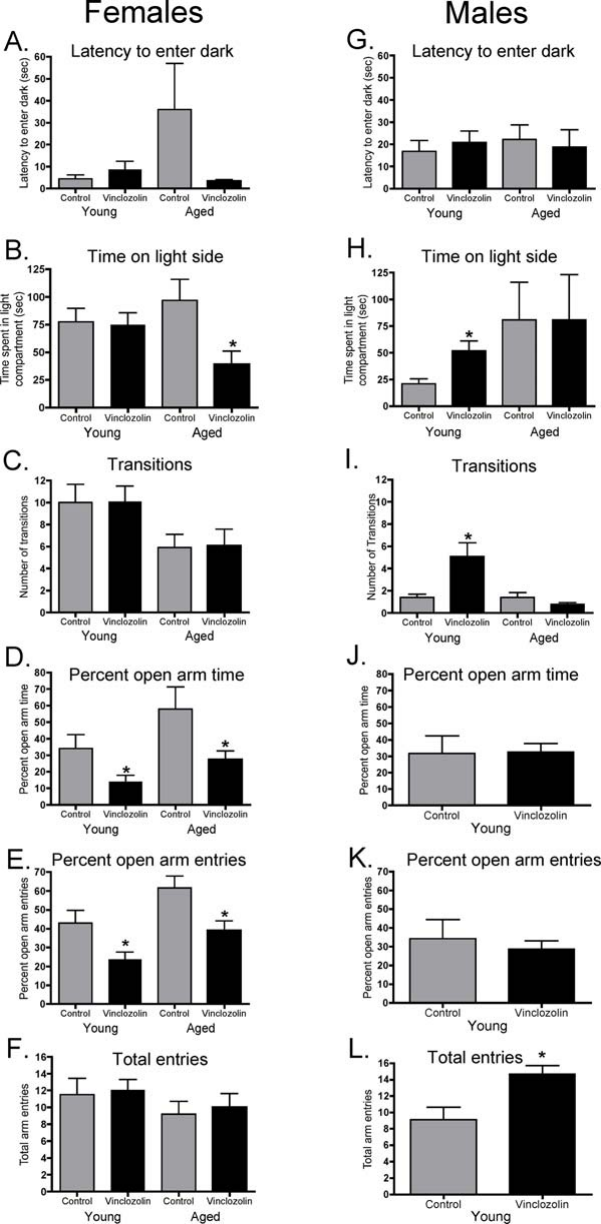
Performance in the light:dark box in young and aged third-generation female and male rats. Data are mean±SEM of latency to enter dark (A, G), time spent in the light side of the box (B, H) and the total number of transitions made between the light and dark sides of the box (C, I) in young (left panel) and aged (right panel) control and vinclozolin generation rats. For young and aged female rats N = 8–9/group. For B aged: t_15_ = 2.65, p<0.018. * P<0.05, compared with control. Performance in the elevated plus-maze in young and aged third-generation female and male rats. Data are mean±SEM of percent open arm time (D, J), percent open arm entries (E, K) and total entries (F, L) in young (left panel) and aged (right panel) control and vinclozolin generation rats. For young rats, all animals from above are shown; for aged rats, N = 5 (control) and 7 (vinclozolin). The aged group represents a subset of those run in the light:dark box shown because of technical (unstable lighting) conditions during testing for the first 5 rats; these rats were omitted from the analysis. For D young: t_15_ = 2.30, p<0.036. For E young: t_15_ = 2.51, p<0.024. For D aged: t_10_ = 2.40, p<0.037. For E aged: t_10_ = 2.82, p<0.018. *P<0.05, compared with control. For young male rats, N = 9–12/group. For aged male rats, N = 9–10/group. For H young: t_19_ = −2.65, p<0.016. For I young: t_19_ = −2.46, p<0.024. * P<0.05, compared with control. All young male animals L: t_19_ = −3.32, p<0.004. *P<0.05, compared with control.

Experiments were initiated to extend the molecular analysis to a more behavioral level. Behavioral analyses of the control and vinclozolin F3 generation male and female animals were performed (Figure 4). A light:dark box procedure and an elevated plus-maze procedure was used to measure anxiety-like behaviors and general motor activity. Both young postnatal (P) day 70–155 (P70–155) and aged postnatal (P>200) rats were used in the analysis. In brief, there was a significant effect of sex on latency to enter dark, time on the light side, and transitions for young rats and a significant effect of sex on latency to enter dark for old rats. Previously, we demonstrated that vinclozolin F3 generation animals between 6–12 months of age develop a number of different adult onset diseases [7]. Therefore, the young P70-155 animals were selected for behavioral studies to avoid any disease artifacts. However, the aged (P>200) animals were also used for comparison with the younger animal behaviors and to better correlate with the microarray analysis. Supplemental Table S6 summarizes the performance in the light:dark box under low lighting conditions for female and male young and aged rats. In general, this condition did not appear to produce the high anxiety-like states that were found under the higher lighting conditions, based on the time spent in the light compartment.

In regards to alterations in female behavior, Figure 4A–F shows the results from young and aged female rats tested in the light:dark box under high lighting conditions and in the elevated plus-maze. There were no differences between control and vinclozolin generation rats in the latency to enter the dark compartment, time spent on the light side of the box, or in the number of transitions made between the light or dark sides of the box. In aged female rats there was a tendency for vinclozolin generation rats to show a decreased latency to enter the dark side and a significant decrease in time spent in the light side of the box, with no differences in the number of transitions (Figure 4A–C). The elevated plus-maze results from young and aged females are shown in Figure 4D–F. In both age groups, vinclozolin generation rats demonstrated a decrease in both the percent time spent on the open arms and in the percent of open arm entries, with no difference in the number of total arm entries.

With respect to alterations in male behaviors, Figure 4G–L shows the response from young and aged males in the light:dark box under high lighting conditions and young males in the elevated plus maze. Young males demonstrated no difference in the latency to enter the dark side of the box, but vinclozolin generation males demonstrated a significant increase in the time spent on the light side of the box and in the number of transitions between the light and dark sides of the box. No differences between control and vinclozolin generation rats were found in the aged males. Figure 4J–L demonstrates the performance of young males on the elevated plus-maze. No differences between control and vinclozolin generation rats were present for percent open arm time or percent of open arm entries. However, there was a significant increase in the number of total arm entries in vinclozolin generation rats. Aged males were not run on the elevated plus-maze task because they were too large for the dimensions of the apparatus.

## Discussion

The current study was designed to investigate the transgenerational actions of vinclozolin on the brain transcriptome and behavior. The objective was to provide both molecular and behavioral evidence for any transgenerational effects. Previously, transgenerational effects were observed on mate preference behavior in the F3 generation vinclozolin animals [29], with females from either control or vinclozolin F3 generation having a mate preference for control generation males and not vinclozolin generation males. Due to this effect on behavior, the current study extends this analysis to investigate the transgenerational effects of vinclozolin on the brain transcriptome and anxiety-like behavior. Therefore, the previous study (29) focused on mate preference and evolutionary biology while the current study investigates molecular effects on the brain and disease associated behaviors. Although no major morphological effects were observed between the control and vinclozolin generation animals, significant transgenerational alterations were observed in the brain transcriptomes. Minimal overlap was observed between the genes altered in the hippocampus and amygdala. This finding suggests the epigenetic programming of the different brain regions are distinct, such that the eventual effect on the various transcriptomes are unique. Interestingly, the F3 generation male and female hippocampus and amygdala had transgenerational transcriptomes unique from each other. The current observations demonstrate that vinclozolin promotes a sex-specific transgenerational programming of the brain transcriptomes.

Analysis of the transgenerationally altered genes demonstrated a number of cellular processes and signal pathways being affected. Although each brain region was distinct in the individual gene set affected, a number of processes and pathways were similar (Table 1). For example, the mitogen activation pathway kinase (MAPK) signaling pathway that is responsive to a large number of regulatory growth and differentiation factors was common to all the altered transgenerational transcriptomes. Interestingly, a number of brain neuronal pathways were affected and in common with most transgenerational gene sets. These include melanogenesis, axon guidance and long-term potentiation (Table 1). A process affected in most of the altered transgenerational transcriptomes was the neuroactive ligand-receptor interaction process. Although correlation of pathways or genes with specified brain functions cannot be made, observations suggest transgenerational reprogramming of the brain transcriptome may alter behavior. Therefore, behavior studies were performed on the control and vinclozolin F3 generation animals, with a focus on anxiety-like behaviors due to the alterations in the amygdala transcriptomes. Although anxiety behaviors were the focus, the whole brain transcriptome data suggests other behavioral indices may also be influenced.

Findings revealed that vinclozolin generation female rats demonstrated an increase in anxiety-like behaviors. In the females from the vinclozolin generation lineage, both young and aged female rats showed significant decreases in percent open arm time and percent open arm entries, without an alteration in general activity level on the elevated plus-maze. In aged females, these findings were consistent with the results from the light:dark box, in which there was a decrease in time spent on the light side of the box. Young females did not demonstrate differences between control and treated rats, suggesting that the light:dark task may be less sensitive or that a different component of anxiety-like behavior is assessed in the elevated plus-maze.

In contrast to females, male rats showed decreased anxiety-like behavior in the light:dark box. In both the light:dark box and elevated plus-maze, treated young males showed hyperactivity that may be related to increased exploration, high sensation-seeking behavior, and/or increased impulsivity. An example of such behavior is an enhanced susceptibility to acquire self-administration of the abused drug, amphetamine [30]. Aged males were generally less active than younger males when tested in the light:dark box, and this difference was significant under low-lighting conditions. The decreased activity in these aged rats may be normal and partially explained by the previous observation that aged males greater than 6 months develop various diseases such as prostate disease, kidney disease, immune abnormalities and tumor development [7]. Younger animals less than six months old do not develop disease other than spermatogenic cell defects [6], [7]. Therefore, the younger animal behavioral analysis is not influenced by disease, but behavior in the older animals needs to be considered in regards to their potential disease states. The behavior analysis was performed on both younger and older animals for comparison and the older animal age better correlated with the microarray analysis.

The mechanisms mediating the sex differences in anxiety-like behavior in vinclozolin generation females and males remain unknown. Hormones and metabolites of progesterone and testosterone have been shown to alter anxiety-like behaviors [31], [32], [33], [34]. Previously we reported the F3 generation vinclozolin animals had no major effects on hormone levels [6], [7], and steroid levels for progesterone, estradiol, testosterone and corticosterone were not affected in the current study, (data not shown). Therefore, altered endocrinology does not appear to be a factor in the altered anxiety behaviors observed. The anxiety-like behavior assessed will likely involve multiple causal factors. For example, one factor that could cause alterations in the anxiety-like behavior is that, since the F2 germ line was directly exposed to vinclozolin, these F2 generation rats may rear F3 pups differently from control F2 rats, potentially leading to altered anxiety in adult F3 rats. For this reason, caution should be used in the mechanistic interpretation of the behavioral studies. For this reason we have referred to the behaviors observed as anxiety-like and caution should be used in interpretation of the behavioral studies.

The analysis of the brain transcriptomes demonstrated that a number of genes previously shown to be involved in brain disease or neurological disorders were transgenerationally altered (Table 2). For example, the catechol-o-methyltransferase (Comt) gene is expressed in the brain and associated with the etiology of schizophrenia and depression [25], [26], [27]. The serotonin transporter (SLLGA4) gene is important for brain function and linked to abnormalities such as autism [28]. The S100 calcium binding protein A4 gene has been shown to be involved in developmental stress responses [29]. The altered expression of brain derived neurotrophic factor (BDNF) and the D2 dopamine receptor (DRD2) is significant as the former has been implicated in Alzheimer's disease, affective disorders, posttraumatic stress disorder, schizophrenia, and substance dependence [35], while the latter has been implicated in post traumatic stress syndrome (PTSD), anxiety, social dysfunction, and depression [36]. Potential altered epigenetic regulation of the ischemic-related factor Vof16 gene is also associated with stress responses [29]. Therefore, a number of genes previously shown to be involved in brain abnormalities and behavior were found to be transgenerationally altered. Although causal relationships cannot be made in the current study, the role of specific transgenerationally altered genes can be investigated in the future.

Endocrine disruptors are a class of compounds that alter hormone actions and endocrinology [8], [37], [38]. Many environmental compounds from fungicides to plastics have endocrine disruptor activity [38], [39]. The endocrine disruptor used in the current study was vinclozolin, which is a fungicide used in the fruit industry [38]. Vinclozolin and its metabolites are anti-androgenic compounds that bind and alter androgen receptor actions [40]. Embryonic and early postnatal exposure to vinclozolin [22], [38], [40] or other endocrine disruptor compounds promote adult onset disease in numerous species [1], [6], [37], [39], [41]. The frequency of transgenerational disease induction and reproducibility of the phenomena [6], [7] suggests an epigenetic mechanism is involved.

In the context of the current study, two potential epigenetic sites of action for environmental toxicants need to be considered. The first is during the active development of a specific organ when the epigenome and transcriptome is undergoing a cascade of developmental stages to eventually establish the adult organ transcriptome and physiology. In the event a factor reprogrammed or altered an epigenetically labile site or metastable allele of the epigenome during this development, the adult organ may not have the proper transcriptome and become susceptible to develop disease. This epigenetically induced adult onset disease state would not be passed to subsequent generations, but may be a significant factor in disease etiology [3], [42]. In regards to behavior this has been termed a context-dependent epigenetic modification [43]. The second major epigenetic site of action involves reprogramming the epigenome of the germ-line [3], [6], [8]. The embryonic programming of the genome during gonadal differentiation could be modified to promote an abnormal epigenome. In the event this modified epigenetic program (i.e. DNA methylation) became imprinted-like, then all subsequent generation programming would be influenced [3]. The primordial germ cells undergo a de-methylation prior to gonadal development and then re-methylation in a sex-specific manner during sex determination [10], [11], [12]. The germ cells at this time of development appear to be sensitive to epigenetic re-programming [6], [7], [8]. Observations demonstrate that the endocrine disruptor vinclozolin can alter the epigenome of the male germ-line transgenerationally. The ability of this altered epigenome to promote a transgenerational epigenetic alteration in the transcriptome has been shown in the developing testis [13] and is speculated to be the mechanism behind the subsequent development of heritable adult onset disease [3], [6]. The role of epigenetic alterations in the brain cell populations as a causal factor in the transcriptome and behavior alterations remains to be elucidated in future studies.

The combined current observations indicate that vinclozolin exposure of a gestating female rat during gonadal sex determination can promote a transgenerational alteration in the brain transcriptome and behavior. We suggest this is due to an epigenetic alteration in the male germ-line that transgenerationally transmits, through an altered germ-line epigenome, an altered epigenetic reprogramming of the brain. The transgenerational changes in different regions of the brain (i.e. hippocampus and amygdala) are distinct and sex specific. These molecular changes in the brain transcriptome are associated with altered behavior, but the causal relationships remain to be established. Interestingly, the alterations in male and female behavior are different, with females developing an increase in anxiety-like behavior and males a decrease in anxiety-like behavior and an increase in activity. Therefore, an environmental compound (i.e. endocrine disruptor) exposure during pregnancy promoted a transgenerational alteration in brain programming and behavior. The observations suggest some element of neurodegenerative disease and brain abnormalities may be induced through an epigenetic transgenerational mechanism. The component of brain abnormalities that are transgenerational remains to be determined, but some neurodegenerative conditions and abnormalities are familial and have a paternal heritability. Future studies will need to determine the role of transgenerational epigenetic mechanisms in brain development, physiology and disease.

## Materials and Methods

### A. Animals & In Vivo Procedures

Gestating outbred Sprague-Dawley mother rats from timed pregnant colonies housed at the Washington State University Vivarium were given intraperitoneal (IP) injections of vinclozolin (100mg/kg/day) from embryonic day 8–14 (E8–E14) of gestation (i.e. F0 generation) as previously described [44]. Sperm positive vaginal smear date was identified as embryonic day 0. Gestating control mothers (i.e. F0 generation) received vehicle alone (i.e. sesame oil and DMSO). At least 8 lines (individual F0 injected females) were generated for controls and 8 lines for vinclozolin generations for these analyses. The F1–F3 generation animals derived from a vinclozolin exposed F0 mother are referred to as vinclozolin generation animals, while those from control F0 generation mothers are identified as control generation animals. The brains from males were collected at 12 months of age and females at 15 months of age for analysis of the hippocampus and amygdala. Adult F1 vinclozolin generation (offspring from F0 mothers) males were bred to F1 vinclozolin generation females to generate the F2 vinclozolin generation. Adult F2 vinclozolin generation males were bred to F2 vinclozolin generation females to generate the F3 vinclozolin generation. Rats for the control groups (i.e. generations F1–F3) were bred in the same manner for all the generations. No inbreeding or sibling crosses were generated. All animals were housed in the same room with similar lighting and feeding conditions. All procedures have been approved by the Washington State University Animal Use and Care Committee. The hormone assays were performed by the Assay Core Laboratory of the Center for Reproductive Biology at Washington State University. These assays were radioimmunoassays for corticosterone, progesterone, testosterone and estradiol. Serum was collected from the same animals used in the microarray analyses for hormone assays. No differences were observed between control and vinclozolin F3 generation animals in steroid levels, (data not shown).

### B. Brain Collection and Histology

The brain was removed in less than 1 minute and placed in crushed ice to chill. The brain was then cut in half along the midline. The hippocampus and amygdaloid nuclei were dissected from on side of each brain using a chilled brain mold and specific brain areas were dissected using coordinates from Paxinos and Watson (Hippocampus: Bregma −2.12 to −4.52; Amygdala Bregma: −2.30 to −3.60) within 3–5 min [45]. Tissue was placed in chilled TRIZOL (150 ml) in 1.5 ml eppendorfs tubes according to the manufacturers specifications. After all brains were dissected, the eppendorfs tubes were vortexed (15 sec) and then frozen on dry ice. Tissues collected for histology were fixed in Bouin's (Sigma St. Louis, MO) for 1 hour, embedded in paraffin and sectioned. Sections were stained with hematoxylin and eosin according to standard procedures. The Center for Reproductive Biology, Histology Core Laboratory assisted with these procedures. The animal numbers were n = 6 for vinclozolin and n = 6 for controls from 3 different lines (i.e. F0 injected mothers) for the F3 generation.

### C. Microarray Analysis and Bioinformatics

The Genomics Core in the Center for Reproductive Biology at Washington State University performed the microarray analysis as previously described [46], [47]. Briefly, RNA from control and vinclozolin F3 generation adult male whole brain or male and female hippocampus or amygdala was reverse transcribed into cDNA and cDNA was transcribed into biotin labeled RNA. Biotin labeled RNA was then hybridized to the Affymetrix rat R230 2.0 gene chips (Affymetrix, Santa Clara, California). Biotinylated RNA was then visualized by labeling with phycoerythrin-coupled avidin. The microarray chip was scanned on an Affymetrix Gene Chip Scanner 3000 (Affymetrix, Santa Clara, CA). The microarray image data were converted to numerical data with GeneChip Operating Software (GCOS version 1.2.1.001; Affymetrix) using a probe set target signal of 125. An analysis was performed with GCOS to assess the relative abundance of the transcripts based on signal and detection calls (present, absent or marginal). This information was imported into Genespring software (Silicon Genetics, Redwood City, CA) and normalized using the recommended defaults. This includes setting signal values below 0.01 to a value of 0.01, total chip normalization to the 50^th^ percentile, and normalization of each chip to the median. Unless otherwise indicated, in order for a transcript to be considered present it had to be both present in the GCOS present/absent call, and have an expression level greater than 75. Briefly, the signals from 11 perfect matched oligonucleotides for a specific gene and 11 mis-matched oligonucleotides were used to make comparisons of signals to statistically determine a present call using a one-sided Wilcoxian's signed rank test. In order for a transcript to be considered changed it had to exhibit at least a 1.5-fold change between the means of the control and vinclozolin samples and have a Students 1-tail t-test p≤0.05 between samples. The raw signal cut off was 75. Therefore, the data presented are for genes that were determined to be statistically present and found to have a statistically significant difference between control and vinclozolin samples.

Two different experiments were performed involving two different sets of animals, RNA sample preparations and microarray chips. Therefore, two control and two vinclozolin generation samples were analyzed on four different chips. This allowed a 2X2 factorial comparison with all present/absent calls and changes in expression to be statistically significant for further analysis. The R^2^ for the replicate microarray chips was found to be R^2^>0.98, which indicated negligible total variability between replicates. This R^2^ value and statistical analysis indicated that the chip number used was appropriate. The number of chips required for specific experiments has been previously reviewed [48] and 2 biological replicate chips were determined to be statistically appropriate. Previous studies have demonstrated that microarray data are validated with quantative PCR data [47], [49]. Due to the presence of 11 different oligonucleotide sets for each specific gene being used on the microarray versus only a single primer set for a gene in a quantative PCR, the microarray is more effective at eliminating false positive or negative data and provides a more robust quantitation of changes in gene expression. However, validation of microarray data was performed with six selected genes using a quantitative real-time PCR procedure. Although the magnitude of the change can vary, the absence or presence of a change is generally consistent, Table 2. A brain related genes COMT, Grik 2, S100a4, Bdnf, Drd2 and Vol16 were selected to perform quantitative PCR analyses to confirm the microarray data. The primer sets used for the Comt were forward: 5′-GAGATCTTCACGGGGTTTCA-3′ reverse: 5′-AGATGTGGTGTGAGCTGCTG-3′; for Grik 2 forward:5′ACAGTTCATCAGCCAATGCTGTGC-3′ reverse 5′AACTGCACCAAATCCAAGATGGCG-3′; for S100a4 forward 5′ATACTCAGGCAACGAGGGTGACAA-3′ reverse 5′TCATGGCAATGCAGGACAGGAAGA-3′; for Bdnf forward 5′AGAAGGTTCGGCCCAACGAAGAAA-3′ reverse 5′AGAAAGAGCAGAGGAGGCTCCAAA-3′; for Drd2 forward 5′TGACAGTCCTGCCAAACCAGAGAA-3′ reverse 5′CACACCGAGAACAATGGCAAGCAT-3′; and Vol16 forward 5′TCGGAGCGTAATACCAACAGCTCA-3′ reverse 5′ACACGTGTAGACAATGCAGAGGGA-3′. The primer sets used for the constitutively expressed S2 gene were (S2, forward 5′- GCTCGTGGAGGTAAAGCTGA-3′, and reverse 5′- TGAGACGAACCAGCACAGAG-3′). Similar observations were made with this quantitative PCR procedure and the microarray analysis, Table 2.

### D. Behavioral Analysis

Animals were housed under a 12:12 hr light:dark cycle (lights on at 07:00am) with food and water available *ad libitum*. Females designated as “young” were between postnatal day (P) 93-124 and, and males designated as “young” were between P82-155. Females designated as “aged” were between P369-386 and males designated as “aged” were between P202-385, with one rat tested at P527. The total number of animals analyzed is listed in the figure legend.

The light:dark box consisted of Plexiglas that was a total of 106 cm in length, 22 cm in width and 25 cm in height. Clear Plexiglas comprised 72 cm of the length and black Plexiglas comprised 34 cm of the total length. The clear portion had a clear floor and a white Plexiglas lid, and the black portion had a black floor and a black Plexiglas lid. A light (23 W) was placed 11 cm over the center of the white lid.

The elevated plus-maze was as previously described [50] and consisted of a “plus”-shaped platform made of black opaque Plexiglas, which was 10 cm in width and 50 cm in length, creating a 10×10 cm neutral zone in the center. The plus-maze was elevated 50 cm from the floor. Two of the arms were enclosed with black Plexiglas walls 40 cm high, with no ceiling.

F3 generational male and female Sprague-Dawley rats from control and vinclozolin lineages were used in a blinded procedure for the behavioral studies. All behavioral experiments were carried out during the first 5 hr of the light cycle, and the same rats were always tested the same time of day. The light:dark box has been used to assess the level of anxiety-like behavior in rodents. It is based on the natural aversion to bright light and open spaces [51], [52], and it has been used to model anxiety. Decreased latency to enter the dark side of the box and more time spent in the dark side are believed to signify increased anxiety levels, and pharmacological studies validate these behaviors as measures of increased anxiety. For the light:dark box, rats were placed individually into the light side of the compartment at the end opposite from the dark compartment, facing the dark compartment. The latency to enter the dark compartment, time spent in the light side of the compartment and number of transitions made between the light and dark compartments were recorded for 5 min. The rat was considered to be in a compartment only when all four paws were located in that compartment. Animals were first tested in the light:dark box with only ambient lighting (no additional lighting). One day to one week later, rats were again tested in the light:dark box exactly as described above except that additional lighting overhead was present to provide a second “dose” of conditions that might reveal differences between groups on anxiety-related behavior compared with when no overhead light was present. The light (described above) was placed on the white lid above the center of the light compartment. One week after testing in the light:dark box in which the additional lighting was used, all rats were tested in the elevated plus-maze as previously described [50]. The elevated plus-maze relies on the animal's natural fear of open spaces, and the percent time spent on the open arms and percent of open arm entries are believed to be a measure of general anxiety level [53]. For this task, rats were placed individually into the center (neutral) zone of the maze, facing an open arm. Rats were allowed to explore for a 5 min period, and the number of open and closed arm entries and time spent on the open and closed arms were recorded. Animals were considered to be in the open or closed arms only when all four paws crossed out of the neutral zone. Percent open arm time was calculated by taking the time spent in the open arms and dividing it by the sum of the time spent in the open arms plus time spent in the closed arms. The percent of open arm entries was calculated by taking the number of open arm entries and dividing it by the sum of the number of open arm plus closed arm entries.

### E. Statistical Analysis

The non-behavioral data were analyzed using a SAS program. The values were expressed as the mean±SEM. Statistical analysis was performed and the difference between the means of control and vinclozolin samples or animals were determined using a Student's t-test. In vivo experiments were repeated with 3–6 individuals for each data point. A statistically significant difference was confirmed at p≤0.05. A two-way ANOVA was used to analyze the effect of sex and treatment (control or vinclozolin) on each of the five behaviors measured on young rats and each of the three behaviors measured on both sexes of old rats. Young and old rats were analyzed separately. Before doing ANOVA, the data was tested for normality and log-transformed if necessary to normalize it. When the ANOVA reported no interaction between sex and treatment, the ANOVA re-calculated to test for the effect of sex and the effect of treatment but not their interaction. Subsequent analysis was conducted separately for each sex and each age by comparing between control and vinclozolin generation rats using a two-tailed, unpaired t-test. Statistical significance was considered if p≤0.05. All statistical differences are reported in the figure legends.

## Supporting Information

Figure S1 (8.19 MB PDF)

Figure S2 (8.74 MB PDF)

Table S1 Transgenerational Male Whole Brain Regulated Genes
(0.17 MB PDF)

Table S2 Transgenerational Male Amygdala Regulated Genes
(0.13 MB PDF)

Table S3 Transgenerational Male Hippocampus Regulated Genes
(0.11 MB PDF)

Table S4 Transgenerational Female Amygdala Regulated Genes
(0.12 MB PDF)

Table S5 Transgenerational Female Hippocampus Regulated Genes
(0.26 MB PDF)

Table S6 Performance in Light: Dark Box with Low Light Condition.
(0.06 MB PDF)

## References

[1] Gluckman PD, Hanson MA (2004). Developmental origins of disease paradigm: a mechanistic and evolutionary perspective.. Pediatr Res.

[2] Li E (2002). Chromatin modification and epigenetic reprogramming in mammalian development.. Nat Rev Genet.

[3] Jirtle RL, Skinner MK (2007). Environmental epigenomics and disease susceptibility.. Nat Rev Genet.

[4] Jiang YH, Bressler J, Beaudet AL (2004). Epigenetics and human disease.. Annu Rev Genomics Hum Genet.

[5] Egger G, Liang G, Aparicio A, Jones PA (2004). Epigenetics in human disease and prospects for epigenetic therapy.. Nature.

[6] Anway MD, Cupp AS, Uzumcu M, Skinner MK (2005). Epigenetic transgenerational actions of endocrine disruptors and male fertility.. Science.

[7] Anway MD, Leathers C, Skinner MK (2006). Endocrine Disruptor Induced Epigenetic Transgenerational Disease States.. Endocrinology.

[8] Anway MD, Skinner MK (2006). Epigenetic transgenerational actions of endocrine disruptors.. Endocrinology.

[9] Nilsson E, Anway M, Stanfield J, Skinner M (2008). Transgenerational epigenetic effects of the endocrine disruptor vinclozolin on pregnancies and female adult onset disease.. Reproduction.

[10] Hajkova P, Erhardt S, Lane N, Haaf T, El-Maarri O (2002). Epigenetic reprogramming in mouse primordial germ cells.. Mech Dev.

[11] Morgan HD, Santos F, Green K, Dean W, Reik W (2005). Epigenetic reprogramming in mammals.. Hum Mol Genet.

[12] Reik W, Walter J (2001). Genomic imprinting: parental influence on the genome.. Nat Rev Genet.

[13] Anway MD, Rekow SS, Skinner MK (2008). Transgenerational epigenetic programming of the embryonic testis transcriptome.. Genomics.

[14] Amir RE, Van den Veyver IB, Wan M, Tran CQ, Francke U (1999). Rett syndrome is caused by mutations in X-linked MECP2, encoding methyl-CpG-binding protein 2.. Nat Genet.

[15] Chen WG, Chang Q, Lin Y, Meissner A, West AE (2003). Derepression of BDNF transcription involves calcium-dependent phosphorylation of MeCP2.. Science.

[16] Klose R, Bird A (2003). Molecular biology. MeCP2 repression goes nonglobal.. Science.

[17] Martinowich K, Hattori D, Wu H, Fouse S, He F (2003). DNA methylation-related chromatin remodeling in activity-dependent BDNF gene regulation.. Science.

[18] Maher ER, Reik W (2000). Beckwith-Wiedemann syndrome: imprinting in clusters revisited.. J Clin Invest.

[19] Nicholls RD, Saitoh S, Horsthemke B (1998). Imprinting in Prader-Willi and Angelman syndromes.. Trends Genet.

[20] Zhao X, Pak C, Smrt RD, Jin P (2007). Epigenetics and Neural Developmental Disorders.. Epigenetics.

[21] Li H, Yamagata T, Mori M, Yasuhara A, Momoi MY (2005). Mutation analysis of methyl-CpG binding protein family genes in autistic patients.. Brain Dev.

[22] Uzumcu M, Suzuki H, Skinner MK (2004). Effect of the anti-androgenic endocrine disruptor vinclozolin on embryonic testis cord formation and postnatal testis development and function.. Reprod Toxicol.

[23] Skinner MK (2008). What is an epigenetic transgenerational phenotype? F3 or F2.. Reprod Toxicol.

[24] O'Donovan MC, Williams NM, Owen MJ (2003). Recent advances in the genetics of schizophrenia.. Hum Mol Genet.

[25] Tsai SJ, Hong CJ, Liao DL, Lai IC, Liou YJ (2004). Association study of a functional catechol-O-methyltransferase genetic polymorphism with age of onset, cognitive function, symptomatology and prognosis in chronic schizophrenia.. Neuropsychobiology.

[26] Tsai SJ, Hong CJ, Yu YW, Chen TJ (2004). Association study of catechol-O-methyltransferase gene and dopamine D4 receptor gene polymorphisms and personality traits in healthy young chinese females.. Neuropsychobiology.

[27] Cantor RM, Kono N, Duvall JA, Alvarez-Retuerto A, Stone JL (2005). Replication of autism linkage: fine-mapping peak at 17q21.. Am J Hum Genet.

[28] Weaver IC, Champagne FA, Brown SE, Dymov S, Sharma S (2005). Reversal of maternal programming of stress responses in adult offspring through methyl supplementation: altering epigenetic marking later in life.. J Neurosci.

[29] Crews D, Gore AC, Hsu TS, Dangleben NL, Spinetta M (2007). Transgenerational epigenetic imprints on mate preference.. Proc Natl Acad Sci U S A.

[30] Piazza PV, Ferdico M, Russo D, Crescimanno G, Benigno A (1989). Circling behavior: ethological analysis and functional considerations.. Behav Brain Res.

[31] Bitran D, Shiekh M, McLeod M (1995). Anxiolytic effect of progesterone is mediated by the neurosteroid allopregnanolone at brain GABAA receptors.. J Neuroendocrinol.

[32] Gomez C, Saldivar-Gonzalez A, Delgado G, Rodriguez R (2002). Rapid anxiolytic activity of progesterone and pregnanolone in male rats.. Pharmacol Biochem Behav.

[33] Picazo O, Fernandez-Guasti A (1995). Anti-anxiety effects of progesterone and some of its reduced metabolites: an evaluation using the burying behavior test.. Brain Res.

[34] Walf AA, Ciriza I, Garcia-Segura LM, Frye CA (2008). Antisense oligodeoxynucleotides for estrogen receptor-beta and alpha attenuate estradiol's modulation of affective and sexual behavior, respectively.. Neuropsychopharmacology.

[35] Zhang H, Ozbay F, Lappalainen J, Kranzler HR, van Dyck CH (2006). Brain derived neurotrophic factor (BDNF) gene variants and Alzheimer's disease, affective disorders, posttraumatic stress disorder, schizophrenia, and substance dependence.. Am J Med Genet B Neuropsychiatr Genet.

[36] Lawford BR, Young R, Noble EP, Kann B, Ritchie T (2006). The D2 dopamine receptor (DRD2) gene is associated with co-morbid depression, anxiety and social dysfunction in untreated veterans with post-traumatic stress disorder.. Eur Psychiatry.

[37] Crews D, McLachlan JA (2006). Epigenetics, evolution, endocrine disruption, health, and disease.. Endocrinology.

[38] Fisher JS (2004). Environmental anti-androgens and male reproductive health: focus on phthalates and testicular dysgenesis syndrome.. Reproduction.

[39] Heindel JJ (2005). The fetal basis of adult disease: Role of environmental exposures–introduction.. Birth Defects Res A Clin Mol Teratol.

[40] Kelce WR, Monosson E, Gamcsik MP, Laws SC, Gray LE (1994). Environmental hormone disruptors: evidence that vinclozolin developmental toxicity is mediated by antiandrogenic metabolites.. Toxicol Appl Pharmacol.

[41] Newbold RR, Hanson RB, Jefferson WN, Bullock BC, Haseman J (1998). Increased tumors but uncompromised fertility in the female descendants of mice exposed developmentally to diethylstilbestrol.. Carcinogenesis.

[42] Ho SM, Tang WY, Belmonte de Frausto J, Prins GS (2006). Developmental exposure to estradiol and bisphenol A increases susceptibility to prostate carcinogenesis and epigenetically regulates phosphodiesterase type 4 variant 4.. Cancer Res.

[43] Crews D (2008). Epigenetics and its implications for behavioral neuroendocrinology.. Frontiers in Neuroendocrinology: In Press.

[44] Cupp AS, Uzumcu M, Suzuki H, Dirks K, Phillips B (2003). Effect of transient embryonic in vivo exposure to the endocrine disruptor methoxychlor on embryonic and postnatal testis development.. J Androl.

[45] Burwell RD (2001). Borders and cytoarchitecture of the perirhinal and postrhinal cortices in the rat.. J Comp Neurol.

[46] McLean DJ, Friel PJ, Pouchnik D, Griswold MD (2002). Oligonucleotide microarray analysis of gene expression in follicle-stimulating hormone-treated rat Sertoli cells.. Mol Endocrinol.

[47] Shima JE, McLean DJ, McCarrey JR, Griswold MD (2004). The Murine Testicular Transcriptome: Characterizing Gene Expression in the Testis During the Progression of Spermatogenesis.. Biol Reprod.

[48] Chen JJ, Delongchamp RR, Tsai CA, Hsueh HM, Sistare F (2004). Analysis of variance components in gene expression data.. Bioinformatics.

[49] Kezele PR, Ague JM, Nilsson E, Skinner MK (2005). Alterations in the ovarian transcriptome during primordial follicle assembly and development.. Biol Reprod.

[50] Cloutier S, Forquer MR, Sorg BA (2006). Low level lindane exposure alters extinction of conditioned fear in rats.. Toxicology.

[51] Crawley JN (1981). Neuropharmacologic specificity of a simple animal model for the behavioral actions of benzodiazepines.. Pharmacol Biochem Behav.

[52] Crawley JN (1985). Exploratory behavior models of anxiety in mice.. Neurosci Biobehav Rev.

[53] File SE (1993). The interplay of learning and anxiety in the elevated plus-maze.. Behav Brain Res.

