# Supplementary material for: Transgenerational Epigenetic Programming of the Brain Transcriptome and Anxiety Behavior
Source: PLoS One. 2008 Nov 18;3(11):e3745. doi: 10.1371/journal.pone.0003745 (PMC2581440; doi:10.1371/journal.pone.0003745)
Supplement: Table S1 — Transgenerational Male Whole Brain Regulated Genes (0.17 MB PDF) [file pone.0003745.s003.pdf]

Table S1
Transgenerational Male Whole Brain Regulated Genes

Apoptosis

Sample	F3-Cont	F3-Vinc	**Vin/Con**		
Gene Symt	Raw	Raw	**Ratio**	Genbank	Gene Title
Aven_predi	97	63	**0.65**	AA899459	apoptosis, caspase activation inhibitor (predicted)
Pycard	43	81	**1.88**	BI282953	apoptosis-associated speck-like protein containing a C/
Cflar	69	126	**1.84**	AI578435	CASP8 and FADD-like apoptosis regulator
Casp1	43	108	**2.52**	D85899	caspase 1
Higd1b_pre	48	116	**2.44**	AI576009	HIG1 domain family, member 1B (predicted)
Stk17b	171	276	**1.62**	AI012590	serine/threonine kinase 17b (apoptosis-inducing)

Cell Cycle

Sample	F3-Cont	F3-Vinc	**Vin/Con**		
Gene Symt	Raw	Raw	**Ratio**	Genbank	Gene Title
Arrdc3	163	339	**2.07**	AA818910	Arrestin domain containing 3
Cdc14a_pr	42	78	**1.86**	AW915083	CDC14 cell division cycle 14 homolog A (S. cerevisiae)
Cdc73	168	285	**1.70**	BI290578	Cell division cycle 73, Paf1/RNA polymerase II comple›
Ccnd1	70	191	**2.75**	BI295861	cyclin D1
Gadd45b_p	48	138	**2.88**	BI287978	growth arrest and DNA-damage-inducible 45 beta (prec
Pak4_predi	57	87	**1.52**	BE098920	P21 (CDKN1A)-activated kinase 4 (predicted)

Cytoskeleton-ECM

Sample	F3-Cont	F3-Vinc	**Vin/Con**		
Gene Symt	Raw	Raw	**Ratio**	Genbank	Gene Title
Als2cr3	152	284	**1.86**	BG378620	amyotrophic lateral sclerosis 2 (juvenile) chromosome r
Cdh1	15	121	**7.96**	NM_031334	cadherin 1
Cdh11	118	71	**0.61**	BF419584	Cadherin 11
Cdh5_predi	111	204	**1.84**	BE110616	Cadherin 5 (predicted)
Cldn5	412	690	**1.68**	BI281680	claudin 5
Col3a1	108	357	**3.30**	BI275716	collagen, type III, alpha 1
Col4a5_pre	102	196	**1.92**	AA891834	Collagen, type IV, alpha 5 (predicted)
Col5a2	134	231	**1.73**	AI179399	collagen, type V, alpha 2
Coro1c_pre	45	80	**1.78**	BE108221	coronin, actin binding protein 1C (predicted)
Clec14a	10	134	**12.99**	BI295930	C-type lectin domain family 14, member a
Dcn	95	180	**1.90**	BM390253	decorin
Dsp	43	127	**2.99**	AW144509	Desmoplakin (predicted)
Dncl2b_pre	126	251	**1.98**	AI144946	dynein, cytoplasmic, light chain 2B (predicted)
DLP2	49	80	**1.65**	AI409460	Dynein-like protein 2
Eml1	273	458	**1.68**	BI289642	Echinoderm microtubule associated protein like 1 (pred
RGD:13032	125	217	**1.73**	BF285026	endothelial cell adhesion molecule
Emp2	14	489	**35.53**	AA818807	epithelial membrane protein 2
Eva_predic	39	97	**2.51**	BI282616	epithelial V-like antigen (predicted)
Xlkd1_pred	27	254	**9.32**	AI179260	extra cellular link domain-containing 1 (predicted)
Ecm1	58	169	**2.92**	AI407838	extracellular matrix protein 1
Fgg	4	128	**29.41**	NM_012559	fibrinogen, gamma polypeptide
Fgl2	39	175	**4.50**	BG663284	fibrinogen-like 2
Fn1	147	303	**2.07**	AA893484	fibronectin 1
Flna_predic	129	334	**2.59**	BI283060	filamin, alpha (predicted)
Gsn	573	1281	**2.24**	BI285576	gelsolin
Icam1	12	419	**35.37**	NM_012967	intercellular adhesion molecule 1
Icam2	56	225	**4.00**	AI410467	intercellular adhesion molecule 2

F11r	93	288	**3.10**	NM_053796	junctional adhesion molecule 1
Krt1-18	44	152	**3.43**	BI286012	keratin complex 1, acidic, gene 18
Krt1-19	16	253	**16.09**	BI279605	keratin complex 1, acidic, gene 19
Krt2-7_prec	27	109	**4.09**	BI284344	keratin complex 2, basic, gene 7 (predicted)
Krt2-8	41	138	**3.37**	BF281337	keratin complex 2, basic, gene 8
Lamc2	32	95	**2.99**	BM385282	lamimin, gamma 2
Lamb2	126	202	**1.60**	NM_012974	laminin, beta 2
Lamb3	45	105	**2.33**	BE101834	Laminin, beta 3
Lgals3	63	454	**7.15**	NM_031832	lectin, galactose binding, soluble 3
Lgals3bp	82	151	**1.83**	AF065438	lectin, galactoside-binding, soluble, 3 binding protein
Lmcd1_pre	52	83	**1.58**	AI602501	LIM and cysteine-rich domains 1 (predicted)
LMO7	352	968	**2.75**	AI598833	LIM domain only protein 7
Lamp3_pre	8	568	**70.52**	BF283924	lysosomal-associated membrane protein 3 (predicted)
Mgp	383	1179	**3.08**	NM_012862	matrix Gla protein
Mxra8	71	110	**1.55**	AA946351	matrix-remodelling associated 8
Mfap5_prec	18	76	**4.22**	BI283094	microfibrillar associated protein 5 (predicted)
Mfap4_prec	22	479	**21.84**	AI177059	microfibrillar-associated protein 4 (predicted)
Msn	151	738	**4.88**	BF281185	moesin
Muc1	13	155	**11.87**	BI274326	mucin 1
Myh11	111	166	**1.50**	X16262	myosin heavy chain 11
Myh6	38	112	**2.91**	NM_017239	myosin heavy chain, polypeptide 6
Myo1c	41	82	**2.02**	NM_023092	myosin IC
Mrlcb	323	874	**2.71**	X05566	myosin regulatory light chain
Myo5c_pre	41	108	**2.64**	BM386808	Myosin VC (predicted)
Myh14_pre	201	346	**1.72**	AI578120	myosin, heavy polypeptide 14 (predicted)
Myh9	181	291	**1.61**	BM392410	myosin, heavy polypeptide 9
Myl7_predi	8	100	**13.31**	AA891242	myosin, light polypeptide 7, regulatory (predicted)
Myl9_predi	106	180	**1.70**	BI279044	myosin, light polypeptide 9, regulatory (predicted)
Mylk_predic	153	360	**2.35**	AI177055	Myosin, light polypeptide kinase (predicted)
Nid	99	155	**1.57**	AI235948	nidogen (entactin)
Ogn_predic	164	349	**2.13**	AA945955	osteoglycin (predicted)
Plunc	8	377	**44.83**	BF546645	palate, lung, and nasal epithelium expressed transcript
Pdlim1	50	139	**2.78**	NM_017365	PDZ and LIM domain protein 1 (Elfin) (LIM domain prot
Col4a1_pre	85	187	**2.20**	AI176393	procollagen, type IV, alpha 1 (predicted)
Col6a1_pre	59	100	**1.68**	AI598402	procollagen, type VI, alpha 1 (predicted)
Col6a3_pre	55	109	**2.00**	AI176126	procollagen, type VI, alpha 3 (predicted)
Col12a1	48	78	**1.63**	BE108345	procollagen, type XII, alpha 1
Pgsg	298	475	**1.60**	NM_020074	proteoglycan peptide core protein
Plp2	55	194	**3.54**	BM385031	proteolipid protein 2
LOC31595	25	546	**22.22**	BF290699	Similar to claudin-18A1.2
Lrp16	154	93	**0.60**	AW524615	Similar to fibronectin leucine rich transmembrane prote
RGD15631	104	582	**5.58**	AI237685	Similar to osteoclast inhibitory lectin
MGC10951	24	101	**4.18**	AA012755	similar to tropomyosin 1, embryonic fibroblast - rat
Stom_predi	33	102	**3.14**	BI295949	stomatin (predicted)
Svil_predict	94	170	**1.81**	AI045848	supervillin (predicted)
Sdc1	108	179	**1.66**	NM_013026	syndecan 1
Tln_predict	110	194	**1.77**	BI283756	talin (predicted)
Thbd	41	411	**10.12**	AA818521	thrombomodulin
Tagln	43	151	**3.53**	NM_031549	transgelin
Tmod3	127	232	**1.83**	BG378269	tropomodulin 3
Tpm1	287	437	**1.52**	M23764	tropomyosin 1, alpha
Tpm4	211	553	**2.61**	BI281955	Tropomyosin 4
Tnnt2	23	86	**3.74**	NM_012676	troponin T2

Vil2	220	395	**1.80**	AA851304	villin 2
Vill_predict	96	145	**1.51**	BE099937	villin-like (predicted
Vim	454	881	**1.94**	NM_031140	vimentin

Development

Sample<br>Gene Symb	F3-Cont<br>Raw	F3-Vinc<br>Raw	**Vin/Con**<br>**Ratio**	Genbank	Gene Title
Adfp	42	85	**2.03**	BG673602	Adipose differentiation related protein
ADRP	54	138	**2.55**	BI285616	adipose differentiation-related protein
Jub	33	87	**2.64**	BI284400	ajuba homolog (Xenopus laevis)
Amotl2	161	275	**1.71**	BF418582	Angiomotin like 2
Amotl1_pre	152	239	**1.57**	BI277433	Angiomotin-like 1 (predicted)
Angpt1	43	88	**2.03**	AI639162	angiopoietin 1
Ace	24	167	**7.00**	AA926148	angiotensin 1 converting enzyme 1
Apeg1	152	101	**0.67**	AI072733	Aortic preferentially expressed gene 1
Bk	75	35	**0.47**	U30831	Brain and kidney protein
Crygs_pred	123	45	**0.37**	AI408238	crystallin, gamma S (predicted)
Dpt_predict	36	252	**6.95**	BI285485	dermatopontin (predicted)
Evi1_predic	64	168	**2.61**	BE107033	ecotropic viral integration site 1 (predicted)
Elavl1_prec	123	188	**1.53**	BF559812	ELAV (embryonic lethal, abnormal vision, Drosophila)-li
Emcn	64	159	**2.49**	AI172271	endomucin
Emp1	32	77	**2.42**	BI275741	epithelial membrane protein 1
Fbn1	62	96	**1.54**	BM389019	fibrillin-1
Fhl1	78	119	**1.52**	BI298356	four and a half LIM domains 1
Glipr1_prec	39	105	**2.65**	BF287967	GLI pathogenesis-related 1 (glioma) (predicted)
Gmfg	78	192	**2.46**	BG666787	glia maturation factor, gamma
Gpnmb	76	382	**5.02**	NM_133298	glycoprotein (transmembrane) nmb
Gp38	147	701	**4.76**	NM_019358	glycoprotein 38
Gpc3	45	118	**2.62**	NM_012774	Glypican 3
Heca_predi	70	125	**1.79**	BM390524	Headcase homolog (Drosophila) (predicted)
IAG2	117	223	**1.90**	AI575519	implantation-associated protein
Irx1_predic	55	184	**3.35**	BF543574	Iroquois related homeobox 1 (Drosophila) (predicted) //
Irx3_predic	55	128	**2.32**	AI030203	Iroquois related homeobox 3 (Drosophila) (predicted)
Kirrel3_pre	193	106	**0.55**	BF402633	Kin of IRRE like 3 (Drosophila) (predicted)
Mvp	75	132	**1.75**	NM_022715	major vault protein
Mog	71	122	**1.72**	BF410306	myelin oligodendrocyte glycoprotein
Mcl1	182	302	**1.66**	AI172056	myeloid cell leukemia sequence 1
Myadm	137	222	**1.62**	BI296048	myeloid-associated differentiation marker
Nedd9	115	267	**2.32**	BM392374	neural precursor cell expressed, developmentally down
Nedd9_pre	27	91	**3.39**	BF555968	neural precursor cell expressed, developmentally down
Net1	125	229	**1.83**	AI180187	Neuroepithelial cell transforming gene 1
Nrp1	149	359	**2.41**	AF016296	neuropilin 1
Npc2	898	1716	**1.91**	BI287960	Niemann Pick type C2
Odz2 /// LO	168	111	**0.66**	NM_020088	odd Oz/ten-m homolog 2 (Drosophila) /// hypothetical g
Pard6b_pre	50	115	**2.32**	BF525012	par-6 (partitioning defective 6) homolog beta (C. elegar
Prx	13	96	**7.49**	NM_023976	periaxin
Postn_pred	19	260	**13.57**	BM389026	periostin, osteoblast specific factor (predicted)
Pmp22	452	839	**1.86**	AA943163	peripheral myelin protein 22
Plac8_pred	52	217	**4.17**	BG378630	placenta-specific 8 (predicted)
Plac9_pred	61	152	**2.48**	BI276313	placenta-specific 9 (predicted)
Plvap	87	523	**5.99**	NM_020086	plasmalemma vesicle associated protein
Prg-2	136	88	**0.65**	AI711557	plasticity-related protein PRG-2
Retnla	41	198	**4.80**	NM_053333	resistin like alpha

S100a10	252	445	**1.77**	NM_031114	S-100 related protein, clone 42C
Spa17	67	113	**1.68**	NM_053482	sperm autoantigenic protein 17
Sox17_pre(	79	138	**1.75**	AI031004	SRY-box containing gene 17 (predicted)
Sox18	56	100	**1.78**	AW528157	SRY-box containing gene 18
Sox4_predi	140	234	**1.68**	AA956294	SRY-box containing gene 4 (predicted)
Sftpc	18	5858	**321.89**	AI009659	surfactant associated protein C
Sftpa1	7	745	**111.13**	NM_017329	surfactant, pulmonary-associated protein A1
Sftpb	5	1190	**255.87**	X14778	surfactant, pulmonary-associated protein B
Syn3	535	854	**1.60**	AI599265	Synapsin III
Syngr2	75	114	**1.52**	BF285314	synaptogyrin 2
Vamp8	106	316	**2.98**	NM_031827	vesicle-associated membrane protein 8 (endobrevin)

Electron Transport

Sample	F3-Cont	F3-Vinc	**Vin/Con**		
Gene Symb	Raw	Raw	**Ratio**	Genbank	Gene Title
Cybrd1_pre	33	95	**2.91**	BF419070	cytochrome b reductase 1 (predicted)
Cybasc3	43	77	**1.80**	AI179227	Cytochrome b, ascorbate dependent 3
Cyb5r3	217	354	**1.63**	J03867	Cytochrome b5 reductase 3
Cyp2b2	14	635	**45.67**	AI454613	Cytochrome P450, family 2, subfamily b, polypeptide 2
Cyp2f2	23	93	**4.12**	NM_019303	cytochrome P450, family 2, subfamily f, polypeptide 2
Cyp4b1	18	526	**29.37**	M29853	cytochrome P450, subfamily 4B, polypeptide 1
Glrx2	152	247	**1.62**	BG671304	Glutaredoxin 2 (thioltransferase) (predicted)
Gpx1	787	1182	**1.50**	S41066	glutathione peroxidase 1
Gpx2	10	202	**20.33**	AA800587	glutathione peroxidase 2
Gpx3	118	569	**4.80**	NM_022525	glutathione peroxidase 3
Prdx6	42	130	**3.06**	BF398290	Peroxiredoxin 6

Growth Factors, Cyto-, Chemokines

Sample	F3-Cont	F3-Vinc	**Vin/Con**		
Gene Symb	Raw	Raw	**Ratio**	Genbank	Gene Title
Bmp4	40	106	**2.62**	NM_012827	bone morphogenetic protein 4
Ccl5	46	153	**3.36**	NM_031116	chemokine (C-C motif) ligand 5
Ccl6	2	467	**217.16**	BE095824	chemokine (C-C motif) ligand 6
Cxcl4	88	313	**3.56**	AI169104	Chemokine (C-X-C motif) ligand 4
Cxcr4	46	94	**2.05**	AA945737	Chemokine receptor (LCR1)
Cklfsf6	77	170	**2.22**	AI406687	Chemokine-like factor super family 6 (predicted)
F3	144	222	**1.54**	NM_013057	coagulation factor 3
Ctgf	177	453	**2.56**	NM_022266	connective tissue growth factor
Egfl7	73	177	**2.42**	AF223678	EGF-like domain 7
Eps8l2_pre	112	199	**1.77**	BI285918	EPS8-like 2 (predicted)
Fgf1	86	132	**1.53**	BI289840	fibroblast growth factor 1
Igf1	94	180	**1.92**	M15481	insulin-like growth factor 1
Il18	64	147	**2.29**	AJ222813	interleukin 18
Ogn_predic	55	100	**1.82**	AA945955	osteoglycin (predicted)
Pdgfa	41	83	**2.05**	AA866419	platelet derived growth factor, alpha
Scgb3a1	3	468	**180.13**	AI011836	secretoglobin, family 3A, member 1
Cxcl16	53	134	**2.54**	BI296385	similar to chemokine (C-X-C motif) ligand 16
Vegf	324	563	**1.74**	AI175732	vascular endothelial growth factor
Wfdc1	85	130	**1.53**	BI279661	wap four-disulfide core domain 1

Golgi Apparatus

Sample	F3-Cont	F3-Vinc	**Vin/Con**		
Gene Symb	Raw	Raw	**Ratio**	Genbank	Gene Title

Gla	123	77	**0.62**	BM385090	galactosidase, alpha
Gcnt2 /// LC	93	143	**1.54**	AI234819	glucosaminyl (N-acetyl) transferase 2, I-branching enzy
Hs2st1_pre	105	68	**0.65**	AW530083	heparan sulfate 2-O-sulfotransferase 1 (predicted)
Man2a1	84	184	**2.19**	AI178793	Mannosidase 2, alpha 1
Pomt2	55	91	**1.65**	AI180194	Protein-O-mannosyltransferase 2
RGD15636	100	231	**2.30**	BF410842	Similar to chondroitin sulfate GalNAcT-2 (predicted)
LOC69048!	43	118	**2.77**	AA944162	Similar to cis-Golgi matrix protein GM130
RGD15662	571	33	**0.06**	AT005664	similar to Coatomer gamma-2 subunit (Gamma-2 coat
St3gal4	51	97	**1.91**	BI295550	ST3 beta-galactoside alpha-2,3-sialyltransferase 4
B4galt1_ma	52	85	**1.63**	BF282414	UDP-Gal:betaGlcNAc beta 1,4-galactosyltransferase, p

Immune Response

Sample	F3-Cont	F3-Vinc	**Vin/Con**		
Gene Symb	Raw	Raw	**Ratio**	Genbank	Gene Title
B2m	2504	3766	**1.50**	NM_012512	beta-2 microglobulin
Cd36	31	301	**9.70**	NM_031561	Cd36 antigen
Cd36 /// LO	11	89	**8.34**	AF072411	cd36 antigen /// similar to fatty acid translocase/CD36
Cd44	49	137	**2.80**	BI302830	CD44 antigen
Cd48	129	272	**2.11**	X13016	CD48 antigen
Cd53	103	195	**1.90**	NM_012523	CD53 antigen
Cd68_predi	17	92	**5.45**	AI177761	CD68 antigen (predicted)
Cd74	158	939	**5.96**	NM_013069	CD74 antigen (invariant polpypeptide of major histocon
Cd9	410	677	**1.65**	AI227627	CD9 antigen
Cd97_predi	38	182	**4.83**	BI296525	CD97 antigen (predicted)
Cd99	103	201	**1.96**	AI235284	CD99 antigen
HA11_RAT	192	569	**2.97**	M24024	Class I histocompatibility antigen, Non-RT1.A alpha-1 c
---	18	936	**52.27**	U07886	Polymeric immunoglobulin receptor AATTAA-containing
F2r	185	363	**1.97**	NM_012950	coagulation factor II receptor
C1s	62	121	**1.95**	D88250	complement component 1, s subcomponent
C2	51	109	**2.11**	AI716125	Complement component 2
C3	175	316	**1.81**	NM_016994	complement component 3
C4a /// C4-2	85	334	**3.92**	BI285347	complement component 4a /// complement component
C7	82	355	**4.33**	BF284922	Complement component 7
Crry	175	325	**1.86**	D42115	complement receptor related protein
Fcgr3	143	360	**2.51**	NM_053843	Fc gamma receptor II beta
Fcgr3 /// LC	137	380	**2.78**	NM_053843	Fc receptor, IgG, low affinity III /// Fc gamma receptor II
IgG-2a /// L	23	300	**12.99**	L22654	gamma-2a immunoglobulin heavy chain /// similar to ga
LOC28716	401	839	**2.09**	AI237401	globin, alpha
Gbp2	41	96	**2.34**	NM_133624	guanylate binding protein 2, interferon-inducible
LOC68032!	7	441	**65.36**	BG374683	Ig active lambda2-like chain mRNA, 3' end
Igha	42	749	**17.82**	AI412189	immunoglobulin heavy chain (alpha polypeptide)
Igh-1a_prec	5	568	**107.15**	AI411947	immunoglobulin heavy chain 1a (serum IgG2a) (predict
Igj_predicte	22	285	**13.16**	AA817898	immunoglobulin joining chain (predicted)
Ifnar1_pred	57	86	**1.51**	AA799711	interferon (alpha and beta) receptor 1 (predicted)
Ifi30_predic	122	194	**1.60**	BM389261	interferon gamma inducible protein 30 (predicted)
Ifitm1_pred	68	202	**2.99**	BG380285	interferon induced transmembrane protein 1 (predicted
Ifitm2	104	233	**2.24**	NM_030833	interferon induced transmembrane protein 2 (1-8D)
Irf2_predict	50	77	**1.53**	BI302791	interferon regulatory factor 2 (predicted)
Ifit2	44	86	**1.96**	BE118697	interferon-induced protein with tetratricopeptide repeats
Ly6b	10	333	**34.47**	M30689	Ly6-B antigen gene
Ly6c	50	214	**4.24**	NM_020103	Ly6-C antigen
C1qr1	49	163	**3.31**	BI282932	Lymphocyte antigen 68
RT1-CE12	130	262	**2.02**	M24026	RT1 class I, CE12

RT1-Aw2	170	370	**2.17**	AI012221	RT1 class Ib, locus Aw2
RT1-Aw2 //.	96	329	**3.45**	BI282965	RT1 class Ib, locus Aw2 /// RT1 class I, CE12 /// RT1 cl
RT1-Aw2 //.	215	463	**2.15**	AJ249701	RT1 class Ib, locus Aw2 /// RT1 class Ia, locus A2 /// RT
RT1-S3	56	153	**2.76**	AJ243974	RT1 class Ib, locus S3
RT1-Ba	20	181	**9.04**	BG378249	RT1 class II, locus Ba
RT1-Da	93	583	**6.25**	Y00480	RT1 class II, locus Da
RT1-Db1	19	137	**7.21**	BI279526	RT1 class II, locus Db1
LOC49898\	61	98	**1.62**	BG664827	similar to Ab2-143
LOC68040	321	208	**0.65**	BG374818	Similar to Complement C1q-like protein 3 precursor (Gl
LOC50018(	6	2341	**368.59**	L22655	similar to IG KAPPA CHAIN V-V REGION K2 PRECUR
LOC29935	33	115	**3.50**	AI411693	similar to Igh-6 protein /// similar to Ig H-chain V-region
LOC49802	26	105	**4.12**	BF387360	similar to immunoglobulin superfamily, member 7
LOC68632(	43	98	**2.27**	BI294409	similar to Interferon-alpha/beta receptor beta chain pre
LOC31450\	28	87	**3.11**	AA996557	similar to single chain Fv antibody fragment scFv 7-10A
RGD15634	207	395	**1.91**	BM391860	Similar to T-cell activation leucine repeat-rich protein (p
Tiam1_pred	75	42	**0.56**	BM389265	T-cell lymphoma invasion and metastasis 1 (predicted)
Thbd	13	103	**7.77**	BG666306	thrombomodulin

Metabolism & Transport

Sample	F3-Cont	F3-Vinc	**Vin/Con**		
Gene Symb	Raw	Raw	**Ratio**	Genbank	Gene Title
Acsl5	175	282	**1.61**	NM_053607	acyl-CoA synthetase long-chain family member 5
Aytl2_predi	186	429	**2.31**	BG380281	Acyltransferase like 2 (predicted)
Arf4l_predi	30	89	**2.95**	AI030650	ADP-ribosylation factor 4-like (predicted)
Aldh2	195	327	**1.68**	NM_032416	aldehyde dehydrogenase 2
Aldh1a1	286	631	**2.21**	NM_022407	aldehyde dehydrogenase family 1, member A1
Aldh9a1	200	329	**1.65**	BI294750	Aldehyde dehydrogenase family 9, subfamily A1
Akr1cl1_pre	45	262	**5.83**	AW522526	aldo-keto reductase family 1, member C-like 1 (predicte
Accn1	78	45	**0.58**	Y14635	amiloride-sensitive cation channel 1
Aoc3	11	187	**17.11**	AI070137	amine oxidase, copper containing 3
Alas2	159	244	**1.53**	NM_013197	aminolevulinic acid synthase 2
Ace	75	259	**3.45**	AF201331	angiotensin 1 converting enzyme 1
Aqp1	30	171	**5.78**	AA891661	aquaporin 1
Aqp5	35	232	**6.67**	NM_012779	aquaporin 5
Atp6v1c2	13	185	**14.15**	BF283053	ATPase, H+ transporting, V1 subunit C, isoform 2
Ca3	46	114	**2.49**	AB030829	carbonic anhydrase 3
Ces3	17	543	**32.31**	L46791	carboxylesterase 3
Comt	113	251	**2.23**	NM_012531	catechol-O-methyltransferase
Chia	1	210	**167.76**	AI639227	chitinase, acidic
Clic2_predi	28	95	**3.33**	BI293548	chloride intracellular channel 2 (predicted)
Clic3_predi	4	169	**42.18**	AI234249	chloride intracellular channel 3 (predicted)
Colec12	114	182	**1.60**	BI284441	collectin sub-family member 12
Cngb1	77	24	**0.31**	AJ224680	cyclic nucleotide-gated channel beta subunit 1
RGD:13029	551	898	**1.63**	NM_022501	cysteine-rich protein 2
Cyp27a1	40	111	**2.75**	M73231	cytochrome P450, family 27, subfamily a, polypeptide 1
Dguok_pred	82	52	**0.64**	BF285179	deoxyguanosine kinase (predicted)
Dpysl4	308	204	**0.66**	BF413467	dihydropyrimidinase-like 4
Acbd3	88	57	**0.65**	BF419134	DMT1-associated protein
Cybb	13	75	**5.64**	AI176057	endothelial type gp91-phox gene
Ephx1	105	167	**1.58**	NM_012844	epoxide hydrolase 1
Ftl1	1000	1596	**1.60**	L01122	ferritin light chain 1
Fmo1	59	296	**5.05**	NM_012792	flavin containing monooxygenase 1
Fmo2	13	117	**8.73**	BM389350	flavin containing monooxygenase 2

Galm	91	165	**1.81**	AA997048	galactose mutarotase (aldose 1-epimerase)
Gja4	25	81	**3.25**	NM_021654	gap junction membrane channel protein alpha 4
Gja5	38	87	**2.29**	NM_019280	gap junction membrane channel protein alpha 5
Gpt2_predi	110	57	**0.52**	AA955605	Glutamic pyruvate transaminase (alanine aminotransfe
Gstm2	33	100	**3.06**	AI169331	glutathione S-transferase, mu 2
Gpd1	79	193	**2.46**	BF399697	Glycerol-3-phosphate dehydrogenase 1 (soluble)
Gpd2	137	60	**0.44**	U08027	glycerol-3-phosphate dehydrogenase 2
Gypc_predi	57	107	**1.89**	AA944212	glycophorin C (Gerbich blood group) (predicted) /// simi
Ggta1	60	118	**1.95**	AI178222	Glycoprotein galactosyltransferase alpha 1, 3
Gimap5	19	86	**4.46**	BI294743	GTPase, IMAP family member 5
Gmps_prec	66	110	**1.67**	BI283031	guanine monphosphate synthetase (predicted)
Hk1	104	58	**0.55**	_Rat_Hexokina	hexokinase 1
Hsd11b1	360	540	**1.50**	NM_017080	hydroxysteroid 11-beta dehydrogenase 1
Ltbp1	33	89	**2.71**	NM_021587	LanC (bacterial lantibiotic synthetase component C)-lik
Lta4h	283	472	**1.67**	AW918009	Leukotriene A4 hydrolase
Lpl	167	266	**1.59**	NM_012598	lipoprotein lipase
Lcp1_predi	151	333	**2.20**	BE109711	lymphocyte cytosolic protein 1 (predicted)
Lyz	521	5571	**10.70**	L12458	lysozyme
Loxl1_predi	43	101	**2.34**	AI599031	lysyl oxidase-like 1 (predicted)
Mettl7a	270	500	**1.85**	AA946026	Methyltransferase like 7A
Mgst1	252	723	**2.87**	NM_134349	microsomal glutathione S-transferase 1
Mgst2_prec	38	130	**3.45**	BI290559	microsomal glutathione S-transferase 2 (predicted)
Mgll	208	130	**0.62**	AY081195	monoglyceride lipase
Gnptg	100	56	**0.56**	AW522848	N-acetylglucosamine-1-phosphotransferase, gamma su
Asahl_pred	62	235	**3.80**	AI412627	N-acylsphingosine amidohydrolase (acid ceramidase)-l
Asah3l_pre	65	149	**2.27**	AI137826	N-acylsphingosine amidohydrolase 3-like (predicted)
Pon3	33	355	**10.65**	BM385409	paraoxonase 3
Pnpla2_pre	84	132	**1.57**	BI278288	Patatin-like phospholipase domain containing 2 (predic
Pi4k2b	44	121	**2.77**	BM383435	Phosphatidylinositol 4-kinase type-II beta
Pspla1	32	87	**2.73**	D88666	phosphatidylserine-specific phospholipase A1
Pik3ap1_pr	34	84	**2.48**	AW916092	phosphoinositide-3-kinase adaptor protein 1 (predicted
Pla2g1b	23	160	**6.97**	AI234860	phospholipase A2, group IB
Pla2g2a	7	87	**12.06**	NM_031598	phospholipase A2, group IIA (platelets, synovial fluid)
Pla2g2d	7	108	**16.68**	AI044869	phospholipase A2, group IID
Plscr1	10	78	**7.47**	NM_057194	phospholipid scramblase 1
Ppcdc_prec	81	39	**0.48**	AI411422	Phosphopantothenoylcysteine decarboxylase (predicte
Kcnk15	105	68	**0.65**	BF405604	potassium channel, subfamily K, member 15
Kcnj16	241	152	**0.63**	AI411366	potassium inwardly-rectifying channel, subfamily J, mei
Kcnma1	113	74	**0.65**	BF386160	potassium large conductance calcium-activated channe
Selenbp1	54	175	**3.26**	NM_080892	selenium binding protein 2
Shmt1_pre	60	106	**1.77**	AI010087	serine hydroxymethyl transferase 1 (soluble) (predicted
LOC50035	96	179	**1.86**	BM391516	similar to Deoxyribose-phosphate aldolase-like
LOC36806	45	182	**4.03**	AI232716	similar to thioether S-methyltransferase
LOC36187	89	55	**0.62**	AW433978	similar to Vacuolar ATP synthase subunit S1 precursor
Scnn1a	13	84	**6.50**	NM_031548	sodium channel, nonvoltage-gated, type I, alpha polype
Scn7a	48	289	**6.09**	BF285019	sodium channel, voltage-gated, type 6, alpha polypepti
Slc13a5	105	66	**0.63**	BG381311	solute carrier family 13 (sodium-dependent citrate trans
Slc25a24_	49	106	**2.18**	BM385894	Solute carrier family 25 (mitochondrial carrier, phospha
Slc29a1	56	183	**3.25**	NM_031684	solute carrier family 29, member 1
Slc34a2	3	1161	**368.63**	NM_053380	solute carrier family 34 (sodium phosphate), member 2
Slc39a4_pr	33	104	**3.19**	AI556941	solute carrier family 39 (zinc transporter), member 4 (p
Slc4a8	52	82	**1.57**	BM385244	solute carrier family 4 (anion exchanger), member 8
Slc44a2_pr	229	344	**1.50**	AI408306	Solute carrier family 44, member 2 (predicted)

Slc9a3r2	89	155	**1.75**	BI277485	solute carrier family 9 (sodium/hydrogen exchanger), is
Slco2a1	7	240	**35.00**	AI407489	Solute carrier organic anion transporter family, member
Scd2	127	62	**0.49**	BI850137	stearoyl-Coenzyme A desaturase 2
Sqrdl	113	178	**1.57**	AI013361	sulfide quinone reductase-like (yeast)
Sult1a1	159	362	**2.27**	AF394783	sulfotransferase family 1A, phenol-preferring, member
Syt4	403	236	**0.59**	L38247	synaptotagmin 4
Tpmt	101	165	**1.63**	AI638971	thiopurine methyltransferase
Tgm2	112	321	**2.87**	NM_019386	tissue-type transglutaminase
Tmc4_pred	70	149	**2.14**	AI556940	transmembrane channel-like gene family 4 (predicted)
Unc93b_pr	74	131	**1.77**	AI234533	unc-93 homolog B (C. elegans) (predicted)
Vat1_predic	77	117	**1.52**	BI284849	vesicle amine transport protein 1 homolog (T californica
Xdh	70	199	**2.86**	NM_017154	xanthine dehydrogenase

Proteolysis

Sample	F3-Cont	F3-Vinc	**Vin/Con**		
Gene Symb	Raw	Raw	**Ratio**	Genbank	Gene Title
Adam17	147	240	**1.64**	NM_020306	a disintegrin and metalloproteinase domain 17
Anpep	31	186	**6.05**	NM_031012	alanyl (membrane) aminopeptidase
Ctsc	56	162	**2.87**	AI409046	cathepsin C
Ctsh	341	662	**1.94**	NM_012939	cathepsin H
Dpp4	20	106	**5.36**	J02997	dipeptidylpeptidase 4
Laptm5	69	165	**2.38**	BF406601	lysosomal-associated protein transmembrane 5
Napsa	3	221	**67.09**	NM_031670	napsin A aspartic peptidase
Osgepl1_pr	94	61	**0.65**	BF393066	O-sialoglycoprotein endopeptidase-like 1 (predicted)
Psme1	287	460	**1.60**	NM_017264	protease (prosome, macropain) 28 subunit, alpha
Prss23	125	432	**3.45**	AI177099	protease, serine, 23
Psmb8	57	112	**1.98**	NM_080767	proteosome (prosome, macropain) subunit, beta type 8
Serping1	92	224	**2.43**	AW915763	serine (or cysteine) peptidase inhibitor, clade G, memb
Serpinb10	4	96	**26.99**	BF399855	serine (or cysteine) proteinase inhibitor, clade B (ovalbu
Serpinb6	96	223	**2.33**	AA997359	serine (or cysteine) proteinase inhibitor, clade B (ovalbu
Serpinb1a_	80	210	**2.62**	BF411331	serine (or cysteine) proteinase inhibitor, clade B, memb
Serpinh1	118	201	**1.71**	BI285495	serine (or cysteine) proteinase inhibitor, clade H, memb
Spint2	175	370	**2.11**	AI412117	serine protease inhibitor, Kunitz type 2
Siah1a	52	79	**1.51**	NM_080905	seven in absentia 1A
LOC49869(	27	87	**3.21**	AI230591	similar to ctla-2-beta protein (141 AA)
Senp5_pre	137	210	**1.53**	BE105606	SUMO/sentrin specific protease 5 (predicted) /// similar
Timp1	39	77	**1.95**	NM_053819	tissue inhibitor of metalloproteinase 1
Timp2	456	704	**1.54**	NM_021989	tissue inhibitor of metalloproteinase 2
Timp3	221	386	**1.75**	AA893169	tissue inhibitor of metalloproteinase 3 (Sorsby fundus d
Ube3a_pre	61	96	**1.56**	BG376442	ubiquitin protein ligase E3A (predicted)
Ube1dc1_p	103	190	**1.85**	AI408025	ubiquitin-activating enzyme E1-domain containing 1 (pr

Receptor & Binding Proteins

Sample	F3-Cont	F3-Vinc	**Vin/Con**		
Gene Symb	Raw	Raw	**Ratio**	Genbank	Gene Title
Admr	15	86	**5.69**	BF551274	adrenomedullin receptor
Ager	32	882	**27.90**	NM_053336	advanced glycosylation end product-specific receptor
Apbb1ip_pr	79	126	**1.60**	BM386169	amyloid beta (A4) precursor protein-binding, family B, r
Abca3	189	303	**1.61**	BE096678	ATP-binding cassette, sub-family A (ABC1), member 3
Abcc10_pre	126	81	**0.64**	AI577346	ATP-binding cassette, sub-family C (CFTR/MRP), mem
Bzrp	90	196	**2.19**	AI008680	benzodiazepin receptor
Bmpr2	189	304	**1.61**	BF398206	Bone morphogenic protein receptor, type II (serine/thre
Calcrl	108	703	**6.53**	AA945708	calcitonin receptor-like

Dlgap1	196	84	**0.43**	BF413506	Discs, large (Drosophila) homolog-associated protein 1
Dlgap1	464	275	**0.59**	BE116953	Disks large-associated protein 1 (DAP-1) (Guanylate ki
Eng	59	153	**2.60**	BF288089	Endoglin
Edg5	25	79	**3.17**	NM_017192	endothelial differentiation, sphingolipid G-protein-couple
Ednra	23	104	**4.45**	BF414702	Endothelin receptor type A
Epha8	113	73	**0.64**	C06786	Eph receptor A8
Esrrg	194	98	**0.51**	AA996810	Estrogen-related receptor gamma
Fubp3	108	71	**0.66**	BF282112	Far upstream element (FUSE) binding protein 3 (predic
Grik2	148	70	**0.48**	AW527501	Glutamate receptor, ionotropic, kainate 2
Igfbp7_prec	795	1454	**1.83**	AI233246	insulin-like growth factor binding protein 7 (predicted)
Il13ra1	60	106	**1.76**	BF282650	interleukin 13 receptor, alpha 1
Il17r_predic	75	122	**1.63**	BI279744	interleukin 17 receptor (predicted)
Kdr	97	345	**3.54**	U93307	kinase insert domain protein receptor
Ltbp4_pred	97	186	**1.91**	BG375362	latent transforming growth factor beta binding protein 4
Ltbr_predic	56	95	**1.69**	AI705656	lymphotoxin B receptor (predicted)
Mrc1_predi	30	161	**5.44**	BI294018	mannose receptor, C type 1 (predicted)
Mrvldc1_pr	52	179	**3.42**	AI176583	MARVEL (membrane-associating) domain containing 1
Nucb2	125	214	**1.71**	NM_021663	NEFA precursor
Nrp2	59	105	**1.77**	BM386520	neuropilin 2
Osbpl1a	104	60	**0.58**	BM384713	Oxysterol binding protein-like 1A
Osbpl5_pre	94	153	**1.62**	BG372522	oxysterol binding protein-like 5 (predicted)
Pvrl3_predi	48	95	**1.96**	AW525315	poliovirus receptor-related 3 (predicted)
Ptbp1	124	199	**1.60**	X60790	polypyrimidine tract binding protein 1
Rbp1	76	124	**1.62**	NM_012733	retinol binding protein 1
S100a11	101	1257	**12.49**	BG378926	S100 calcium binding protein A11 (calizzarin)
S100a6	74	1215	**16.37**	AF140232	S100 calcium binding protein A6 (calcyclin)
S100g	8	315	**39.41**	NM_012521	S100 calcium binding protein G
S100a4	85	454	**5.31**	NM_012618	S100 calcium-binding protein A4
S100a8	32	111	**3.48**	NM_053822	S100 calcium-binding protein A8 (calgranulin A)
S100a9	81	276	**3.42**	NM_053587	S100 calcium-binding protein A9 (calgranulin B)
Sec14l3	39	171	**4.41**	AA925062	SEC14-like 3 (S. cerevisiae)
Sec14l3	14	565	**39.92**	AJ132352	SEC14-like protein 3 (45 kDa secretory protein) (rsec45
LOC305633	27	79	**2.94**	AI013888	similar to Antxr2 [anthrax toxin receptor] protein
LOC287847	832	554	**0.67**	BG380409	similar to ataxin 2-binding protein 1 isoform 2
LOC498435	56	114	**2.02**	BM385804	similar to ATP-binding cassette, subfamily G, member 3
LOC690976	108	181	**1.67**	AI230762	Similar to Calponin-2 (Calponin H2, smooth muscle) (N
RGD15651	88	1111	**12.70**	AA817993	Similar to dendritic cell-derived immunoglobulin(Ig)-like
LOC296462	162	56	**0.34**	BI292232	similar to GTP binding protein 5
LOC316130	59	99	**1.69**	BI281957	similar to mannose-6-phosphate receptor binding prote
Sftpd	21	625	**30.13**	NM_012878	surfactant associated protein D
Sftpa1	18	1949	**110.76**	BI295853	surfactant, pulmonary-associated protein A1
Tax1bp3	85	179	**2.12**	BI274159	Tax1 (human T-cell leukemia virus type I) binding prote
Thra	115	71	**0.62**	M18028	thyroid hormone receptor alpha
Trip10	53	80	**1.50**	NM_053920	thyroid hormone receptor interactor 10
Tgfbr2	257	580	**2.26**	AI408571	transforming growth factor, beta receptor II
Tyrobp	72	143	**1.97**	AI102519	Tyro protein tyrosine kinase binding protein
Vipr1	64	479	**7.54**	BI301509	vasoactive intestinal peptide receptor 1
LOC679357	179	277	**1.55**	**AI103026**	similar to interferon regulatory factor 2 binding protein 2

Signaling

Sample	F3-Cont	F3-Vinc	**Vin/Con**		
Gene Symb	Raw	Raw	**Ratio**	Genbank	Gene Title
Akap13	110	217	**1.97**	AI599048	A kinase (PRKA) anchor protein 13

Akap2	44	81	**1.86**	BF398063	A kinase (PRKA) anchor protein 2
Akap5	66	143	**2.15**	NM_133515	A kinase (PRKA) anchor protein 5
Acvrl1	30	170	**5.64**	NM_022441	activin A receptor type II-like 1
Acvr1c	134	63	**0.47**	U35025	activin receptor-like kinase 7
Arc	200	313	**1.57**	NM_019361	activity regulated cytoskeletal-associated protein
Enpep	27	108	**4.04**	AF214568	aminopeptidase A
Anxa1	78	778	**9.91**	NM_012904	annexin 1
Anxa5	329	633	**1.92**	NM_013132	annexin 5
Anxa2	197	557	**2.83**	NM_019905	Annexin A2
Anxa8_pre(	34	355	**10.33**	BM389254	annexin A8 (predicted)
Alox5ap	24	85	**3.46**	NM_017260	arachidonate 5-lipoxygenase activating protein
Btg2	101	242	**2.40**	NM_017259	B-cell translocation gene 2, anti-proliferative
Bf	58	283	**4.92**	AI639117	B-factor, properdin
Bgn	103	216	**2.10**	NM_017087	biglycan
Bst2	73	329	**4.54**	BM385476	bone marrow stromal cell antigen 2
Camk2d	88	53	**0.60**	NM_012519	calcium/calmodulin-dependent protein kinase II, delta
Crem	135	202	**1.50**	AW529408	CAMP responsive element modulator
Csnk1g3	76	45	**0.59**	NM_022855	casein kinase 1 gamma 3 isoform
Cav	158	947	**6.01**	BI285449	Caveolin
Cav2	167	631	**3.77**	BE349669	caveolin 2
Cdc42ep3_	138	231	**1.67**	AA874943	CDC42 effector protein (Rho GTPase binding) 3 (predi(
Defb3	3	1034	**382.91**	AF068861	defensin beta 3
Dab2	137	239	**1.75**	AI407821	disabled homolog 2, mitogen-responsive phosphoprote
Dusp1	367	770	**2.10**	U02553	dual specificity phosphatase 1
Dusp6	322	554	**1.72**	NM_053883	dual specificity phosphatase 6
Dyrk2_pred	101	171	**1.69**	AI501693	Dual-specificity tyrosine-(Y)-phosphorylation regulated
Eltd1	92	140	**1.52**	NM_022294	EGF, latrophilin seven transmembrane domain containi
Ehd2	72	291	**4.04**	BE110691	EH-domain containing 2
Ehd4	73	155	**2.11**	BI284307	EH-domain containing 4
Epas1	1085	2151	**1.98**	AI598434	endothelial PAS domain protein 1
Tek	108	349	**3.24**	AI710931	Endothelial-specific receptor tyrosine kinase
Efna1	33	92	**2.83**	AW531877	ephrin A1
Efnb2_pred	209	350	**1.67**	BF389666	ephrin B2 (predicted)
Egfl6	23	116	**5.09**	BF418373	epidermal growth factor-like protein 6
Fos	197	506	**2.57**	BF415939	FBJ murine osteosarcoma viral oncogene homolog
Flt1	130	204	**1.56**	AI059968	FMS-like tyrosine kinase 1
Fxyd3	63	133	**2.12**	AI009597	FXYD domain-containing ion transport regulator 3
Fgd5_predi	49	91	**1.87**	AI555744	FYVE, RhoGEF and PH domain containing 5 (predicte(
Gpr116	134	424	**3.16**	BI295477	G protein-coupled receptor 116
Gprc5a	40	354	**8.81**	BI276110	G protein-coupled receptor, family C, group 5, member
Gm2a	168	259	**1.54**	AI717483	GM2 ganglioside activator protein
Gng11	102	188	**1.84**	NM_022396	guanine nucleotide binding protein gamma subunit 11
Hspb1	118	535	**4.54**	NM_031970	heat shock 27kDa protein 1
Hspa1a	67	327	**4.90**	BI278231	heat shock 70kD protein 1A
Hspa1a /// I	45	389	**8.70**	NM_031971	heat shock 70kD protein 1A /// heat shock 70kD protein
Homer1	45	213	**4.72**	AF030088	homer, neuronal immediate early gene, 1
Itga1	57	150	**2.64**	NM_030994	integrin alpha 1
Itgal	43	110	**2.58**	BI289767	Integrin alpha L
Itgb1	230	591	**2.58**	AI177366	integrin beta 1
Itgb2	67	160	**2.40**	AI177292	integrin beta 2
Itga6	297	196	**0.66**	BE110753	integrin, alpha 6
Itgb6	18	106	**5.94**	AI070686	integrin, beta 6
Igtp	69	123	**1.80**	AI407953	interferon gamma induced GTPase

Ifitm3	146	509	**3.47**	BI285494	interferon induced transmembrane protein 3
Irf7_predict	67	128	**1.91**	BF411036	interferon regulatory factor 7 (predicted)
Iqgap1_pre	138	369	**2.67**	BM387072	IQ motif containing GTPase activating protein 1 (predic
Lcn2	3	234	**73.20**	NM_130741	lipocalin 2
Lu	71	243	**3.44**	NM_031752	Lutheran blood group (Auberger b antigen included)
Met	14	99	**6.99**	BG378920	met proto-oncogene
Map2k3_pr	78	117	**1.52**	BI283843	mitogen activated protein kinase kinase 3 (predicted)
Parva	94	155	**1.65**	AA686007	parvin, alpha
Phactr2	89	148	**1.67**	AW527533	Phosphatase and actin regulator 2
Pcyt1b	102	58	**0.57**	AI175443	Phosphorylase kinase alpha 1
Plekhf2_pre	108	194	**1.79**	AI103367	pleckstrin homology domain containing, family F (with F
RGD:72785	3	83	**31.87**	AW530225	prepro-Neuropeptide W polypeptide
Pacsin1	289	179	**0.62**	NM_017294	protein kinase C and casein kinase substrate in neuron
Prkch	78	118	**1.50**	AA799981	protein kinase C-eta
Ptprc	45	105	**2.36**	BF288130	Protein tyrosine phosphatase, receptor type, C
Ptprf	228	409	**1.80**	M60103	protein tyrosine phosphatase, receptor type, F
isg12(b)	30	144	**4.83**	AA819034	putative ISG12(b) protein
Rab2l	98	218	**2.21**	BI295991	RAB2, member RAS oncogene family-like
Rab27a	54	107	**1.97**	BM389524	RAB27A, member RAS oncogene family
Rab3d	51	80	**1.55**	M83681	RAB3D, member RAS oncogene family
Rab5b_pre	138	91	**0.66**	BF560790	RAB5B, member RAS oncogene family (predicted)
Rab8b	169	270	**1.60**	BF398743	RAB8B, member RAS oncogene family
Rin2_predic	190	353	**1.85**	BM392293	Ras and Rab interactor 2 (predicted)
Rassf7_pre	58	89	**1.54**	BM385768	Ras association (RalGDS/AF-6) domain family 7 (predi
Rhoj_predic	53	108	**2.02**	BM389644	ras homolog gene family, member J (predicted)
Rasip1_pre	75	149	**2.00**	AA848597	Ras interacting protein 1 (predicted)
Rac2_predi	53	167	**3.17**	AI010476	RAS-related C3 botulinum substrate 2 (predicted)
Ramp2	210	556	**2.64**	NM_031646	receptor (calcitonin) activity modifying protein 2
Rims2	76	49	**0.64**	NM_053945	regulating synaptic membrane exocytosis 2
Arhgdib_pr	138	304	**2.21**	BF285771	Rho, GDP dissociation inhibitor (GDI) beta (predicted)
Rgnef_prec	48	90	**1.90**	AW521384	Rho-guanine nucleotide exchange factor (predicted)
Scgb1a1	18	5790	**318.15**	NM_013051	secretoglobin, family 1A, member 1
Sgk	360	654	**1.82**	NM_019232	serum/glucocorticoid regulated kinase
Shc1	53	107	**2.03**	BF548525	SHC (Src homology 2 domain-containing) transforming
RGD15627	70	117	**1.67**	BG669292	Similar to ABI gene family, member 3 (NESH) binding p
RGD15635	87	50	**0.58**	BF401477	similar to AP2 associated kinase 1 (predicted)
LOC68829	130	85	**0.65**	BG371889	similar to Retinal rod rhodopsin-sensitive cGMP 3,5-cyc
Snf1lk	41	78	**1.89**	NM_021693	SNF1-like kinase
Snx4_predi	374	242	**0.65**	AI029221	Sorting nexin 4 (predicted)
Scap2	103	203	**1.98**	NM_130413	src family associated phosphoprotein 2
Socs2	140	220	**1.57**	BM384088	Suppressor of cytokine signaling 2
Tns	84	382	**4.57**	BF290193	Tensin
Tenc1_prec	92	151	**1.65**	BI294836	tensin like C1 domain containing phosphatase (predicte
Tspan8	52	392	**7.58**	NM_133526	Tetraspanin 8
Tagln2_pre	82	454	**5.53**	BI285865	transgelin 2 (predicted)
Tacstd2	20	122	**6.02**	AA891826	tumor-associated calcium signal transducer 2
Vasp_predi	102	213	**2.10**	AW520792	vasodilator-stimulated phosphoprotein (predicted)
Wif1	52	139	**2.68**	NM_053738	Wnt inhibitory factor 1
Anxa4	82	228	**2.78**	BM385237	ZAP 36/annexin IV

Transcription

Sample	F3-Cont	F3-Vinc	**Vin/Con**		
Gene Symb	Raw	Raw	**Ratio**	Genbank	Gene Title

Aebp1_pre	93	203	**2.20**	BI278482	AE binding protein 1 (predicted)
Ahnak	274	1327	**4.85**	AI407114	AHNAK nucleoprotein (desmoyokin)
Rai14	48	124	**2.58**	AI234852	Ankycorbin (Ankyrin repeat and coiled-coil structure-co
Bcl11b_pre	421	190	**0.45**	BM390227	B-cell leukemia/lymphoma 11B (predicted)
Bcor_predic	53	87	**1.62**	BI289386	Bcl6 interacting corepressor (predicted)
Cebpb	24	104	**4.39**	NM_024125	CCAAT/enhancer binding protein (C/EBP), beta
Copeb	90	233	**2.60**	NM_031642	core promoter element binding protein
Ddx58_pre	42	75	**1.80**	AI575264	DEAD (Asp-Glu-Ala-Asp) box polypeptide 58 (predictec
Dnajc1_pre	82	140	**1.70**	BI295806	DnaJ (Hsp40) homolog, subfamily C, member 1 (predic
Elf1	45	76	**1.70**	AA818055	E74-like factor 1 (ets domain transcription factor)
Ebf	119	65	**0.55**	AI406967	Early B-cell factor 1
Egr2	92	162	**1.76**	NM_053633	early growth response 2
Egr4	74	123	**1.66**	NM_019137	early growth response 4
Elk3_predic	74	145	**1.96**	BM392135	ELK3, member of ETS oncogene family (predicted)
Fos	197	506	**2.57**	BF415939	FBJ murine osteosarcoma viral oncogene homolog
FoxA1	6	76	**12.63**	BM383972	Forkhead box A1
Fhl1	620	996	**1.61**	BG673187	four and a half LIM domains 1
Gata2	37	107	**2.91**	NM_033442	GATA binding protein 2
Gata6	7	170	**25.34**	AA800786	GATA binding protein 6
Hes1	48	126	**2.63**	NM_024360	hairy and enhancer of split 1 (Drosophila)
Hmgb2 /// L	55	132	**2.40**	NM_017187	high mobility group box 2 /// similar to High mobility gro
Hoxa5	22	77	**3.44**	BE107303	homeo box A5
Hod	317	1216	**3.84**	NM_133621	homeobox only domain
Nr4a1	192	431	**2.24**	NM_024388	immediate early gene transcription factor NGFI-B
Id1	167	386	**2.31**	M86708	Inhibitor of DNA binding 1, helix-loop-helix protein (splic
Junb	91	144	**1.57**	NM_021836	Jun-B oncogene
Klf2	102	654	**6.45**	BM385790	Kruppel-like factor 2
Klf4	50	307	**6.20**	NM_053713	Kruppel-like factor 4 (gut)
Klf5	58	133	**2.28**	NM_053394	Kruppel-like factor 5 (intestinal)
Lrrfip1	21	102	**5.00**	BM387864	Leucine rich repeat (in FLII) interacting protein 1
Mfhas1_pre	109	170	**1.55**	AI136864	malignant fibrous histiocytoma amplified sequence 1 (p
Mgea6_pre	143	237	**1.65**	AI177180	meningioma expressed antigen 6 (coiled-coil proline-ric
Mcl1	308	606	**1.97**	AI102618	Myeloid cell leukemia sequence 1
Nfe2l2	182	295	**1.62**	NM_031789	NF-E2-related factor 2
Nfix	1251	2557	**2.04**	AI555855	nuclear factor I/X
Nfkbia	114	326	**2.87**	AW672589	nuclear factor of kappa light chain gene enhancer in B-
Nr4a3	167	329	**1.98**	NM_031628	nuclear receptor subfamily 4, group A, member 3
Ptrf_predict	566	1369	**2.42**	AA899937	Polymerase I and transcript release factor (predicted)
Runx1	39	92	**2.34**	NM_017325	runt related transcription factor 1
Stat1	118	182	**1.54**	AW434718	signal transducer and activator of transcription 1
LOC68753	20	81	**3.99**	AA817785	similar to Forkhead box protein F1 (Forkhead-related p
MGC72578	84	137	**1.62**	BF420059	similar to immediate early response 2
LOC49949	992	1768	**1.78**	BE104219	similar to MADS box transcription enhancer factor 2, pc
RGD15631	739	285	**0.39**	BF414312	similar to MADS box transcription enhancer factor 2, pc
RGD13063	44	100	**2.26**	AI407047	similar to Zinc finger protein 198 (Fused in myeloprolife
Sp110_pre	68	117	**1.73**	AI411381	SP110 nuclear body protein (predicted)
Sox13_pre	55	102	**1.86**	BM383170	SRY (sex determining region Y)-box 13 (predicted)
Ssg1	69	125	**1.81**	AI235465	steroid sensitive gene 1
Tnks_predi	99	169	**1.70**	BM388725	tankyrase, TRF1-interacting ankyrin-related ADP-ribose
Tiparp_pre	109	165	**1.51**	AI179464	TCDD-inducible poly(ADP-ribose) polymerase (predicte
Titf1	17	217	**12.77**	BF389361	thyroid transcription factor 1
Tcf21	12	97	**8.25**	BE113336	transcription factor 21
Tacstd1	20	217	**10.97**	BG376410	tumor-associated calcium signal transducer 1

Txnip	237	789	**3.33**	U30789	upregulated by 1,25-dihydroxyvitamin D-3
Ets1	69	175	**2.53**	NM_012555	v-ets erythroblastosis virus E26 oncogene homolog 1 (a
Erg	31	85	**2.76**	NM_133397	v-ets erythroblastosis virus E26 oncogene like (avian)
Jun	253	544	**2.15**	BI288619	v-jun sarcoma virus 17 oncogene homolog (avian)
Zfp36	67	242	**3.62**	AB025017	zinc finger protein 36
Zfp36l1	316	488	**1.54**	NM_017172	zinc finger protein 36, C3H type-like 1
Zfp628_pre	118	60	**0.51**	BE097029	Zinc finger protein 628 (predicted)
RGD:62867	109	68	**0.62**	AF277900	zinc finger protein HIT-4
Zcchc8_pre	123	82	**0.66**	AA894084	zinc finger, CCHC domain containing 8 (predicted)
Zfyve28_pr	95	60	**0.63**	BF403058	Zinc finger, FYVE domain containing 28 (predicted)

Translation_Protein Modification

Sample	F3-Cont	F3-Vinc	**Vin/Con**		
Gene Symb	Raw	Raw	**Ratio**	Genbank	Gene Title
Ear11	2	100	**40.80**	D88586	eosinophil-associated, ribonuclease A family, member 1
Dcps	44	76	**1.74**	AI535521	mRNA decapping enzyme
Dcps	121	64	**0.53**	BI288131	mRNA decapping enzyme
Paip1_pred	338	520	**1.54**	AA963084	Polyadenylate binding protein-interacting protein 1 (pre
Slfn2_predi	42	122	**2.94**	AW915529	schlafen 2 (predicted)
Slfn3	31	125	**4.06**	NM_053687	schlafen 3
Slfn5_predi	63	172	**2.75**	AI072951	schlafen 5 (predicted)
Cugbp2	67	117	**1.74**	BF390754	CUG triplet repeat, RNA binding protein 2

Miscellaneous & Unknown

Sample	F3-Cont	F3-Vinc	**Vin/Con**		
Gene Symb	Raw	Raw	**Ratio**	Genbank	Gene Title
Lrrc4c_pre	209	112	**0.53**	AA819053	leucine rich repeat containing 4C (predicted)
Angel1_pre	79	38	**0.49**	AI578461	angel homolog 1 (Drosophila) (predicted)
---	47	106	**2.24**	BM386869	CDNA clone IMAGE:7366335
Cttnbp2nl_	141	232	**1.64**	BM383195	CTTNBP2 N-terminal like (predicted)
Fam38a_pr	23	75	**3.20**	BI274438	family with sequence similarity 38, member A (predicted
Gm1960	11	276	**24.89**	AW917501	gene model 1960, (NCBI)
Gm1960	4	958	**228.15**	D21095	gene model 1960, (NCBI)
RGD13115	100	163	**1.62**	BF288207	hypothetical LOC315055 (predicted)
LOC49854	367	1672	**4.55**	AA800750	hypothetical protein LOC498544
Kirrel3_pre	347	211	**0.61**	BF399121	kin of IRRE like 3 (Drosophila) (predicted)
Lims2_prec	30	75	**2.51**	BI275904	LIM and senescent cell antigen like domains 2 (predicte
Mtac2d1	35	194	**5.57**	BI302544	membrane targeting (tandem) C2 domain containing 1
Perc64	35	103	**2.93**	BM384701	PE responsive protein c64
Perc64	63	176	**2.78**	BI278571	PE responsive protein c64
Pqlc3	66	115	**1.75**	AW915795	PQ loop repeat containing 3
Sema3g	210	387	**1.84**	BE108859	Sema domain, immunoglobulin domain (Ig), short basic
Sema3c_pr	103	238	**2.30**	BM390322	sema domain, immunoglobulin domain (Ig), short basic
Sdpr	82	802	**9.79**	AI009714	serum deprivation response protein
Sh2d4a_pr	21	97	**4.69**	AA800626	SH2 domain containing 4A (predicted)
Stac2_pred	81	48	**0.60**	BF406342	SH3 and cysteine rich domain 2 (predicted)
Spn	34	126	**3.74**	BF550890	sialophorin
---	51	84	**1.65**	AA858591	similar HORMA domain containing 1 (predicted)
MGC10949	44	93	**2.10**	AI071166	similar to 1110007F12Rik protein
---	69	232	**3.35**	BM385779	Similar to 1300014I06Rik protein (predicted)
RGD13095	47	77	**1.63**	AI102745	Similar to 2310014H01Rik protein (predicted)
RGD13069	34	81	**2.38**	AA819788	similar to 5830458K16Rik protein (predicted)
RGD13048	123	81	**0.66**	AI044301	similar to 6430573F11Rik protein (predicted)

---	29	162	**5.67**	BE109323	Similar to 9530046H09Rik protein (predicted)
LOC309112	59	115	**1.96**	AI178948	similar to BC026645 protein
LOC680229	334	145	**0.43**	AI413058	Similar to Beta-sarcoglycan (Beta-SG) (43 kDa dystrop
RGD15594	98	148	**1.51**	BF291168	Similar to BH3-only member B protein (predicted)
LOC679937	25	316	**12.85**	BM386323	similar to CG4025-PA
RGD13081	53	85	**1.59**	AA899109	similar to chromosome 17 open reading frame 27 (pred
RGD15651	16	179	**10.93**	AI045955	similar to Clecsf12 protein (predicted)
LOC304138	150	475	**3.17**	AA818900	similar to cysteine and tyrosine-rich protein 1
RGD15663	37	95	**2.59**	AA946147	Similar to cysteine-rich glycoprotein (predicted)
LOC691657	37	315	**8.43**	BF550246	Similar to Cysteine-rich protein 1 (Cysteine-rich intestin
RGD13080	118	190	**1.61**	AI408727	similar to DNA segment, Chr 4, Brigham & Womens Ge
LOC499322	13	226	**17.06**	AA799328	similar to expressed sequence AW413625
LOC687266	29	103	**3.50**	AI072658	similar to Folylpolyglutamate synthase, mitochondrial p
LOC291964	51	84	**1.64**	BI281796	similar to Formin homology 2 domain containing 1
RGD13075	41	91	**2.23**	AI231799	similar to Friedreich ataxia region gene X123 (predicted
RGD13105	67	116	**1.74**	BG375354	similar to hypothetical protein FLJ14146 (predicted)
RGD13113	90	158	**1.77**	BI294811	similar to hypothetical protein FLJ20037 (predicted)
RGD15596	399	247	**0.62**	BE101933	similar to hypothetical protein FLJ25477 isoform 2 (pre
LOC363091	5	128	**24.46**	AI103939	similar to hypothetical protein FLJ30973
RGD13058	36	102	**2.85**	BI288939	similar to hypothetical protein FLJ40283 (predicted)
LOC500300	41	109	**2.68**	BM390487	similar to hypothetical protein MGC6835
MGC12488	138	253	**1.83**	AI176611	Similar to Mob4B protein
RGD15619	48	87	**1.82**	BM390809	similar to Muc5b protein (predicted)
LOC309499	34	88	**2.57**	AW917760	similar to Myoferlin (Fer-1 like protein 3)
RGD15616	174	327	**1.88**	AA997745	similar to nemo like kinase (predicted)
LOC686753	28	250	**8.87**	AA851385	similar to nephronectin isoform a
LOC287346	56	177	**3.16**	BF284080	similar to novel protein
LOC287346	47	144	**3.09**	AA957487	similar to novel protein
LOC315732	31	75	**2.46**	BI276339	similar to Nuclear membrane binding protein NUCLING
B130017i01	145	319	**2.20**	AI407483	Similar to Parg1-pending protein (predicted)
LOC684871	19	129	**6.88**	AI230625	similar to Protein C8orf4 (Thyroid cancer protein 1) (TC
MGC10877	91	459	**5.03**	BM391248	similar to RIKEN cDNA 1810057C19
LOC498642	42	95	**2.28**	AW252379	similar to RNA binding protein gene with multiple splicir
LOC690777	38	78	**2.04**	BF404935	Similar to RUN and FYVE domain-containing 2
LOC362057	70	143	**2.03**	BF285490	similar to semaF cytoplasmic domain associated protei
RGD15625	72	110	**1.53**	AW142608	similar to SH3-containing guanine nucleotide exchange
LOC686545	84	53	**0.63**	AW527248	similar to slit homolog 1
MGC94010	187	338	**1.80**	BM384419	Similar to SPI6
RGD15621	269	162	**0.60**	AW521222	Similar to TAFA5
LOC500040	127	199	**1.57**	BI288769	similar to Testis derived transcript
LOC315843	46	114	**2.49**	BE107185	similar to WD repeat domain 11 protein
Slitrk1_pred	157	94	**0.60**	AW523107	SLIT and NTRK-like family, member 1 (predicted)
Spsb1_pred	80	49	**0.61**	BE106389	SplA/ryanodine receptor domain and SOCS box contai
Samd8	85	50	**0.59**	BI295733	sterile alpha motif domain containing 8
Tmem10_p	487	324	**0.67**	AI717253	transmembrane protein 10 (predicted)
Tmem123	94	169	**1.81**	AI171651	transmembrane protein 123//transmembrane protein 12
Tnrc6_pred	578	872	**1.51**	BI294732	Trinucleotide repeat containing 6 (predicted)
Trim23	135	87	**0.64**	L04760	tripartite motif protein 23
Trim_predic	61	105	**1.73**	BI280655	Tripartite motif protein 34 (predicted)
RGD:73507	76	163	**2.16**	AI009530	Unknown (protein for MGC:72614)
Wfdc2	26	265	**10.10**	AI411527	WAP four-disulfide core domain 2

EST

Sample Gene Symb	F3-Cont Raw	F3-Vinc Raw	**Vin/Con Ratio**	Genbank	Gene Title
---	138	222	**1.61**	AI031032	Transcribed locus, weakly similar to XP_341951.1 PRE
---	123	222	**1.80**	AI598401	Transcribed locus, weakly similar to NP_002639.1 pim-
---	49	76	**1.55**	BM385157	Transcribed locus, strongly similar to XP_001058445.1
---	270	429	**1.59**	BI289625	Transcribed locus, strongly similar to NP_035333.2 pro
---	131	79	**0.60**	BF403401	Transcribed locus, moderately similar to XP_580018.1
---	91	215	**2.36**	BI278962	Transcribed locus, moderately similar to NP_795929.1
---	26	128	**4.89**	BM389422	Transcribed locus, moderately similar to NP_694996.2
---	127	79	**0.62**	BF403165	Transcribed locus
---	68	117	**1.73**	AI175779	Transcribed locus
---	65	106	**1.64**	AI408264	Transcribed locus
---	150	92	**0.62**	BF549454	Transcribed locus
---	95	57	**0.61**	BF391706	Transcribed locus
---	27	103	**3.86**	AI233857	Transcribed locus
---	106	201	**1.90**	AI411809	Transcribed locus
---	179	478	**2.68**	BF283404	Transcribed locus
---	63	101	**1.60**	BE116601	Transcribed locus
---	45	118	**2.62**	BM384377	Transcribed locus
---	52	175	**3.36**	BF407452	Transcribed locus
---	112	237	**2.11**	BI283685	Transcribed locus
---	67	107	**1.60**	AI169118	Transcribed locus
---	134	215	**1.60**	AI709424	Transcribed locus
---	104	161	**1.54**	AA900322	Transcribed locus
---	761	501	**0.66**	AA925373	Transcribed locus
---	40	129	**3.25**	AI058292	Transcribed locus
---	36	300	**8.39**	BE107090	Transcribed locus
---	106	166	**1.56**	BE103875	Transcribed locus
---	84	54	**0.64**	AA924955	Transcribed locus
---	182	279	**1.53**	BF545309	Transcribed locus
---	29	78	**2.72**	AA818143	Transcribed locus
---	220	398	**1.81**	BF563716	Transcribed locus
---	67	147	**2.20**	AI178627	Transcribed locus
---	45	106	**2.37**	AI410262	Transcribed locus
---	13	206	**15.76**	AA859079	Transcribed locus
---	107	65	**0.61**	BE099664	Transcribed locus
---	162	100	**0.62**	BE117653	Transcribed locus
---	340	20	**0.06**	AA964506	Transcribed locus
---	77	116	**1.51**	AW918368	Transcribed locus
---	311	587	**1.89**	AI144865	Transcribed locus
---	288	504	**1.75**	AI071538	Transcribed locus
---	73	143	**1.96**	AI228481	Transcribed locus
---	162	99	**0.61**	AW531387	Transcribed locus
---	747	466	**0.62**	AI100827	Transcribed locus
---	46	120	**2.64**	BE115859	Transcribed locus
---	120	181	**1.51**	AI502604	Transcribed locus
---	27	81	**3.03**	BF389682	Transcribed locus
---	48	110	**2.30**	BI277619	Transcribed locus
---	49	77	**1.55**	AA818282	Transcribed locus
---	427	278	**0.65**	AA955175	Transcribed locus
---	57	105	**1.84**	AI408734	Transcribed locus
---	171	281	**1.64**	BI279751	Transcribed locus
---	485	914	**1.89**	BE107419	Transcribed locus

---	43	116	**2.67**	BE101285	Transcribed locus
---	124	221	**1.79**	BF551377	Transcribed locus
RGD13052	39	77	**1.95**	BF393865	similar to RIKEN cDNA C130022K22 gene (predicted)
LOC36289	105	170	**1.61**	AA859508	similar to RIKEN cDNA 9330161F08
RGD13111	297	562	**1.89**	AI716248	similar to RIKEN cDNA 9230117N10 (predicted)
RGD13070	150	276	**1.85**	BM384306	similar to RIKEN cDNA 9130404D14 (predicted)
LOC31351	41	121	**2.95**	AW915484	similar to RIKEN cDNA 5330440M15 /// similar to hypot
RGD13115	80	49	**0.61**	BF407634	similar to RIKEN cDNA 5033405K12 (predicted)
LOC49980	102	171	**1.68**	BE328914	similar to RIKEN cDNA 4933404M02
RGD13064	117	67	**0.58**	BF396478	similar to RIKEN cDNA 4930556P03 (predicted)
RGD13050	143	408	**2.84**	AI171466	similar to RIKEN cDNA 2700055K07 (predicted)
Rod1	212	389	**1.84**	BI280285	Similar to RIKEN cDNA 2610207I16 (predicted)
RGD15630	95	150	**1.57**	BF291140	similar to RIKEN cDNA 2010106G01 (predicted)
LOC36306	5	113	**22.77**	AI599133	similar to RIKEN cDNA 1600029D21
---	46	89	**1.94**	AA924459	Similar to RIKEN cDNA 1200013B08
RGD13103	107	182	**1.70**	H33457	similar to RIKEN cDNA 1200004M23 (predicted)
LOC50084	84	219	**2.61**	AI178214	LOC500840
LOC50071	29	92	**3.16**	AW919728	LOC500719 /// LOC500720 /// LOC500721
LOC50048	53	250	**4.76**	BM387041	LOC500488
LOC49931	175	353	**2.02**	BM388445	LOC499318
LOC49826	30	102	**3.44**	AI177743	LOC498261
LOC49795	49	97	**1.98**	BF399335	LOC497957
---	112	69	**0.62**	BI273954	---
---	614	326	**0.53**	BF393011	---
---	90	495	**5.50**	BF542239	---
---	81	49	**0.60**	AW527725	---
---	111	182	**1.64**	BE102708	---
---	69	118	**1.72**	BF390648	---
---	317	165	**0.52**	BI303277	---
---	46	97	**2.10**	AW528011	---
---	81	247	**3.03**	AI101416	---
---	53	120	**2.28**	AI071755	---
---	51	197	**3.91**	AI575277	---
---	107	70	**0.65**	AA998001	---
---	22	82	**3.66**	AI043711	---
---	321	167	**0.52**	AI555608	---
---	105	168	**1.60**	BE329364	---
---	96	61	**0.64**	AI013683	---
---	88	57	**0.64**	AW529368	---
---	168	109	**0.65**	AI029275	---
---	88	28	**0.32**	BI297481	---
---	12	95	**7.93**	AA893192	---
LOC49827	283	479	**1.69**	BE111722	---
---	260	129	**0.49**	AI169620	---
---	61	118	**1.94**	BM388957	---
---	168	302	**1.80**	AI012566	---
---	125	259	**2.08**	BE103242	Transcribed locus
LOC67981	63	148	**2.34**	AA963975	similar to GLI pathogenesis-related 2
---	78	117	**1.50**	**AW253907**	Transmembrane 9 superfamily protein member 4 (pred
LOC29131	310	474	**1.53**	AA900781	N-myristoyltransferase 2
