# Supplementary material for: Transgenerational Epigenetic Programming of the Brain Transcriptome and Anxiety Behavior
Source: PLoS One. 2008 Nov 18;3(11):e3745. doi: 10.1371/journal.pone.0003745 (PMC2581440; doi:10.1371/journal.pone.0003745)
Supplement: Table S2 — Transgenerational Male Amygdala Regulated Genes (0.13 MB PDF) [file pone.0003745.s004.pdf]

Table S2
Transgenerational Male Amygdala Regulated Genes

Apoptosis

Sample	F3-Cont	F3-Vinc	**Vin/Con**		
Gene Symbol	Raw	Raw	**Ratio**	Genbank	Gene Title
Apg12	91	52	**0.57**	BF563278	Autophagy-related 12 (yeast)
Acin1	64	97	**1.50**	BE116857	Apoptotic chromatin condensation inducer 1

Cell Cycle

Sample	F3-Cont	F3-Vinc	**Vin/Con**		
Gene Symbol	Raw	Raw	**Ratio**	Genbank	Gene Title
Cdc2l5	60	92	**1.53**	BF405622	Cell division cycle 2-like 5 (cholinesterase-relate
Sugt1	417	691	**1.66**	BI299197	SGT1, suppressor of G2 allele of SKP1 (S. cere
Kif5b	124	76	**0.61**	AA924572	kinesin family member 5B

Cytoskeleton-ECM

Sample	F3-Cont	F3-Vinc	**Vin/Con**		
Gene Symbol	Raw	Raw	**Ratio**	Genbank	Gene Title
Boc_predicted	100	50	**0.51**	BE110539	Biregional cell adhesion molecule-related/down-
Cdh22	77	25	**0.32**	BF388223	Cadherin 22
Cotl1_predicted	120	246	**2.04**	AI411057	coactosin-like 1 (Dictyostelium) (predicted)
Eml1	180	273	**1.52**	BI289642	Echinoderm microtubule associated protein like
Flnb_predicted	111	37	**0.34**	BI275447	Filamin, beta (predicted)
Kif5a	141	87	**0.62**	BF408765	Kinesin family member 5A
Mical2_predictec	78	47	**0.61**	BI274243	Microtubule associated monoxygenase, calponi
Map1b	150	85	**0.57**	BE096402	Microtubule-associated protein 1b
Map4	646	1046	**1.62**	AI230787	Microtubule-associated protein 4
Mapre1	232	131	**0.56**	U75920	microtubule-associated protein, RP/EB family, m
Opcml	534	822	**1.54**	M88709	Opioid binding protein/cell adhesion molecule-lil
RGD:619969	263	149	**0.56**	NM_020074	proteoglycan peptide core protein
**Pcdh7**	**138**	**79**	**0.57**	**BE121006**	**protocadherin 7///Transcribe locus**
RGD:621676	90	58	**0.65**	BI282702	smooth muscle alpha-actin
**LOC685664**	**142**	**76**	**0.53**	**BF406304**	**similar to axonemal dynein light chain 1**
Vezt	59	98	**1.67**	AI235367	Vezatin, adherens junctions transmembrane prc

Development

Sample	F3-Cont	F3-Vinc	**Vin/Con**		
Gene Symbol	Raw	Raw	**Ratio**	Genbank	Gene Title
Hnt	730	363	**0.50**	AA955579	neurotrimin
Apc	200	115	**0.57**	NM_012499	adenomatosis polyposis coli
Ash1l_predicted	157	100	**0.64**	BE119506	Ash1 (absent, small, or homeotic)-like (Drosoph
**Auts2_predicte**	**79**	**37**	**0.46**	**AI070144**	**Autism susceptibility candidate 2 (predicted)**
Cpne8_predictec	119	199	**1.68**	AI059204	Copine VIII (predicted)
Hrpt2_predicted	541	273	**0.50**	BI290578	hyperparathyroidism 2 (with jaw tumor) (predicte
Inexa	203	317	**1.56**	X52017	Internexin, alpha
Kirrel3_predictec	127	82	**0.65**	BF399121	kin of IRRE like 3 (Drosophila) (predicted)
Lmo3	556	214	**0.39**	AF353304	LIM domain only 3
LOC680445	92	53	**0.58**	AA875457	similar to muscleblind-like 2 isoform 1
Lrrtm3_predictec	79	40	**0.50**	BE107602	Leucine rich repeat transmembrane neuronal 3
Ndph_predicted	164	101	**0.62**	BF394149	Norrie disease homolog (human) (predicted)
Negr1	80	37	**0.46**	AW527711	Neuronal growth regulator 1
Nelf	906	1389	**1.53**	AJ293698	Nasal embryonic LHRH factor

Pmch	54	108	**1.99**	NM_012625	Pro-melanin-concentrating hormone
RGD1565646_pı	113	44	**0.39**	AI111954	Similar to SOX2 protein (predicted)
Sacs_predicted	109	61	**0.56**	BF567998	Sacsin (predicted)
**Sncg**	**368**	**837**	**2.28**		**Synuclein, gamma**
Sox9	812	352	**0.43**	AI454332	SRY-box containing gene 9
Tex264	134	204	**1.52**	AW252660	Testis expressed gene 264 homolog (mouse)

Golgi Apparatus

Sample	F3-Cont	F3-Vinc	**Vin/Con**		
Gene Symbol	Raw	Raw	**Ratio**	Genbank	Gene Title
Chst8_predicted	51	100	**1.97**	AI501232	Carbohydrate (N-acetylgalactosamine 4-0) sulfc
Gga3_predicted	166	104	**0.63**	BE112983	Golgi associated, gamma adaptin ear containinç
Hs2st1_predicte	79	48	**0.62**	AW530083	heparan sulfate 2-O-sulfotransferase 1 (predicte
Man1a2_predicte	121	73	**0.60**	BF401339	mannosidase, alpha, class 1A, member 2 (predi

Growth Factors and Cytokines

Sample	F3-Cont	F3-Vinc	**Vin/Con**		
Gene Symbol	Raw	Raw	**Ratio**	Genbank	Gene Title
Csf1	315	176	**0.56**	AW535553	colony stimulating factor 1(macrophage)
Fgfr1	197	318	**1.61**	BI275155	Fibroblast growth factor receptor 1
Fstl1	78	48	**0.62**	NM_024369	follistatin-like 1
Grem1	87	45	**0.52**	NM_019282	Gremlin 1 homolog, cysteine knot superfamily (>
Wnt4	68	136	**1.99**	NM_053402	wingless-type MMTV integration site family, mer

Immune Response

Sample	F3-Cont	F3-Vinc	**Vin/Con**		
Gene Symbol	Raw	Raw	**Ratio**	Genbank	Gene Title
Cdw92	91	58	**0.63**	AI105205	CDW92 antigen
Igsf4a	244	155	**0.64**	AA900645	immunoglobulin superfamily, member 4A
Irf5_predicted	77	126	**1.64**	BF284236	Interferon regulatory factor 5 (predicted)
RGD1306344	83	53	**0.63**	AI073208	Similar to Ab1-133
LOC690085	125	199	**1.60**	AW534002	Similar to B-cell CLL/lymphoma 7A
LOC680404	769	1215	**1.58**	BG374818	Similar to Complement C1q-like protein 3 precu
**Sart2_predicted**	**112**	**66**	**0.58**	**BM386930**	**Squamous cell carcinoma antigen recognize**
Tollip_predicted	87	51	**0.59**	BF408962	Toll interacting protein (predicted)

Metabolism

Sample	F3-Cont	F3-Vinc	**Vin/Con**		
Gene Symbol	Raw	Raw	**Ratio**	Genbank	Gene Title
Abcc1	107	71	**0.66**	AI059506	ATP-binding cassette, sub-family C (CFTR/MRF
Abhd8_predicted	246	164	**0.66**	AI137533	Abhydrolase domain containing 8 (predicted)
Adpgk	107	191	**1.79**	BE102146	ADP-dependent glucokinase
Agpat1	99	175	**1.76**	BI284306	1-acylglycerol-3-phosphate O-acyltransferase 1
Art3_predicted	113	70	**0.62**	AI104393	ADP-ribosyltransferase 3 (predicted)
Asph_predicted	127	71	**0.56**	AI113308	aspartate-beta-hydroxylase (predicted)
Atp1b2	239	99	**0.42**	U45946	ATPase, Na+/K+ transporting, beta 2 polypeptid
Atp2b1	184	300	**1.63**	AW534277	ATPase, Ca++ transporting, plasma membrane
Clstn2	54	119	**2.19**	BE109141	Calsyntenin 2
Comt	*293*	*182*	***0.62***	*NM_012531*	*catechol-O-methyltransferase*
Cyp2d22	162	107	**0.66**	U48220	Cytochrome P450, family 2, subfamily d, polype
Dpysl2	426	171	**0.40**	BE118404	dihydropyrimidinase-like 2
Entpd3	82	52	**0.63**	AI070096	Ectonucleoside triphosphate diphosphohydrolas
Folh1	77	48	**0.63**	AF040256	Folate hydrolase

Glul	793	462	**0.58**	BI296610	glutamine synthetase 1
Kcnj3	91	41	**0.46**	U09243	Potassium inwardly-rectifying channel, subfamily
Kcnk1	113	64	**0.57**	NM_021688	putative potassium channel TWIK
Kcnq3	141	88	**0.62**	BF400850	Potassium voltage-gated channel, subfamily Q,
Magi2	98	65	**0.66**	BF412454	Membrane associated guanylate kinase, WW ar
Nmt2	112	65	**0.58**	AA900781	N-myristoyltransferase 2
RGD1559938_pr	117	204	**1.74**	AI101062	Similar to spermine synthase (predicted)
Scn4b	202	128	**0.63**	AI137995	Sodium channel, voltage-gated, type IV, beta
Slc15a2	330	500	**1.52**	NM_031672	Solute carrier family 15 (H+/peptide transporter)
Slc27a4	177	101	**0.57**	BI286134	Solute carrier family 27 (fatty acid transporter), r
Slc4a11_predicte	107	64	**0.60**	BI293444	Solute carrier family 4, sodium bicarbonate trans
Sqle	187	286	**1.53**	NM_017136	Squalene epoxidase
Tmed5	57	99	**1.74**	AI137113	Transmembrane emp24 protein transport domai
Xylt2	84	49	**0.58**	AA892497	Xylosyltransferase II

Proteolysis

Sample	F3-Cont	F3-Vinc	**Vin/Con**		
Gene Symbol	Raw	Raw	**Ratio**	Genbank	Gene Title
Psmc3ip	81	50	**0.61**	NM_134458	proteasome (prosome, macropain) 26S subunit,
Rnf6_predicted	***31***	***77***	***2.46***	***BI296352***	***Ring finger protein (C3H2C3 type) 6 (predicte***
RGD1560364_pr	99	43	**0.44**	BE108174	Similar to vacuolar protein sorting 13C protein
Senp5_predictec	*57*	*186*	***3.23***	*BE105606*	*SUMO/sentrin specific protease 5 (predicted) ///*
Ube2n	350	636	**1.82**	BI294702	ubiquitin-conjugating enzyme E2N (homologous
Xpnpep1	223	338	**1.52**	AI178742	X-prolyl aminopeptidase (aminopeptidase P) 1,

Receptors & Binding proteins

Sample	F3-Cont	F3-Vinc	**Vin/Con**		
Gene Symbol	Raw	Raw	**Ratio**	Genbank	Gene Title
Dlgap2	87	45	**0.51**	BF409093	Discs, large (Drosophila) homolog-associated pr
Fnbp1	80	185	**2.32**	BI274481	Formin binding protein 1
Gabbr1	459	296	**0.65**	Y10369	Gamma-aminobutyric acid (GABA) B receptor 1
Gria1	138	208	**1.51**	BG376217	glutamate receptor, ionotropic, AMPA1 (alpha 1)
Lphn3	101	38	**0.38**	AF081159	Latrophilin 3
Plxna3	81	48	**0.59**	AW141867	Plexin A3 (mapped)
LOC685491	100	66	**0.65**	BF284235	Similar to retinoblastoma binding protein 4
Sort1	107	67	**0.62**	BF394904	Sortilin 1
Trpm3_predicted	116	71	**0.61**	BF413661	Transient receptor potential cation channel, sub

Signaling

Sample	F3-Cont	F3-Vinc	**Vin/Con**		
Gene Symbol	Raw	Raw	**Ratio**	Genbank	Gene Title
**Abca1**	**96**	**157**	**1.65**	**AI502114**	**ATP-binding cassette, sub-family A (ABC1), r**
Akap5	***182***	***60***	***0.33***	***NM_133515***	***A kinase (PRKA) anchor protein 5***
Anxa4	68	105	**1.55**	BM385237	ZAP 36/annexin IV
Arf6	110	73	**0.66**	NM_024152	ADP-ribosylation factor 6
Arhgap20	85	52	**0.61**	BF404613	Rho GTPase activating protein 20
Axin2	40	77	**1.92**	NM_024355	axin2
Bcar1	58	87	**1.50**	NM_012931	breast cancer anti-estrogen resistance 1
Camk2a	*331*	*934*	***2.82***	*BM384558*	*calcium/calmodulin-dependent protein kinase II*
Camk2a	176	116	**0.66**	BE107291	Calcium/calmodulin-dependent protein kinase II
Camk2d	155	69	**0.45**	X77194	calcium/calmodulin-dependent protein kinase II,
Dcamkl3_predict	91	46	**0.51**	AW529479	Doublecortin and CaM kinase-like 3 (predicted)
Dusp1	392	231	**0.59**	U02553	dual specificity phosphatase 1

Dyrk1a	85	48	**0.57**	BI303285	Dual-specificity tyrosine-(Y)-phosphorylation reg
Eps8_predicted /	108	72	**0.66**	BF393611	Epidermal growth factor receptor pathway subst
Faf1	165	254	**1.54**	NM_130406	Fas-associated factor 1
Gabrg3	119	75	**0.63**	BF403919	Gamma-aminobutyric acid (GABA) A receptor, s
Gng12	92	59	**0.65**	AI576549	Guanine nucleotide binding protein (G protein),
Gnl2	43	87	**2.03**	AI060050	Guanine nucleotide binding protein-like 2 (nucle
Gpr153	91	53	**0.58**	AI599549	G protein-coupled receptor 153
Gpr88	273	140	**0.51**	NM_031696	G-protein coupled receptor 88
Homer1	144	69	**0.48**	BF397258	Homer homolog 1 (Drosophila)
Hspa1a	96	56	**0.58**	BI278231	heat shock 70kD protein 1A
LOC686610	82	17	**0.21**	BF396263	similar to transducin (beta)-like 2
Plekhc1_predicte	93	50	**0.54**	BE128738	pleckstrin homology domain containing, family C
Ppp1r3c	189	115	**0.61**	AW530361	Protein phosphatase 1, regulatory (inhibitor) sub
Prkag2	202	345	**1.71**	AI409841	Protein kinase, AMP-activated, gamma 2 non-ca
Prkag2	200	120	**0.60**	AW527165	Protein kinase, AMP-activated, gamma 2 non-ca
Prkar1a	124	75	**0.60**	AW524136	protein kinase, cAMP dependent regulatory, typ
Prkcc	321	533	**1.66**	NM_012628	Protein kinase C, gamma
Prkg2	132	80	**0.61**	NM_013012	Protein kinase, cGMP-dependent, type II
Ptprd	77	49	**0.64**	BM392254	Protein tyrosine phosphatase, receptor type, D
Rab15	397	610	**1.54**	BM384841	RAB15, member RAS onocogene family
Rab3gap2	275	166	**0.60**	AI011183	RAB3 GTPase activating protein subunit 2
RGD1306588_pı	112	75	**0.67**	BF418962	Similar to TBC1 domain family member 22A
RGD1311455_pı	180	112	**0.62**	BE108260	Similar to MAPK-interacting and spindle-stabilizi
RGD1565261_pı	161	293	**1.82**	AI412533	similar to kinase non-catalytic C-lobe domain (K
Ripk2_predicted	88	50	**0.57**	BF412519	Receptor (TNFRSF)-interacting serine-threonine
Rspo1_predicted	92	56	**0.61**	AI044916	R-spondin homolog (Xenopus laevis) (predicted
Stk32c_predicted	495	291	**0.59**	BE097305	Serine/threonine kinase 32C (predicted)
Trio	360	204	**0.57**	AI577848	triple functional domain (PTPRF interacting)
---	132	87	**0.66**	AI070638	Transcribed locus, strongly similar to NP_73375

Transcription

Sample<br>Gene Symbol	F3-Cont<br>Raw	F3-Vinc<br>Raw	**Vin/Con**<br>**Ratio**	Genbank	Gene Title
**Ankrd12_predic**	**98**	**49**	**0.50**	**AI577496**	**ankyrin repeat domain 12 (predicted)**
Atf2	100	60	**0.60**	BF288181	Activating transcription factor 2
Baz2b_predicted	87	41	**0.47**	BF404567	Bromodomain adjacent to zinc finger domain, 2I
Bhlhb8	100	174	**1.75**	BE110870	basic helix-loop-helix domain containing, class E
Clasp1_predicted	81	53	**0.65**	AI044920	Chromodomain helicase DNA binding protein 4
Cugbp2	131	68	**0.52**	AW523660	CUG triplet repeat, RNA binding protein 2
Ddef1_predicted	137	83	**0.60**	AA894199	Development and differentiation enhancing (pre
Ddx3x	109	40	**0.37**	BG667163	DEAD (Asp-Glu-Ala-Asp) box polypeptide 3, X-l
Edg2	643	1002	**1.56**	NM_053936	endothelial differentiation, lysophosphatidic acid
Egr2	144	82	**0.57**	NM_053633	early growth response 2
Epc2_predicted	51	88	**1.74**	AI535147	Enhancer of polycomb homolog 2 (Drosophila) (
Fbxo33_predicte	114	174	**1.53**	AW918372	F-box only protein 33 (predicted)
Hdac1	79	44	**0.56**	AW530195	Histone deacetylase 1
Hint3	187	120	**0.64**	AA925490	Histidine triad nucleotide binding protein 3
Ing4	195	86	**0.44**	BM391761	Inhibitor of growth family, member 4
LOC500030	78	155	**1.99**	BE107536	similar to PHD finger protein 14 isoform 1
LOC685610	75	117	**1.56**	BF551093	similar to zinc finger protein 59
**LOC69142**	**78**	**46**	**0.60**	**BF396725**	**similar to zinc finger protein 101**
Mxi1	146	77	**0.53**	NM_013160	Max interacting protein 1
Ncor1	176	94	**0.53**	BF390024	nuclear receptor co-repressor 1

Nfib	389	631	**1.62**	AW253720	Nuclear factor I/B
Nfix	*1335*	*454*	***0.34***	*AI555855*	*Nuclear factor I/X*
Pbx1_predicted	76	32	**0.42**	BI281745	pre-B-cell leukemia transcription factor 1 (predic
Poldip3_predicte	418	272	**0.65**	BF420785	polymerase (DNA-directed), delta interacting pro
RGD1307526	83	157	**1.89**	BF397936	Similar to modulator of estrogen induced transc
RGD1310602_pr	138	87	**0.63**	BE105705	Similar to ankyrin repeat domain 40
Satb1	181	277	**1.53**	AI227638	Special AT-rich sequence binding protein 1
Stx1a	215	124	**0.58**	NM_053788	Syntaxin 1A (brain)
Tcf20_mapped	108	170	**1.57**	BF394639	Transcription factor 20 (mapped)
Tnks2_predicted	133	62	**0.47**	H32233	Tankyrase, TRF1-interacting ankyrin-related AD
Tsn	317	130	**0.41**	BM391661	translin
Wasl	358	553	**1.54**	AI706673	Wiskott-Aldrich syndrome-like (human)
Xab2	72	121	**1.68**	AF277899	XPA binding protein 2
Zbed3_predicted	89	55	**0.62**	BE110658	Zinc finger, BED domain containing 3
Zc3hdc7_predict	143	88	**0.62**	BE116569	Zinc finger CCCH type containing 7 A (predicted
Zcrb1	144	233	**1.62**	BF386158	Zinc finger CCHC-type and RNA binding motif 1
Zfhx1b_predicted	120	246	**2.05**	BG377397	Zinc finger homeobox 1b
Zfp533_predicted	71	120	**1.70**	AA818377	zinc finger protein 533 (predicted)
Zfpm2_predicted	39	80	**2.07**	AI175842	Zinc finger protein, multitype 2 (predicted)

Translation and Protein Modification

Sample<br>Gene Symbol	F3-Cont<br>Raw	F3-Vinc<br>Raw	**Vin/Con**<br>**Ratio**	Genbank	Gene Title
LOC678972	83	50	**0.61**	AA875047	Chaperonin subunit 6a (zeta) (predicted)
Eif5b	89	135	**1.52**	BF395978	Eukaryotic translation initiation factor 5B
Exosc5_predicte	73	113	**1.55**	AA943578	Exosome component 5 (predicted)
Qtrtd1_predicted	145	94	**0.65**	AA819409	queuine tRNA-ribosyltransferase domain contai
Rpl3	*875*	*1356*	***1.55***	*AW142765*	*ribosomal protein L3*
Trmt5_predicted	75	118	**1.57**	BF407592	TRM5 tRNA methyltransferase 5 homolog (S. ce

Miscel & Unknown

Gene Symbol	Raw	Raw	**Vin/Con**	Genbank	Gene Title
Sample<br>Gene Symbol	F3-Cont<br>Raw	F3-Vinc<br>Raw	**Vin/Con**<br>**Ratio**	Genbank	Gene Title
Gramd3	366	224	**0.61**	C06752	GRAM domain containing 3
LOC302898	218	340	**1.56**	BG377487	Ac1158
LOC315843	116	201	**1.72**	BE107185	similar to WD repeat domain 11 protein
LOC498014	106	61	**0.58**	BG379949	similar to hypothetical protein LOC284018 isofo
LOC500720 /// L	313	201	**0.64**	BG670822	Non-coding RNA expressed in the brain, repeat
LOC680229	253	424	**1.67**	AI413058	Similar to Beta-sarcoglycan (Beta-SG) (43 kDa
LOC685448	81	47	**0.58**	AI101017	Hypothetical protein LOC685448
LOC689176	183	120	**0.65**	AI103213	similar to transmembrane protein 64
Lsm14a_predicte	79	46	**0.59**	BF417956	LSM14 homolog A (SCD6, S. cerevisiae) (predic
Mdn1	83	32	**0.38**	AA956668	Midasin homolog (yeast)
Mterfd2	90	153	**1.71**	BI293662	MTERF domain containing 2
RGD1304810_pr	81	54	**0.66**	AI044301	similar to 6430573F11Rik protein (predicted)
RGD1305631_pr	77	47	**0.61**	BF420447	Similar to 2810437L13Rik protein (predicted)
RGD1305958_pr	114	75	**0.66**	BF408519	Similar to BB128963 protein
RGD1306798_pr	106	170	**1.61**	AI136405	similar to CG15929-PA (predicted)
RGD1307479_pr	84	126	**1.51**	BG372657	similar to KIAA1521 protein (predicted)
RGD1309995_pr	79	124	**1.56**	BF398054	Similar to CG13957-PA (predicted)
RGD1310117_pr	196	121	**0.61**	BG381331	Hypothetical LOC298591 (predicted)
RGD1310433_pr	152	89	**0.58**	AW254450	Similar to mKIAA1757 protein (predicted)

RGD1310651_pr	132	85	**0.64**	BF407551	similar to hypothetical protein MGC20460 (predi
RGD1311016_pr	118	65	**0.55**	BI281497	similar to RIKEN cDNA 9130427A09 (predicted)
RGD1312005_pr	224	137	**0.61**	BF392349	Similar to DD1 (predicted)
RGD1559605_p	***107***	***260***	***2.43***	***BE101933***	***similar to hypothetical protein FLJ25477 isof***
RGD1561931_pr	44	104	**2.38**	BF416137	Similar to KIAA2022 protein (predicted)
RGD1562278_pr	87	58	**0.66**	BF409055	Similar to KTSR5831 (predicted)
RGD1563912_pr	82	44	**0.54**	BG672771	RGD1563912 (predicted)
RGD1564084_pr	42	95	**2.24**	BM387858	Similar to timeless-interacting protein (predicted
RGD1564451_pr	75	47	**0.63**	BE114077	Similar to Tribbles homolog 2 (predicted)
RGD1565350_pr	101	59	**0.58**	BF283302	Similar to Shb protein
RGD1565498_pr	83	52	**0.62**	BF414751	Similar to Hypothetical protein LOC270802 (pre
RGD1565556_pr	78	17	**0.22**	BF404888	similar to cajalin 2 isoform a (predicted)
RGD1565596_pr	66	113	**1.70**	AI072448	Similar to Gene model 461 (predicted)
RGD1565884_pr	153	79	**0.52**	AI043817	similar to Pellino protein homolog 2 (Pellino 2) (
RGD1566117_pr	526	1101	**2.09**	AI555865	Similar to hypothetical protein FLJ23033 (predic
Ston2_predicted	100	220	**2.20**	AW534519	stonin 2 (predicted)
Trim33_predicted	130	48	**0.37**	AI103408	tripartite motif protein 33 (predicted)
Wdr66_predicted	48	78	**1.62**	BF283420	WD repeat domain 66 (predicted)

EST's

Sample	F3-Cont	F3-Vinc	**Vin/Con**		
Gene Symbol	Raw	Raw	**Ratio**	Genbank	Gene Title
**---**	**101**	**47**	**0.47**	**AA800192**	**Transcribed locus, strongly similar to XP_57**
---	154	243	**1.58**	AA996970	Transcribed locus, strongly similar to NP_95466
---	110	68	**0.62**	AI411809	Transcribed locus
---	80	37	**0.46**	BF290937	Transcribed locus
---	90	57	**0.63**	AI102735	Transcribed locus
	74	153	**2.07**	BI282726	Transcribed locus
---	127	211	**1.66**	BM384026	Transcribed locus
---	79	44	**0.56**	BI300887	Transcribed locus
---	71	115	**1.63**	BE107482	Transcribed locus
---	140	59	**0.43**	AW531363	Transcribed locus
---	141	218	**1.55**	BE105498	Transcribed locus
---	153	97	**0.64**	AW527767	Transcribed locus
---	138	72	**0.52**	BI278180	Transcribed locus
---	104	68	**0.66**	BI293639	Transcribed locus
---	83	43	**0.52**	BG663446	Transcribed locus
---	91	52	**0.57**	AA964975	Transcribed locus
---	242	366	**1.51**	BG380796	Transcribed locus
---	*79*	*126*	***1.60***	*AA926072*	*Transcribed locus*
---	88	146	**1.66**	BF412064	Transcribed locus
---	449	712	**1.58**	BG668719	Transcribed locus
---	169	63	**0.37**	AI102173	Transcribed locus
---	65	133	**2.04**	AW530415	Transcribed locus
---	97	63	**0.64**	BE102746	Transcribed locus
---	190	287	**1.51**	AW526127	Transcribed locus
---	171	95	**0.55**	AW914907	Transcribed locus
---	203	61	**0.30**	BF404508	Transcribed locus
---	77	48	**0.63**	AW522969	Transcribed locus
**---**	**86**	**42**	**0.48**	**BF403875**	**Transcribed locus**
---	293	187	**0.64**	BF391580	Transcribed locus
---	144	93	**0.65**	BF555100	Transcribed locus
---	163	251	**1.54**	BF289504	Transcribed locus

RGD621098	87	50	**0.57**	U92010	Similar to RIKEN cDNA D230025D16Rik
RGD1307396_pı	76	117	**1.54**	AW916327	Similar to RIKEN cDNA 6330406I15 (predicted)
RGD1310022	*8*	*93*	***12.39***	*AI172311*	*similar to RIKEN cDNA 2610204K14*
RGD1311358	80	46	**0.58**	AI012753	similar to RIKEN cDNA 2410017P07
RGD1565557_pı	217	134	**0.62**	AF023090	Similar to RIKEN cDNA 2010301N04 (predicted
RGD1564454_pı	132	85	**0.64**	BM390539	Similar to RIKEN cDNA 2010200O16 (predicted
RGD1308134_pı	77	116	**1.51**	BM390489	Similar to RIKEN cDNA 1110020A23 (predicted)
RGD1306284_pı	143	224	**1.56**	AA866227	Similar to RIKEN cDNA 1110005A03
RGD:735106	60	147	**2.45**	AI712625	Similar to RIKEN cDNA 0610011N22 gene
---	160	84	**0.52**	AW921250	---
---	76	37	**0.49**	BF390648	---
**---**	**84**	**50**	**0.59**	**BI278779**	**---**
---	120	207	**1.73**	AI030314	---
---	122	77	**0.63**	AA998001	---
---	246	159	**0.65**	BE110241	---

Note - The bolded genes are similar between male and female gene sets.
The italic genes are similar within the same sex between amygdala and hippocampus.
