# Supplementary material for: Transgenerational Epigenetic Programming of the Brain Transcriptome and Anxiety Behavior
Source: PLoS One. 2008 Nov 18;3(11):e3745. doi: 10.1371/journal.pone.0003745 (PMC2581440; doi:10.1371/journal.pone.0003745)
Supplement: Table S3 — Transgenerational Male Hippocampus Regulated Genes (0.11 MB PDF) [file pone.0003745.s005.pdf]

Table S3
Transgenerational Male Hippocampus Regulated Genes

Cell Cycle

Sample	F3-Cont	F3-Vinc	**Vin/Con**		
Gene Symb	Raw	Raw	**Ratio**	Genbank	Gene Title
Myo1b	78	47	**0.60**	NM_053986	Myosin Ib
Ogn_predic	106	53	0.50	AA945955	osteoglycin (predicted)
**Pcdhgc3**	**181**	**103**	**0.57**	**BE097805**	**Protocadherin gamma subfamily C, 3**
Rdx	99	66	0.67	BE113016	Radixin
Sostdc1	237	20	**0.08**	AA892798	Sclerostin domain containing 1

Development

Sample	F3-Cont	F3-Vinc	**Vin/Con**		
Gene Symb	Raw	Raw	**Ratio**	Genbank	Gene Title
Chrdl1	103	67	**0.65**	AA819658	Chordin-like protein 1 precursor (Neuralin-1) (Ventrop
**Crim1_pre**	**128**	**221**	**1.72**	**BI289620**	**Cysteine-rich motor neuron 1 (predicted)**
Kl	284	14	**0.05**	BE102060	Klotho

Golgi Apparatus

Sample	F3-Cont	F3-Vinc	**Vin/Con**		
Gene Symb	Raw	Raw	**Ratio**	Genbank	Gene Title
Ap3m1	79	51	**0.65**	BF564312	Adaptor-related protein complex 3, mu 1 subunit

Growth Factors and Cytokines

Sample	F3-Cont	F3-Vinc	**Vin/Con**		
Gene Symb	Raw	Raw	**Ratio**	Genbank	Gene Title
Mdk	356	209	**0.59**	NM_030859	Midkine
**Tgfb2**	**90**	**54**	**0.60**	**BE117736**	**Transforming growth factor, beta 2**

Immune Response

Sample	F3-Cont	F3-Vinc	**Vin/Con**		
Gene Symb	Raw	Raw	**Ratio**	Genbank	Gene Title
F5	124	14	**0.11**	AI717113	Coagulation factor 5 (mapped)

Metabolism

Sample	F3-Cont	F3-Vinc	**Vin/Con**		
Gene Symb	Raw	Raw	**Ratio**	Genbank	Gene Title
Abhd14b	102	67	**0.66**	BF409560	Abhydrolase domain containing 14b
Acaa2	115	186	**1.62**	NM_130433	acetyl-Coenzyme A acyltransferase 2 (mitochondrial 3
**Acsl6**	**95**	**60**	**0.63**	**BE105421**	**acyl-CoA synthetase long-chain family member 6**
**Arg2**	**53**	**122**	**2.30**	**AI574994**	**Arginase 2**
Bcat1	855	468	**0.55**	NM_017253	Branched chain aminotransferase 1, cytosolic
**Ptgds**	**2848**	**1566**	**0.55**	**J04488**	**prostaglandin D2 synthase**
Comt	*324*	*149*	***0.46***	*NM_012531*	*catechol-O-methyltransferase*
Clic6	117	19	**0.16**	BI290737	Chloride intracellular channel 6
Cbs	108	186	**1.73**	NM_012522	Cystathionine beta synthase
Enpp2	1261	532	**0.42**	NM_057104	Ectonucleotide pyrophosphatase/phosphodiesterase 2
Fntb	155	97	**0.63**	M69056	Farnesyltransferase, CAAX box, beta
Qpctl_predi	57	87	**1.52**	AW521959	Glutaminyl-peptide cyclotransferase-like (predicted)
Hsd17b7	82	51	**0.62**	NM_017235	Hydroxysteroid (17-beta) dehydrogenase 7
Pex11b	170	257	**1.52**	BG377340	Peroxisomal biogenesis factor 11b
Pggt1b	169	110	**0.65**	AA875190	protein geranylgeranyltransferase type I, beta subunit

Slc13a4	76	30	**0.40**	AI013778	solute carrier family 13 (sodium/sulfate symporters), r
Slc31a1	100	61	**0.61**	NM_133600	Solute carrier family 31 (copper transporters), membe

Proteolysis

Sample	F3-Cont	F3-Vinc	**Vin/Con**		
Gene Symb	Raw	Raw	**Ratio**	Genbank	Gene Title
Senp5_pre	*62*	*181*	***2.92***	*BE105606*	*SUMO/sentrin specific protease 5 (predicted) /// simila*
**Wsb2**	**112**	**203**	**1.80**	**AA956464**	**WD SOCS-box protein 2**
Rnf6_pred	***42***	***108***	***2.60***	***BI296352***	***Ring finger protein (C3H2C3 type) 6 (predicted)***

Receptors & Binding Proteins

Sample	F3-Cont	F3-Vinc	**Vin/Con**		
Gene Symb	Raw	Raw	**Ratio**	Genbank	Gene Title
Folr1	131	40	**0.31**	AI233882	Folate receptor 1 (adult)
Gmeb2	131	74	**0.57**	AF205779	Glucocorticoid modulatory element binding protein 2
Hrh3	151	237	**1.58**	NM_053506	Histamine receptor H3
**Igfbp2**	**266**	**118**	**0.44**	**NM_013122**	**Insulin-like growth factor binding protein 2**
Ifngr2_pred	94	63	**0.67**	AI602715	Interferon gamma receptor 2 (predicted)
**Stxbp1**	**207**	**128**	**0.62**	**U06069**	**Syntaxin binding protein 1**
Ttr	4426	103	**0.02**	NM_012681	Transthyretin
Leprot	76	50	**0.66**	NM_020099	leptin receptor overlapping transcript

Signaling

Sample	F3-Cont	F3-Vinc	**Vin/Con**		
Gene Symb	Raw	Raw	**Ratio**	Genbank	Gene Title
**Agtpbp1_p**	**199**	**125**	**0.63**	**BF553179**	**ATP/GTP binding protein 1 (predicted)**
Akap5	*113*	*59*	***0.52***	*NM_133515*	*A kinase (PRKA) anchor protein 5*
Camk2a	***300***	***865***	***2.88***	***BM384558***	***calcium/calmodulin-dependent protein kinase II al***
Cib2	127	78	**0.62**	BF403998	Calcium and integrin binding family member 2
**Cit**	**106**	**171**	**1.61**	**AA957183**	**Citron;citron kinase**
Hspa12a_p	490	765	**1.56**	BG670210	Heat shock 70kDa protein 12A (predicted)
Nek1_predi	98	49	**0.50**	AI406369	NIMA (never in mitosis gene a)-related expressed kin
Pde9a	80	157	**1.97**	BF399743	Phosphodiesterase 9A
Pfkm	472	312	**0.66**	BI291434	Phosphofructokinase, muscle
**Plek2_prec**	**190**	**72**	**0.38**	**AI102821**	**pleckstrin 2 (predicted)**
**Plekhh1_pı**	**90**	**34**	**0.38**	**BI275435**	**pleckstrin homology domain containing, family H**
Rab3b	131	197	**1.51**	NM_031091	RAB3B, member RAS oncogene family
RGD15616	117	68	**0.58**	AA859909	Similar to TBC1 domain family, member 5 (predicted)

Transcription

Sample	F3-Cont	F3-Vinc	**Vin/Con**		
Gene Symb	Raw	Raw	**Ratio**	Genbank	Gene Title
**Dnaja4**	**97**	**65**	**0.67**	**BF565278**	**DnaJ (Hsp40) homolog, subfamily A, member 4**
Fos	97	58	**0.60**	BF415939	FBJ murine osteosarcoma viral oncogene homolog
Ndrg1_prec	86	55	**0.64**	BE120446	N-myc downstream regulated gene 1
Nfix	***2495***	***1610***	***0.65***	***AI555855***	***Nuclear Factor I/X***
**Nrip3_pred**	**342**	**209**	**0.61**	**BI288541**	**Nuclear receptor interacting protein 3 (predicted)**
**Rnf187_pre**	**131**	**85**	**0.65**	**AI502527**	**Ring finger protein 187 (predicted)**
**LOC68537**	**156**	**101**	**0.64**	**BG671371**	**Similar to ankyrin repeat domain 13c**
Smc5l1_pre	77	46	**0.60**	BF419367	SMC5 structural maintenance of chromosomes 5-like

Translation

Sample	F3-Cont	F3-Vinc	**Vin/Con**		
Gene Symb	Raw	Raw	**Ratio**	Genbank	Gene Title
Rae1	78	44	**0.56**	BF419215	mRNA-associated protein mrnp 41 (Rae1 protein hon
LOC49842	190	111	**0.58**	AW527413	Similar to U2 small nuclear ribonucleoprotein auxiliar

Miscellaneous & Unknown

Sample	F3-Cont	F3-Vinc	**Vin/Con**		
Gene Symb	Raw	Raw	**Ratio**	Genbank	Gene Title
**LOC68137**	**61**	**224**	**3.66**	**AA944136**	**hypothetical protein LOC681371**
Sema3c_pr	200	130	**0.65**	BM390322	sema domain, immunoglobulin domain (Ig), short bas
**RGD13119**	**277**	**530**	**1.92**	**BF563206**	**Similar to 6430514L14Rik protein (predicted)**
LOC50067	93	56	**0.60**	BM384427	Similar to B0511.12
RGD15596	***104***	***244***	***2.36***	***BE101933***	***similar to hypothetical protein FLJ25477 isoform***
LOC30991	76	47	**0.62**	BM385683	Similar to NOGO-interacting mitochondrial protein (pr
**Tmem24**	**332**	**512**	**1.54**	**BG378195**	**Transmembrane protein 24**
Tmem38a_	105	65	**0.62**	BF410357	Transmembrane protein 38a (predicted)
Vps4a	89	55	**0.61**	BF388609	Vacuolar protein sorting 4a (yeast)

EST's

Sample	F3-Cont	F3-Vinc	**Vin/Con**		
Gene Symb	Raw	Raw	**Ratio**	Genbank	Gene Title
---	574	366	0.64	AI028809	Similar to cDNA sequence BC006662 (predicted)
**LOC36262**	**211**	**127**	**0.60**	**BE096504**	**Similar to chromosome 1 open reading frame 63**
RGD13102	262	175	**0.67**	AI137602	similar to chromosome 7 open reading frame 19; CAF
RGD13084	349	556	**1.59**	AW521378	Similar to RIKEN cDNA B130016O10 gene (predicted
**---**	**632**	**295**	**0.47**	**AI179665**	**Transcribed locus**
---	82	54	**0.66**	AI044117	Transcribed locus
---	300	460	**1.53**	AA858791	Transcribed locus
**---**	**96**	**56**	**0.59**	**BF403869**	**Transcribed locus**
---	*691*	*1326*	***1.92***	*AW142765*	*Transcribed locus*
RGD13100	***7***	***135***	***19.42***	***AI172311***	***Transcribed locus***
---	79	133	**1.69**	AI146055	Transcribed locus
**---**	**323**	**603**	**1.87**	**AI112113**	**Transcribed locus**
---	89	138	**1.56**	BM385137	Transcribed locus
**---**	**145**	**360**	**2.49**	**AI103530**	**Transcribed locus**
---	112	60	**0.54**	BF386949	Transcribed locus
**---**	**138**	**217**	**1.58**	**AA925807**	**Transcribed locus**
---	*76*	*114*	***1.51***	*AA926072*	*Transcribed locus*
---	87	57	**0.65**	BE117189	Transcribed locus
---	139	68	**0.49**	BF403401	Transcribed locus, moderately similar to XP_580018.
RGD13098	125	75	**0.60**	BI288367	Transcribed locus, strongly similar to XP_001065048.

Note - The bolded genes are similar between male and female gene sets.
The italic genes are similar within the same sex between amygdala and hippocampus.
