# Supplementary material for: Transgenerational Epigenetic Programming of the Brain Transcriptome and Anxiety Behavior
Source: PLoS One. 2008 Nov 18;3(11):e3745. doi: 10.1371/journal.pone.0003745 (PMC2581440; doi:10.1371/journal.pone.0003745)
Supplement: Table S4 — Transgenerational Female Amygdala Regulated Genes (0.12 MB PDF) [file pone.0003745.s006.pdf]

Table S4
Transgenerational Female Amygdala Regulated Genes

Cytoskeleton-ECM

Sample	F3-Cont	F3-Vinc	**Vin/Con**		
Gene Symb	Raw	Raw	**Ratio**	Genbank	Gene Title_Affymetrix
Ncam1	144	92	**0.64**	BF409530	Neural cell adhesion molecule 1
Spock3_pre	357	236	**0.66**	BG668764	sparc/osteonectin, cwcv and kazal-like domains proteoglyc
Eml2	*70*	*110*	***1.58***	*AF335571*	*echinoderm microtubule associated protein like 2*
Fbln2	72	125	**1.73**	AA944398	fibulin 2
Kifap3_pre	80	52	**0.65**	BF418109	Kinesin-associated protein 3 (predicted)
Tpm3	278	108	**0.39**	NM_057208	tropomyosin 3, gamma
Pigl	79	51	**0.64**	D88364	phosphatidylinositol glycan, class L

Development

Sample	F3-Cont	F3-Vinc	**Vin/Con**		
Gene Symb	Raw	Raw	**Ratio**	Genbank	Gene Title_Affymetrix
**Auts2_pre**	**139**	**60**	**0.43**	**AI070144**	**autism susceptibility candidate 2 (predicted)**
Bai3_predic	81	52	**0.64**	BF401684	Brain-specific angiogenesis inhibitor 3 (predicted)
Crim1_prec	425	644	**1.52**	AI703807	cysteine-rich motor neuron 1 (predicted)
Dlgap1	91	23	**0.25**	BF413506	Discs, large (Drosophila) homolog-associated protein 1
Lrrc4	239	116	**0.48**	BE105879	leucine rich repeat containing 4 protein precursor
RGD15611	207	128	**0.62**	BF397988	Similar to autism susceptibility candidate 2 (predicted)
Sncg	*374*	*653*	***1.74***	*NM_031688*	*synuclein, gamma*
Slit3	138	88	**0.64**	NM_031321	slit homolog 3 (Drosophila)

Epigenetics

Sample	F3-Cont	F3-Vinc	**Vin/Con**		
Gene Symb	Raw	Raw	**Ratio**	Genbank	Gene Title_Affymetrix
Cav2	126	57	**0.45**	BE349669	caveolin 2
Chd2_pred.	*94*	*56*	***0.59***	*BF396633*	*Chromodomain helicase DNA binding protein 2 (predicted)*
Hp1bp3	130	76	**0.59**	BF396757	Heterochromatin protein 1, binding protein 3
LOC68292	*97*	*147*	***1.52***	*BF557618*	*similar to chromatin modifying protein 1B*

Golgi Apparatus

Sample	F3-Cont	F3-Vinc	**Vin/Con**		
Gene Symb	Raw	Raw	**Ratio**	Genbank	Gene Title_Affymetrix
RGD15605	*94*	*143*	***1.52***	*BI281965*	*similar to Vps41 protein (predicted)*
Pgea1	59	92	**1.55**	BE117101	PKD2 interactor, golgi and endoplasmic reticulum associat

Growth Factors,Cyto- and Chemokines

Sample	F3-Cont	F3-Vinc	**Vin/Con**		
Gene Symb	Raw	Raw	**Ratio**	Genbank	Gene Title_Affymetrix
Cmkor1	176	99	**0.56**	NM_053352	chemokine orphan receptor 1
Dock9	98	59	**0.60**	BI286269	dedicator of cytokinesis 9
Tgfb2	*263*	*150*	***0.57***	*BE117736*	*Transforming growth factor, beta 2*
Negr1	169	77	**0.45**	AW533779	Neuronal growth regulator 1

Immune Response

Sample	F3-Cont	F3-Vinc	**Vin/Con**		
Gene Symb	Raw	Raw	**Ratio**	Genbank	Gene Title_Affymetrix
Aif1	103	155	**1.51**	NM_017196	allograft inflammatory factor 1

RT1-S3	*83*	*41*	***0.49***	*AJ243974*	*RT1 class Ib, locus S3*
**Sart2_prec**	**102**	**63**	**0.62**	**BM386930**	**Squamous cell carcinoma antigen recognized by T cell**
Xpa_predic	59	89	**1.50**	BF554085	xeroderma pigmentosum, complementation group A (predic
RT1-Ke4	*196*	*122*	***0.62***	*BM389027*	*RT1 class I, locus Ke4*
Stag1_prec	*103*	*56*	***0.55***	*AI071210*	*Stromal antigen 1 (predicted)*

Metabolism & Transport

Sample	F3-Cont	F3-Vinc	**Vin/Con**		
Gene Symb	Raw	Raw	**Ratio**	Genbank	Gene Title_Affymetrix
Adk	123	188	**1.52**	U90340	adenosine kinase
Aqp4	*66*	*127*	***1.93***	*U14007*	*aquaporin 4*
Arcn1	*73*	*113*	***1.54***	*BF414061*	*archain 1*
Ca3	*29*	*297*	***10.14***	*AB030829*	*carbonic anhydrase 3*
Chac1_pre	140	92	**0.65**	AI170665	ChaC, cation transport regulator-like 1 (E. coli) (predicted)
Cth	56	87	**1.55**	NM_017074	cystathionase (cystathionine gamma-lyase)
Kcnh1	98	37	**0.38**	BF394600	Potassium voltage-gated channel, subfamily H (eag-relatec
LOC68480	112	59	**0.52**	BI273855	similar to Probable phospholipid-transporting ATPase ID (A
Pgm1	121	202	**1.66**	NM_017033	phosphoglucomutase 1
Pus7_predi	91	45	**0.49**	BM390168	Pseudouridylate synthase 7 homolog (S. cerevisiae) (predi
Scd2	*289*	*471*	***1.63***	*BE107760*	*stearoyl-Coenzyme A desaturase 2*

Proteolysis

Sample	F3-Cont	F3-Vinc	**Vin/Con**		
Gene Symb	Raw	Raw	**Ratio**	Genbank	Gene Title_Affymetrix
Lap3	164	103	**0.63**	AA945172	leucine aminopeptidase 3
RGD15638	269	163	**0.60**	AI180403	similar to cullin 4A (predicted)
Rnf6_pred	***24***	***99***	***4.09***	***BI296352***	***ring finger protein (C3H2C3 type) 6 (predicted)***
RGD15607	170	101	**0.60**	BF396481	Similar to ring finger protein 111 (predicted)
Usp47_pre	*82*	*137*	***1.68***	*AI407830*	*ubiquitin specific protease 47 (predicted)*

Receptors & Binding Proteins

Sample	F3-Cont	F3-Vinc	**Vin/Con**		
Gene Symb	Raw	Raw	**Ratio**	Genbank	Gene Title_Affymetrix
Abca1	***76***	***174***	***2.30***	***AI502114***	***ATP-binding cassette, sub-family A (ABC1), member 1***
Abca3	77	116	**1.51**	BF546340	ATP-binding cassette, sub-family A (ABC1), member 3
Gabbr1	86	56	**0.65**	BF410498	Gamma-aminobutyric acid (GABA) B receptor 1

Signaling

Sample	F3-Cont	F3-Vinc	**Vin/Con**		
Gene Symb	Raw	Raw	**Ratio**	Genbank	Gene Title_Affymetrix
**Akap5**	**215**	**102**	**0.48**	**NM_133515**	**A kinase (PRKA) anchor protein 5**
Cnksr3	80	52	**0.65**	AW253242	Cnksr family member 3
Dusp8_pre	83	48	**0.58**	AW528387	dual specificity phosphatase 8 (predicted)
Farp1_pred	48	83	**1.73**	AI058490	FERM, RhoGEF (Arhgef) and pleckstrin domain protein 1 (
Git2	123	69	**0.56**	BF402645	G protein-coupled receptor kinase-interactor 2
Grik5	100	60	**0.60**	BF404569	Glutamate receptor, ionotropic, kainate 5
Herpud1	293	191	**0.65**	NM_053523	homocysteine-inducible, endoplasmic reticulum stress-indu
Itfg3	108	69	**0.64**	AA799854	integrin alpha FG-GAP repeat containing 3
Itgb8_predi	193	114	**0.59**	BG668993	Integrin beta 8 (predicted)
Itgb8_predi	362	204	**0.56**	AI502837	Integrin beta 8 (predicted)
LOC68444	144	265	**1.84**	BE106252	similar to Peptidyl-prolyl cis-trans isomerase NIMA-interact
Nek1_predi	126	76	**0.61**	AI406369	NIMA (never in mitosis gene a)-related expressed kinase 1
Ntrk2	122	74	**0.61**	BF386266	Neurotrophic tyrosine kinase, receptor, type 2

Plekhb2_pr	*41*	*88*	***2.13***	*AW254369*	*pleckstrin homology domain containing, family B (evectins)*
Ppp2r1b	58	106	**1.84**	AI411788	protein phosphatase 2 (formerly 2A), regulatory subunit A (
Ppp3ca	282	146	**0.52**	AI145507	Protein phosphatase 3, catalytic subunit, alpha isoform
Ppp3ca	*69*	*112*	***1.63***	*BF388224*	*Protein phosphatase 3, catalytic subunit, alpha isoform*
Ppp6c	102	158	**1.56**	NM_133589	protein phosphatase 6, catalytic subunit
Prkce	*101*	*45*	***0.45***	*BI301465*	*Protein kinase C, epsilon*
Prkwnk1	138	74	**0.54**	AI714037	Protein kinase, lysine deficient 1
Ptprj	*376*	*182*	***0.48***	*NM_017269*	*protein tyrosine phosphatase, receptor type, J*
Ralgds	181	119	**0.66**	NM_019250	ral guanine nucleotide dissociation stimulator
RGD13072	85	44	**0.52**	BF391396	Similar to protein kinase, lysine deficient 1; kinase deficient
RGD15616	110	49	**0.44**	AW534457	Similar to nemo like kinase (predicted)
RICS_pred	*758*	*489*	***0.65***	*BE097238*	*RhoGAP involved in beta-catenin-N-cadherin and NMDA re*

Transcription

Sample	F3-Cont	F3-Vinc	**Vin/Con**		
Gene Symb	Raw	Raw	**Ratio**	Genbank	Gene Title_Affymetrix
Baz1b	111	63	**0.57**	BF395914	Bromodomain adjacent to zinc finger domain protein 1B
Bcl11b_pre	513	227	**0.44**	BM390227	B-cell leukemia/lymphoma 11B (predicted)
Foxo1a	128	85	**0.66**	BF406350	Forkhead box O1A
Klf5	63	104	**1.66**	NM_053394	Kruppel-like factor 5
LOC50043(	56	90	**1.61**	BF391635	similar to ankyrin repeat domain 6
LOC69066	91	60	**0.67**	BF398283	Similar to zinc finger protein 40
Mll	518	307	**0.59**	BE114473	myeloid/lymphoid or mixed-lineage leukemia
Mllt10	82	50	**0.60**	BF396749	myeloid/lymphoid or mixed-lineage leukemia (trithorax hom
NIPBL	104	61	**0.59**	AA963592	Nipped-B homolog (Drosophila)
RGD15631	*1396*	*2493*	***1.79***	*BE104219*	*similar to MADS box transcription enhancer factor 2, polype*
Wiz_predic	131	70	**0.53**	H31790	widely-interspaced zinc finger motifs (predicted)
Crebl2	*93*	*233*	***2.51***	*BE102391*	*cAMP responsive element binding protein-like 2*

Translation & Protein Modification

Sample	F3-Cont	F3-Vinc	**Vin/Con**		
Gene Symb	Raw	Raw	**Ratio**	Genbank	Gene Title_Affymetrix
Arf4l_predi	78	28	**0.36**	AI030650	ADP-ribosylation factor 4-like (predicted)
Brunol4_pr	*195*	*117*	***0.60***	*AW524497*	*Bruno-like 4, RNA binding protein (Drosophila) (predicted)*
Eif3s6ip	89	146	**1.64**	BF420467	eukaryotic translation initiation factor 3, subunit 6 interactin

Miscellaneous & Unknown

Sample	F3-Cont	F3-Vinc	**Vin/Con**		
Gene Symb	Raw	Raw	**Ratio**	Genbank	Gene Title_Affymetrix
Gramd1b_p	117	78	**0.67**	AI175700	GRAM domain containing 1B (predicted)
Lce1f_pred	176	98	**0.56**	BI281143	late cornified envelope 1F (predicted) /// RGD1561089 (pre
LOC31367	78	47	**0.60**	BF415778	Similar to CG11206-PA
LOC68333	78	49	**0.63**	AI058900	hypothetical protein LOC683334 /// hypothetical protein LO
LOC69077	42	101	**2.39**	BF404935	Similar to RUN and FYVE domain-containing 2
Qser1_pre	135	87	**0.64**	AA800519	Glutamine and serine rich 1 (predicted)
RGD13061	78	46	**0.59**	AI071962	similar to predicted CDS, putative protein of bilaterial origin
RGD13065	140	91	**0.65**	AI577870	similar to Protein C22orf5
RGD13079	*148*	*88*	***0.60***	*AI146080*	*similar to hypothetical protein FLJ14681 (predicted)*
RGD13087	89	51	**0.58**	BE107410	similar to KIAA0892 protein (predicted)
RGD15596	***195***	***383***	***1.96***	***BE101933***	***Similar to hypothetical protein FLJ25477 isoform 2 (pre***
RGD15619	489	238	**0.49**	AA848540	Similar to IQ motif and WD repeats 1 (predicted)
RGD15624	*112*	*174*	***1.56***	*BG665671*	*similar to WAC (predicted)*
RGD15644	220	370	**1.68**	AW915035	RGD1564450 (predicted)

Serinc3	83	50	**0.60**	BE120228	Serine incorporator 3
---	80	47	**0.59**	BE108371	Transcribed locus, weakly similar to XP_341406.2 similar t
---	153	90	**0.59**	BF544149	Transcribed locus, strongly similar to XP_001058170.1 hy
---	104	66	**0.64**	BF408769	Transcribed locus, strongly similar to XP_347025.2 hypoth
---	*396*	*257*	***0.65***	*AI029275*	*Transcribed locus, weakly similar to XP_001054001.1 sim*

EST's

Sample	F3-Cont	F3-Vinc	**Vin/Con**		
Gene Symb	Raw	Raw	**Ratio**	Genbank	Gene Title_Affymetrix
RGD15609	*6*	*178*	***27.85***	*AA799328*	*similar to expressed sequence AW413625 (predicted)*
RGD13118	*78*	*119*	***1.53***	*BI284801*	*similar to RIKEN cDNA 1110021N07*
RGD13053	77	50	**0.65**	BF401603	similar to RIKEN cDNA 4921511I16
RGD13057	*511*	*318*	***0.62***	*AI179665*	*Similar to RIKEN cDNA 5033406L14*
RGD13060	83	36	**0.43**	AW916721	similar to RIKEN cDNA A630054L15; hypothetical protein M
RGD13109	103	55	**0.54**	BE116091	similar to RIKEN cDNA E130308A19 (predicted)
---	207	130	**0.63**	BE103273	Transcribed locus
---	***89***	***152***	***1.70***	***BF406304***	***Transcribed locus***
**---**	**232**	**139**	**0.60**	**BE121006**	**Transcribed locus**
**---**	**141**	**47**	**0.33**	**BF403875**	**Transcribed locus**
**---**	**137**	**85**	**0.62**	**BI278779**	**Transcribed locus**
---	*172*	*86*	***0.50***	*AI102821*	*Transcribed locus*
---	*160*	*84*	***0.52***	*AI070489*	*Transcribed locus*
---	*154*	*351*	***2.27***	*AI103530*	*Transcribed locus*
---	231	139	**0.60**	BI278952	Transcribed locus
---	*109*	*299*	***2.76***	*AA944136*	*Transcribed locus*
---	*63*	*106*	***1.70***	*BE110067*	*Transcribed locus*
---	*76*	*137*	***1.79***	*AA899937*	*Transcribed locus*
**---**	**84**	**50**	**0.59**	**BF396725**	**Transcribed locus**
---	*120*	*200*	***1.66***	*BF545930*	*Transcribed locus*
---	65	106	**1.63**	BE120930	Transcribed locus
---	88	47	**0.54**	BG672252	Transcribed locus
---	109	71	**0.65**	BI286900	Transcribed locus
---	402	645	**1.60**	AI575082	Transcribed locus
---	104	36	**0.35**	BE097725	Transcribed locus
---	137	82	**0.60**	BF402566	Transcribed locus
---	*180*	*101*	***0.56***	*BF399309*	*Transcribed locus*
---	*82*	*52*	***0.64***	*BF391128*	*Transcribed locus*
---	200	101	**0.50**	AA996491	Transcribed locus
---	78	43	**0.55**	BF547003	Transcribed locus
---	*243*	*150*	***0.62***	*AA926109*	*Transcribed locus*
---	*120*	*78*	***0.66***	*AA859319*	*Transcribed locus*
---	115	70	**0.60**	AW528823	Transcribed locus
---	*212*	*122*	***0.58***	*AI145015*	*Transcribed locus*
---	*120*	*72*	***0.60***	*AI137306*	*Transcribed locus*
---	361	211	**0.58**	BE107458	Transcribed locus
---	80	42	**0.52**	BF418643	Transcribed locus
---	91	59	**0.65**	BF409564	Transcribed locus
---	112	66	**0.59**	BF394244	Transcribed locus
---	*113*	*75*	***0.67***	*AI101372*	*Transcribed locus*
---	133	201	**1.52**	AA944179	Transcribed locus
---	122	80	**0.65**	AW535602	---
**---**	**76**	**44**	**0.57**	**AA800192**	**---**
---	117	53	**0.46**	FX_ratb1/X12	---

---	80	46	**0.57**	AA875617	---
**---**	**137**	**79**	**0.57**	**AI577496**	**---**
---	765	400	**0.52**	NM_053584	---
---	100	152	**1.52**	AI764288	---
---	153	97	**0.63**	AI236099	---
---	350	233	**0.66**	BF388125	---
---	107	47	**0.44**	H31323	---
---	77	29	**0.38**	BF405788	---
---	203	341	**1.68**	BG671050	---
---	90	51	**0.57**	BF564798	---
---	78	41	**0.53**	AW919386	---
---	*38*	*78*	***2.06***	*BI288579*	*---*
---	231	153	**0.66**	BG380566	---
---	154	96	**0.62**	BF399576	---
---	301	169	**0.56**	AI176695	---
---	394	246	**0.63**	AI059929	---
---	3571	2335	**0.65**	AI008646	---
---	*174*	*100*	***0.57***	*BF410240*	*---*
---	82	39	**0.48**	AI013683	---

Note - The bolded genes are similar between male and female gene sets.
The italic genes are similar within the same sex between amygdala and hippocampus.
