# Supplementary material for: Transgenerational Epigenetic Programming of the Brain Transcriptome and Anxiety Behavior
Source: PLoS One. 2008 Nov 18;3(11):e3745. doi: 10.1371/journal.pone.0003745 (PMC2581440; doi:10.1371/journal.pone.0003745)
Supplement: Figure S1 — (8.19 MB PDF) [file pone.0003745.s001.pdf]

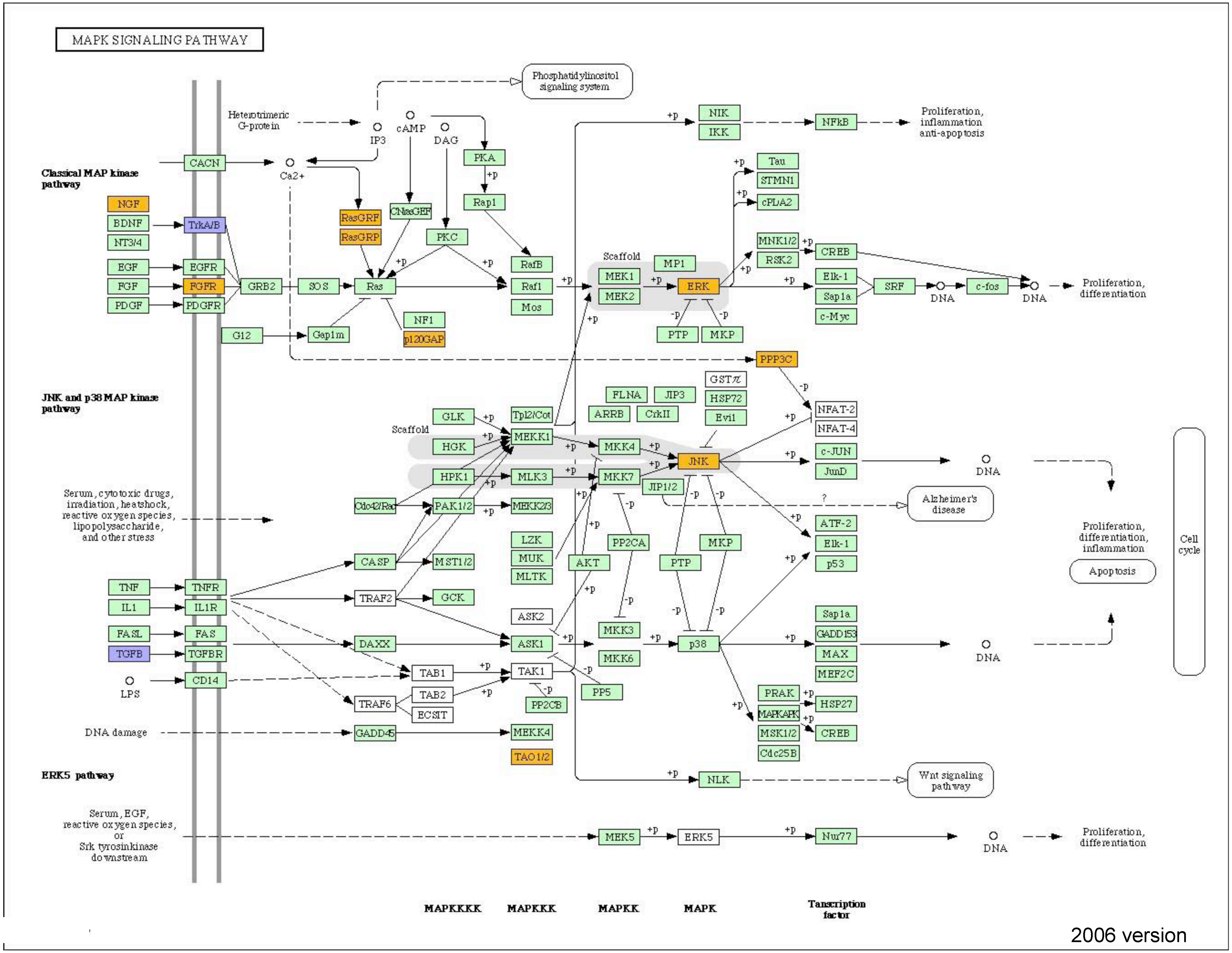

Supplemental Figure S1: Standard KEGG MAPK pathway of 25 altered genes in the F3 generation female hippocampus with red/orange indicating an increase in expression and blue a decrease in expression with green being no change in expression and white gene absent from the array. The genes in the MAPK pathway altered for the other tissue are as follows: male amygdala (aAf2, Prkca, Duspn1, Fgfr2, Gng12, FLNA); male hippocampus (Tgfb3, cfos); and female amygdala (Tgfb2, PPP3Ca)
