# Supplementary material for: Transgenerational Epigenetic Programming of the Brain Transcriptome and Anxiety Behavior
Source: PLoS One. 2008 Nov 18;3(11):e3745. doi: 10.1371/journal.pone.0003745 (PMC2581440; doi:10.1371/journal.pone.0003745)
Supplement: Figure S2 — (8.74 MB PDF) [file pone.0003745.s002.pdf]

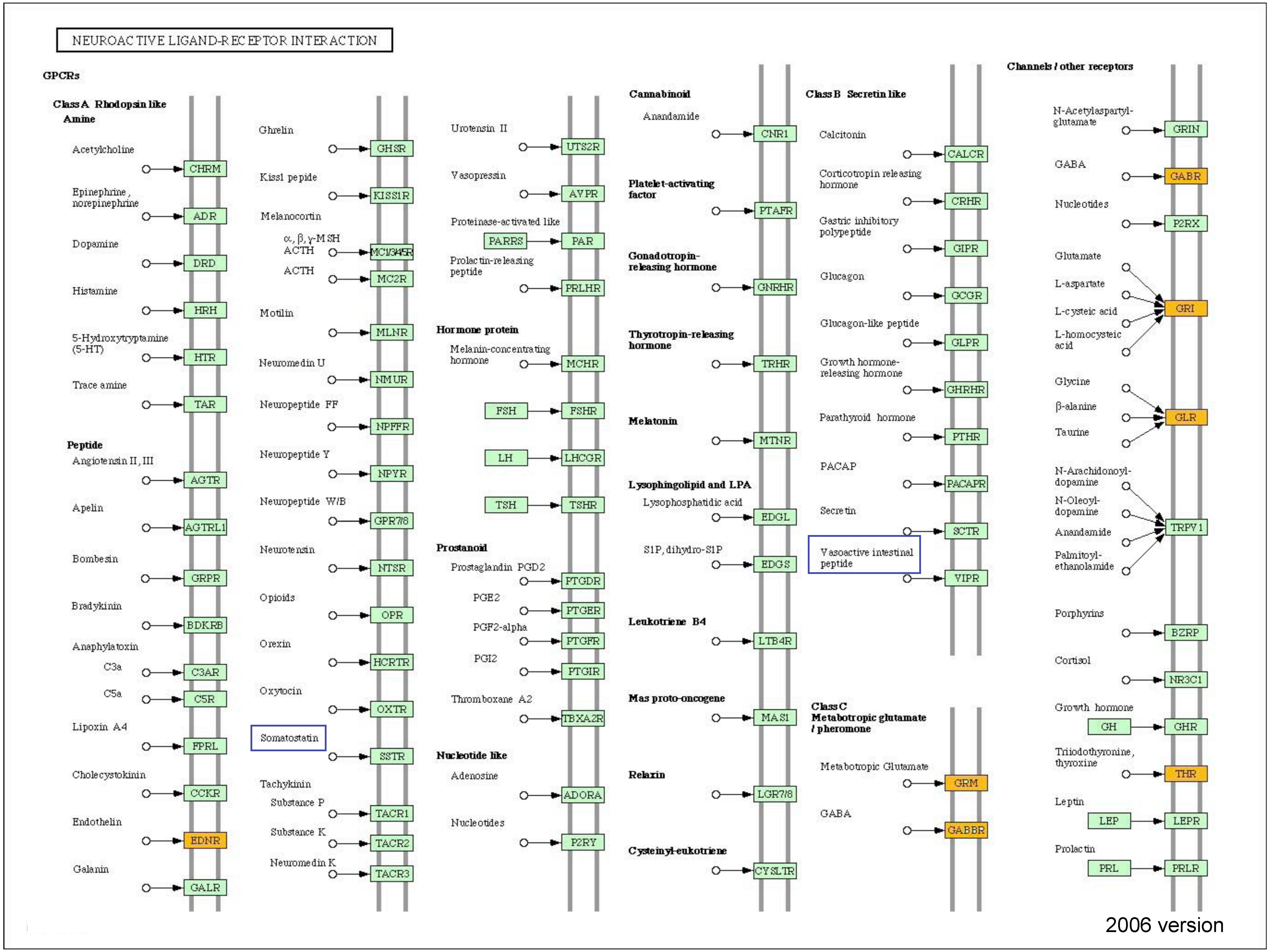

Supplemental Figure S2: Standard KEGG neuroactive ligand receptor interaction pathway (both neural and peripheral tissue) of 13 altered genes in the F3 generation female hippocampus with red/orange indicating an increase in expression and blue a decrease in expression with green being no change in expression and white gene absent from the array. The genes altered in the other tissue are as follows: male amygdala (PMCH, GABRH1, Edg1, GABBR) and male hippocampus (Hrh4, Lepr).
