# Supplementary material for: Transgenerational Epigenetic Programming of the Brain Transcriptome and Anxiety Behavior
Source: PLoS One. 2008 Nov 18;3(11):e3745. doi: 10.1371/journal.pone.0003745 (PMC2581440; doi:10.1371/journal.pone.0003745)
Supplement: Table S5 — Transgenerational Female Hippocampus Regulated Genes (0.26 MB PDF) [file pone.0003745.s007.pdf]

Table S5
Transgenerational Female Hippocampus Regulated Genes

	**Apoptosis**				
Sample	F3-Cont	F3-Vinc	**Vin/Con**		
Gene Symbol	Raw	Raw	**Ratio**	Genbank	Gene Title
Bag3	163	103	**0.63**	AI231792	Bcl2-associated athanogene 3
Cbl27	16	160	**9.99**	AF275151	androgen receptor-related apoptosis-associated protein
Dapk1_predicte	55	149	**2.69**	BI281823	death associated protein kinase 1 (predicted)
Faim2	278	425	**1.53**	AF044201	Fas apoptotic inhibitory molecule 2
Pdcd6ip	457	706	**1.54**	BF389407	programmed cell death 6 interacting protein
RGD1306214_p	19	100	**5.32**	BE107277	similar to TGF-beta induced apotosis protein 2 (predicte
Tegt	358	559	**1.56**	NM_019381	testis enhanced gene transcript (Bax inhibitor-1)

	**Cell Cycle**				
Sample	F3-Cont	F3-Vinc	**Vin/Con**		
Gene Symbol	Raw	Raw	**Ratio**	Genbank	Gene Title
Ccnd1	66	176	**2.67**	BI295861	cyclin D1
Ccnd2	41	546	**13.21**	L09752	cyclin D2
Ccnd3	87	139	**1.60**	NM_012766	cyclin D3
Cdc2l5	65	153	**2.37**	AI170800	cell division cycle 2-like 5 (cholinesterase-related cell di
Cdc2l6_predicte	275	480	**1.75**	BG376309	cell division cycle 2-like 6 (CDK8-like) (predicted)
Cdkn1c	66	105	**1.59**	AI013919	cyclin-dependent kinase inhibitor 1C (P57)
Ddb1	53	245	**4.58**	AJ277077	damage-specific DNA binding protein 1
Dab2	117	207	**1.77**	NM_024159	disabled homolog 2 (Drosophila)
Gas6	156	541	**3.47**	NM_057100	growth arrest specific 6
Numa1	100	168	**1.67**	AI599394	nuclear mitotic apparatus protein 1
RGD1560358_p	75	381	**5.11**	AW534965	similar to cell division cycle and apoptosis regulator 1 (p
Unc5b	54	108	**1.98**	BI303989	Unc-5 homolog B (C. elegans)

	**Cytoskeleton-ECM**				
Sample	F3-Cont	F3-Vinc	**Vin/Con**		
Gene Symbol	Raw	Raw	**Ratio**	Genbank	Gene Title
Actb	1427	3416	**2.39**	NM_031144	actin, beta
Add1	60	176	**2.95**	NM_016990	adducin 1 (alpha)
Add3	46	269	**5.82**	AI407835	adducin 3 (gamma)
App	723	1257	**1.74**	BM986220	amyloid beta (A4) precursor protein
Actr1a_predicte	170	292	**1.71**	BI294346	ARP1 actin-related protein 1 homolog A (yeast) (predict
Actr2	1025	3030	**2.96**	BE107525	ARP2 actin-related protein 2 homolog (yeast)
Cdh13	139	525	**3.78**	BG381748	cadherin 13
Cdh2	147	230	**1.56**	AF097593	cadherin 2
Cdh22	36	526	**14.68**	NM_019161	cadherin 22
Chl1	211	848	**4.02**	BF524215	cell adhesion molecule with homology to L1CAM
Cspg5	33	99	**3.00**	AF292102	chondroitin sulfate proteoglycan 5
Cotl1_predicted	434	283	**0.65**	AI411057	coactosin-like 1 (Dictyostelium) (predicted)
Cntn1	82	127	**1.56**	NM_057118	contactin 1
Coro1c_predicte	23	91	**3.86**	BE120171	coronin, actin binding protein 1C (predicted)
Cttn	25	112	**4.45**	AF054618	cortactin
Dcn	142	230	**1.61**	BM390253	decorin
Dstn	305	483	**1.58**	AI170442	destrin
Dpy19l1_predic	145	315	**2.18**	BI296653	dpy-19-like 1 (C. elegans) (predicted)
Dbn1	978	551	**0.56**	NM_031024	drebrin 1
Dctn3_predicte	614	1050	**1.71**	AA893211	dynactin 3 (predicted)
Dst_predicted	72	162	**2.25**	AI235468	dystonin (predicted)
Dtna_predicted	458	878	**1.92**	BF396607	dystrobrevin alpha (predicted)
Dag1	52	138	**2.65**	AW251326	dystroglycan 1
Eml2	*6*	*115*	***17.88***	*AF335571*	*echinoderm microtubule associated protein like 2*
Eml4_predicted	146	91	**0.63**	BE111290	echinoderm microtubule associated protein like 4 (pred
Enc1	20	100	**4.90**	AA997271	Ectodermal-neural cortex 1
Epn2	40	88	**2.21**	NM_021852	epsin 2
Epb4.1l3	133	202	**1.52**	AB032828	erythrocyte protein band 4.1-like 3
Epb4.1l4a_pred	161	106	**0.66**	BE100811	erythrocyte protein band 4.1-like 4a (predicted)
Fndc3b_predict	22	115	**5.22**	AI176320	fibronectin type III domain containing 3B (predicted)
Gpiap1	108	216	**2.00**	BF546337	GPI-anchored membrane protein 1
Hook3	74	113	**1.53**	AI501458	hook homolog 3 (Drosophila)
Icam5_predicte	134	346	**2.58**	BE102418	intercellular adhesion molecule 5, telencephalin (predic
Kif1b	65	285	**4.42**	AB070355	kinesin family member 1B
Kif3a	29	122	**4.22**	BF397677	kinesin family member 3a
Kif5a	45	85	**1.88**	BF408765	kinesin family member 5A

Kif5c_predicted	21	148	**7.17**	BE104278	kinesin family member 5C (predicted)
KIFC2	541	332	**0.61**	AW433953	kinesin family member C2
Klc1	53	246	**4.65**	AI576961	kinesin light chain 1
Kifap3_predicte	16	187	**11.78**	AI227800	kinesin-associated protein 3 (predicted)
Lgals8	57	90	**1.57**	NM_053862	lectin, galactoside-binding, soluble 8
Lrfn3_predicted	58	89	**1.55**	BE101066	leucine rich repeat and fibronectin type III domain conta
Map1lc3a	739	448	**0.61**	AI177372	microtubule-associated protein 1 light chain 3 alpha
Map1lc3b	276	418	**1.52**	AI233190	microtubule-associated protein 1 light chain 3 beta
Map1b	171	625	**3.67**	BG672052	microtubule-associated protein 1b
Mtap2	19	259	**13.31**	X74211	microtubule-associated protein 2
Mtap7_predicte	347	191	**0.55**	BG374192	Microtubule-associated protein 7 (predicted)
Mapre1	74	157	**2.13**	U75920	microtubule-associated protein, RP/EB family, member
Mtmr4_predicte	77	137	**1.78**	AI178892	myotubularin related protein 4 (predicted)
Mtmr9	109	411	**3.77**	BE111884	myotubularin related protein 9
LOC294446 /// l	85	156	**1.84**	M59859	myristoylated alanine rich protein kinase C substrate ///
Ncam1	275	427	**1.56**	AI409738	Neural cell adhesion molecule 1
Hnt	172	791	**4.60**	NM_017354	neurotrimin
Opcml	551	1095	**1.99**	M88709	opioid binding protein/cell adhesion molecule-like
Pclo	27	80	**3.01**	AF138789	piccolo (presynaptic cytomatrix protein)
Pkp4_predicted	54	153	**2.83**	AA819847	plakophilin 4 (predicted)
Podxl2_predicte	149	97	**0.65**	BI276946	podocalyxin-like 2 (predicted)
Col1a2	42	125	**3.00**	BM388837	procollagen, type I, alpha 2
Col11a2	92	56	**0.61**	BM391350	procollagen, type XI, alpha 2
Pfn2	255	446	**1.75**	AF228737	profilin 2
Pcdh17_predict	47	364	**7.68**	BF558981	protocadherin 17 (predicted)
Pcdh19_predict	76	227	**2.97**	AA997710	protocadherin 19 (predicted)
Pcdh7	38	97	**2.54**	AA956340	Protocadherin 7
Pcdha13	12	76	**6.27**	AW524833	protocadherin alpha 13
**Pcdhga1 /// Pc**	**25**	**174**	**7.00**	**BE097805**	**protocadherin gamma subfamily C, 3 /// protocadhe**
Rdx	50	131	**2.62**	AW527313	radixin
Rsn	54	110	**2.03**	NM_031745	restin (Reed-Steinberg cell-expressed intermediate filar
Sparc	698	458	**0.66**	NM_012656	secreted acidic cysteine rich glycoprotein
Srrm2_predicte	143	908	**6.33**	BF408990	serine/arginine repetitive matrix 2 (predicted)
LOC679221	279	432	**1.55**	AA859614	similar to Microtubule-associated protein RP/EB family
Spnb2	123	342	**2.78**	BF559566	Spectrin beta 2
Spire1_predicte	117	182	**1.55**	BM387505	spire homolog 1 (Drosophila) (predicted)
Stmn4	465	713	**1.53**	AF026530	stathmin-like 4
Smap1l	79	141	**1.79**	BE097445	stromal membrane-associated protein 1-like
Sv2a	355	678	**1.91**	NM_057210	synaptic vesicle glycoprotein 2a
Sv2b	156	364	**2.34**	L10362	synaptic vesicle glycoprotein 2b
Tnr	34	229	**6.80**	NM_013045	tenascin R
Tspan5	42	88	**2.10**	AW251317	tetraspanin 5
Tmsb10	1044	636	**0.61**	NM_021261	thymosin, beta 10
Tmod2	110	327	**2.98**	BF567833	tropomodulin 2
Tpm1	178	280	**1.57**	M34135	tropomyosin 1, alpha
Tpm3	735	472	**0.64**	AI071098	tropomyosin 3, gamma
Vcpip1	56	300	**5.37**	BM383657	valosin containing protein (p97)/p47 complex interactin
Vamp1	20	95	**4.79**	M24104	vesicle-associated membrane protein 1
Vapb	13	83	**6.65**	NM_021847	vesicle-associated membrane protein, associated prote
Zyg11bl	80	207	**2.59**	BF567873	zyg-11 homolog B (C. elegans)-like

Development

Sample	F3-Cont	F3-Vinc	**Vin/Con**		
Gene Symbol	Raw	Raw	**Ratio**	Genbank	Gene Title
Apc	77	144	**1.87**	NM_012499	adenomatosis polyposis coli
Aph1a	53	82	**1.56**	BI275921	anterior pharynx defective 1a homolog (C. elegans)
Apeg1	143	86	**0.60**	NM_012905	aortic preferentially expressed gene 1
Atxn2_predictec	410	656	**1.60**	BE109041	ataxin 2 (predicted)
Atxn2l_predicte	52	88	**1.71**	BE349657	ataxin 2-like (predicted)
Bbx_predicted	42	227	**5.40**	BF392234	bobby sox homolog (Drosophila) (predicted)
Bai3_predicted	48	100	**2.08**	BF409866	Brain-specific angiogenesis inhibitor 3 (predicted)
Cops7a_predict	123	190	**1.55**	BE110772	COP9 (constitutive photomorphogenic) homolog, subur
Cpne8_predicte	18	119	**6.75**	AI059204	copine VIII (predicted)
Cryl1	241	135	**0.56**	BI293393	crystallin, lamda 1
**Crim1_predicte**	**31**	**160**	**5.07**	**BI289620**	**cysteine-rich motor neuron 1 (predicted)**
Cyfip2_predicte	131	242	**1.84**	BE105837	cytoplasmic FMR1 interacting protein 2 (predicted)
Dlgap3	17	99	**5.80**	U67139	discs, large (Drosophila) homolog-associated protein 3
Dlgh1	59	113	**1.93**	NM_012788	discs, large homolog 1 (Drosophila)
Dlgh2	26	81	**3.11**	BI296447	Discs, large homolog 2 (Drosophila)

Dlgh4	26	98	**3.85**	NM_019621	discs, large homolog 4 (Drosophila)
Dlgap4	334	537	**1.61**	U67140	discs, large homolog-associated protein 4 (Drosophila)
Elavl1_predicte	156	1107	**7.12**	BE104552	ELAV (embryonic lethal, abnormal vision, Drosophila)-li
Elavl2	413	1045	**2.53**	BF563441	ELAV (embryonic lethal, abnormal vision, Drosophila)-li
Fbn1	82	48	**0.59**	BM389019	fibrillin 1
Fcmd_predictec	232	473	**2.04**	AI101490	Fukuyama type congenital muscular dystrophy homolo
Grhl1_predictec	130	198	**1.52**	BM383749	Grainyhead-like 1 (Drosophila) (predicted)
Hdh	109	65	**0.60**	BI274329	Huntington disease gene homolog
Ibtk_predicted	135	89	**0.66**	AW525218	inhibitor of Bruton agammaglobulinemia tyrosine kinase
Vof16	7	122	**18.13**	BE107282	ischemia related factor vof-16
Kidins220	51	146	**2.86**	AF313464	kinase D-interacting substance 220
Lrrn6a	965	1821	**1.89**	AI711152	leucine rich repeat neuronal 6A
Mpv17l_predict	32	187	**5.78**	AA849966	Mpv17 transgene, kidney disease mutant-like (predicte
Mbp	383	3170	**8.27**	NM_017026	myelin basic protein
Mog	43	88	**2.08**	BF410306	Myelin oligodendrocyte glycoprotein
Mobp	288	801	**2.78**	D28110	myelin-associated oligodendrocytic basic protein
Nsf	292	873	**2.99**	AF142097	N-ethylmaleimide sensitive fusion protein
Napg	80	174	**2.16**	AA956372	N-ethylmaleimide-sensitive factor attachment protein, g
Nedd4a	524	2373	**4.53**	BI284798	neural precursor cell expressed, developmentally down
Neurod2	39	116	**2.97**	NM_019326	neurogenic differentiation 2
LOC681423 /// l	18	117	**6.48**	BE117361	Nipped-B homolog (Drosophila) /// similar to delangin is
Odz2	122	264	**2.17**	NM_020088	odd Oz/ten-m homolog 2 (Drosophila)
Opa1	45	95	**2.12**	BF407962	Optic atrophy 1 homolog (human)
Pmp22	61	137	**2.23**	AW252810	peripheral myelin protein 22
Prg1	132	505	**3.84**	BF394800	plasticity related gene 1
Peo1_predicted	249	158	**0.64**	AA925995	progressive external ophthalmoplegia 1 homolog (huma
Rb1cc1_predict	196	449	**2.28**	BG671668	RB1-inducible coiled-coil 1 (predicted)
Rqcd1	93	160	**1.73**	AI044879	rcd1 (required for cell differentiation) homolog 1 (S. por
Reln	43	89	**2.06**	NM_080394	reelin
Sema6a_predic	60	93	**1.55**	BM387083	sema domain, transmembrane domain (TM), and cytop
LOC682864	36	147	**4.08**	BE116720	similar to 82-kD FMRP Interacting Protein
LOC499716	47	78	**1.67**	AA818641	similar to DnaJ (Hsp40) homolog, subfamily B, membe
LOC686892	44	188	**4.29**	AI180361	similar to muscleblind-like 1 isoform d
RGD1565589_p	151	273	**1.80**	BM390561	similar to myocardial ischemic preconditioning upregula
LOC682507	46	475	**10.31**	AI706673	similar to Neural Wiskott-Aldrich syndrome protein (N-W
RGD1566269_p	381	208	**0.55**	BE118896	Similar to Neuropilin- and tolloid-like protein 1 (predicte
RGD1310358_p	123	252	**2.06**	BF567255	similar to NNX3 (predicted)
RGD1559723_p	23	113	**4.82**	BE114807	similar to Sspn protein (predicted)
LOC680647	36	86	**2.42**	BG667371	Similar to TAFA2 protein
LOC687031	57	164	**2.90**	BE116590	Similar to transmembrane protein SHREW1
Slit3	171	88	**0.51**	BF386446	Slit homolog 3 (Drosophila)
Spg20	59	100	**1.71**	AI576427	spastic paraplegia 20, spartin (Troyer syndrome) homo
Smndc1	60	100	**1.67**	BI288396	survival motor neuron domain containing 1
Syn2	114	561	**4.92**	NM_019159	synapsin II
Sncb	135	228	**1.69**	NM_080777	synuclein, beta
Sncg	*535*	*1003*	***1.87***	*NM_031688*	*synuclein, gamma*
Tor1aip1	146	363	**2.49**	U19614	torsin A interacting protein 1
Trak2	101	844	**8.35**	BG378620	trafficking protein, kinesin binding 2
Trps1_predictec	62	307	**4.91**	BF398245	trichorhinophalangeal syndrome I (predicted)
Trps1_predictec	121	430	**3.55**	BF550315	trichorhinophalangeal syndrome I (predicted)
Wbscr1	16	142	**8.74**	H31800	Williams-Beuren syndrome chromosome region 1 homo
Waspip	626	377	**0.60**	NM_057192	Wiskott-Aldrich syndrome protein interacting protein
Whsc1l1_predic	94	239	**2.56**	AI234807	Wolf-Hirschhorn syndrome candidate 1-like 1 (predicte
Rbaf600	20	77	**3.85**	BF392966	ZUBR1

Electron Transport

Sample	F3-Cont	F3-Vinc	**Vin/Con**		
Gene Symbol	Raw	Raw	**Ratio**	Genbank	Gene Title
Cyp51	613	1238	**2.02**	NM_012941	cytochrome P450, subfamily 51
Glrx2	76	475	**6.27**	BG671304	Glutaredoxin 2 (thioltransferase)
Iag2	46	145	**3.13**	NM_053946	implantation-associated protein
Uqcrc2	241	418	**1.74**	BF290998	ubiquinol cytochrome c reductase core protein 2

Epigenetics/Gene Modifying Factors

Sample	F3-Cont	F3-Vinc	**Vin/Con**		
Gene Symbol	Raw	Raw	**Ratio**	Genbank	Gene Title
Atrx	13	87	**6.48**	BF397805	alpha thalassemia/mental retardation syndrome X-linke
Rere	96	61	**0.64**	BF412271	Arginine-glutamic acid dipeptide (RE) repeats
Ash1l_predictec	20	143	**7.10**	BG663056	ash1 (absent, small, or homeotic)-like (Drosophila) (pre

Brd2	78	127	**1.64**	AA946361	bromodomain containing 2
Brd3_predicted	62	271	**4.37**	AI172375	bromodomain containing 3 (predicted)
Brd4	12	178	**14.61**	BM389207	bromodomain containing 4
Csnk2a1	19	268	**13.81**	BF288177	casein kinase II, alpha 1 polypeptide
Csnk2a2_predic	38	106	**2.81**	BI290750	casein kinase II, alpha 2, polypeptide (predicted)
Chd1_predicted	60	137	**2.28**	AA955721	chromodomain helicase DNA binding protein 1 (predicte
Chd2_predicted	*91*	*51*	***0.56***	*BF396633*	*Chromodomain helicase DNA binding protein 2 (predic*
Cxxc5	276	564	**2.04**	BI281838	CXXC finger 5
Tia1	46	99	**2.14**	BI298817	Cytotoxic granule-associated RNA binding protein 1
Epc2_predicted	64	138	**2.17**	AW918173	enhancer of polycomb homolog 2 (Drosophila) (predicte
H2afy	169	403	**2.38**	M99065	H2A histone family, member Y
Hdac4_predicte	46	120	**2.63**	BF419085	histone deacetylase 4 (predicted)
Impact	155	253	**1.63**	BG664101	imprinted and ancient
Id4	23	99	**4.38**	AI412150	inhibitor of DNA binding 4
Jmjd3_predicte	50	150	**3.02**	BE118720	jumonji domain containing 3 (predicted)
Metap1_predict	58	91	**1.59**	AI101470	methionyl aminopeptidase 1 (predicted)
Prdm2	33	168	**5.05**	BF388420	PR domain containing 2, with ZNF domain
Pcmtd2_predict	709	1292	**1.82**	AA955163	protein-L-isoaspartate (D-aspartate) O-methyltransfera
RGD1566399_p	95	155	**1.63**	BE098769	similar to MYST histone acetyltransferase monocytic le
LOC314964	24	83	**3.51**	AA965250	similar to PHD finger protein 20-like 1 isoform 1
LOC681178 /// L	134	234	**1.75**	BI288196	similar to polycomb group ring finger 5
Smarca2	70	148	**2.12**	BE102268	SWI/SNF related, matrix associated, actin dependent re
Smarca4	24	223	**9.41**	BE111847	SWI/SNF related, matrix associated, actin dependent re

Golgi Apparatus

Sample	F3-Cont	F3-Vinc	**Vin/Con**		
Gene Symbol	Raw	Raw	**Ratio**	Genbank	Gene Title
Ap1s1_predicte	215	524	**2.44**	AA955775	adaptor protein complex AP-1, sigma 1 (predicted)
Ap2b1	26	95	**3.66**	M34176	adaptor-related protein complex 2, beta 1 subunit
Copz1_predicte	124	217	**1.75**	BF406339	coatomer protein complex, subunit zeta 1 (predicted)
Cbfa2t3_predict	45	93	**2.07**	BE115481	core-binding factor, runt domain, alpha subunit 2; transl
Gga3_predicted	320	114	**0.35**	BE112983	golgi associated, gamma adaptin ear containing, ARF b
Gla	60	112	**1.86**	BM385090	galactosidase, alpha
Golph3	58	101	**1.74**	NM_023977	golgi phosphoprotein 3
Hs2st1	226	439	**1.94**	AI714262	heparan sulfate 2-O-sulfotransferase 1
Pde4dip	54	114	**2.11**	NM_022382	phosphodiesterase 4D interacting protein (myomegalin
Pde4dip	450	278	**0.62**	AI763912	Phosphodiesterase 4D interacting protein (myomegalin
RGD1560511_p	*79*	*189*	***2.40***	*BI281965*	*similar to Vps41 protein (predicted)*
Surf4	146	97	**0.66**	AI227937	surfeit 4
Tgoln2	53	98	**1.87**	AW917192	trans-golgi network protein 2

Growth Factors, Cyto- and Chemokines

Sample	F3-Cont	F3-Vinc	**Vin/Con**		
Gene Symbol	Raw	Raw	**Ratio**	Genbank	Gene Title
Api5_predicted	64	108	**1.68**	H34636	apoptosis inhibitor 5 (predicted)
Bdnf	137	236	**1.72**	NM_012513	brain derived neurotrophic factor
Cxcl12	52	155	**2.97**	AF189724	chemokine (C-X-C motif) ligand 12
Crlf1_predicted	44	81	**1.84**	AA866388	cytokine receptor-like factor 1 (predicted)
Igf2	131	308	**2.36**	NM_031511	insulin-like growth factor 2
Il6st	153	382	**2.49**	BM383427	interleukin 6 signal transducer
Gdf1_predicted	716	433	**0.60**	AI549010	longevity assurance homolog 1 (S. cerevisiae) (predicte
Negr1	155	964	**6.21**	NM_021682	neuronal growth regulator 1
Ogn_predicted	55	117	**2.14**	BG664221	osteoglycin (predicted)
Sep3	88	183	**2.08**	NM_019375	septin 3
Sept6_predicted	117	262	**2.24**	AW532098	Septin 6 (predicted)
Sep7	294	569	**1.93**	NM_022616	septin 7
Sst	468	307	**0.66**	NM_012659	somatostatin
Tgfb2	***121***	***70***	***0.58***	***BE117736***	***Transforming growth factor, beta 2***
Tmeff1	74	177	**2.38**	NM_023020	transmembrane protein with EGF-like and two follistatin

Immune Response

Sample	F3-Cont	F3-Vinc	**Vin/Con**		
Gene Symbol	Raw	Raw	**Ratio**	Genbank	Gene Title
Cd2bp2_predict	32	76	**2.38**	BG379822	CD2 antigen (cytoplasmic tail) binding protein 2 (predic
Cd74	70	203	**2.89**	NM_013069	CD74 antigen (invariant polypeptide of major histocomp
Cd99	64	101	**1.59**	AI235284	CD99 antigen
C7 /// Tubb2c	51	89	**1.76**	BF284922	complement component 7 /// tubulin, beta 2c
Igsf4b_predicte	36	109	**3.06**	BF409960	immunoglobulin superfamily, member 4B (predicted)
Ke2	530	938	**1.77**	AW434268	MHC class II region expressed gene KE2

Pafah1b1	45	141	**3.11**	BG663460	platelet-activating factor acetylhydrolase, isoform Ib, al
RT1-Ke4	*301*	*134*	***0.44***	*BM389027*	*RT1 class I, locus Ke4*
RT1-Aw2	208	34	**0.16**	AA801218	RT1 class Ib, locus Aw2
RT1-S3	*86*	*51*	***0.59***	*AJ243974*	*RT1 class Ib, locus S3*
RT1-Da	45	153	**3.36**	Y00480	RT1 class II, locus Da
RT1-149	109	65	**0.60**	BI282965	RT1-149 protein
Sema4f	143	95	**0.66**	NM_019272	sema domain, immunoglobulin domain (Ig), transmemb
LOC690085	38	199	**5.29**	AW534002	Similar to B-cell CLL/lymphoma 7A
LOC680404 /// l	117	1507	**12.89**	BG374818	similar to Complement C1q-like protein 3 precursor (Gli
LOC296637	118	259	**2.20**	AA944347	similar to HLA-B associated transcript-2 isoform a
RGD1563429_p	61	463	**7.56**	BM391860	similar to T-cell activation leucine repeat-rich protein (p
Stag1_predicte	*100*	*49*	***0.49***	*AI071210*	*Stromal antigen 1 (predicted)*
Thy1	213	597	**2.81**	NM_012673	thymus cell antigen 1, theta
Wrb	87	147	**1.69**	BI280216	tryptophan rich basic protein

Metabolism & Transport

Sample	F3-Cont	F3-Vinc	**Vin/Con**		
Gene Symbol	Raw	Raw	**Ratio**	Genbank	Gene Title
Agpat1	74	167	**2.24**	BF390804	1-acylglycerol-3-phosphate O-acyltransferase 1
Abhd8_predicte	10	227	**22.52**	AI137533	abhydrolase domain containing 8 (predicted)
Acsl1	52	126	**2.45**	BI277523	acyl-CoA synthetase long-chain family member 1
Adpgk	65	170	**2.60**	BE102146	ADP-dependent glucokinase
Aldh1a2	59	119	**2.01**	NM_053896	aldehyde dehydrogenase family 1, subfamily A2
Aldh6a1	50	103	**2.05**	NM_031057	aldehyde dehydrogenase family 6, subfamily A1
Accn2	95	58	**0.61**	AJ309926	amiloride-sensitive cation channel 2, neuronal
Aqp4	*84*	*255*	***3.03***	*NM_012825*	*aquaporin 4*
Aqp9	86	53	**0.61**	NM_022960	aquaporin 9
Arcn1	*55*	*153*	***2.78***	*BF414061*	*archain 1*
**Arg2**	**125**	**245**	**1.96**	**AI574994**	**Arginase 2**
Asna1	194	308	**1.59**	AI406558	arsA arsenite transporter, ATP-binding, homolog 1 (bac
Arsb	72	114	**1.57**	AW917073	arylsulfatase B
Atp2a2	79	331	**4.19**	J04024	ATPase, Ca++ transporting, cardiac muscle, slow twitc
Atp2b1	1829	3444	**1.88**	NM_053311	ATPase, Ca++ transporting, plasma membrane 1
Atp6v1a1_predi	636	1617	**2.54**	BI289589	ATPase, H transporting, lysosomal V1 subunit A (predic
Atp6v1b2	568	1031	**1.82**	NM_057213	ATPase, H transporting, lysosomal V1 subunit B2
Atp6v0a2	37	112	**3.03**	BE110564	ATPase, H+ transporting, lysosomal V0 subunit a isofo
Atp1a1	45	293	**6.56**	M74494	ATPase, Na+/K+ transporting, alpha 1 polypeptide
Atp1a3	1132	746	**0.66**	NM_012506	ATPase, Na+/K+ transporting, alpha 3 polypeptide
Atp1b2	15	194	**12.92**	U45946	ATPase, Na+/K+ transporting, beta 2 polypeptide
B3gat1	43	87	**2.03**	NM_054003	beta-1,3-glucuronyltransferase 1 (glucuronosyltransfer
Bace1	38	149	**3.88**	BG377970	Beta-site APP cleaving enzyme 1
Cacna2d3	20	99	**4.94**	BE108017	Calcium channel, voltage-dependent, alpha 2/delta 3 s
Cacnb1	79	144	**1.81**	NM_017346	calcium channel, voltage-dependent, beta 1 subunit
Cacng3	117	218	**1.87**	AF361340	calcium channel, voltage-dependent, gamma subunit 3
Cacng8	95	60	**0.63**	BE114019	Calcium channel, voltage-dependent, gamma subunit 8
Clstn2	24	268	**11.10**	BE109141	Calsyntenin 2
Chst2_predicte	290	585	**2.02**	AW520944	carbohydrate sulfotransferase 2 (predicted)
Car11	128	229	**1.78**	AW252112	carbonic anhydrase 11
Ca3	*28*	*228*	***8.01***	*AB030829*	*carbonic anhydrase 3*
Cs	136	269	**1.98**	NM_130755	citrate synthase
Cplx1	56	184	**3.29**	U35098	complexin 1
Cplx2	47	345	**7.29**	NM_053878	complexin 2
Ckmt1	1166	750	**0.64**	BI301453	creatine kinase, mitochondrial 1, ubiquitous
Capon	94	339	**3.60**	AF037071	C-terminal PDZ domain ligand of neuronal nitric oxide s
Dgkg	160	599	**3.75**	NM_013126	diacylglycerol kinase, gamma
Elovl6	54	271	**5.03**	BF396857	ELOVL family member 6, elongation of long chain fatty
Etnk1_predicte	83	133	**1.61**	BG668816	ethanolamine kinase 1 (predicted)
Xpo4_predicted	25	108	**4.31**	BF397646	exportin 4 (predicted)
Fmo1	58	87	**1.51**	NM_012792	flavin containing monooxygenase 1
Gja1	13	81	**6.11**	BG378227	gap junction membrane channel protein alpha 1
Got1	438	822	**1.87**	D00252	glutamate oxaloacetate transaminase 1
Glul	270	509	**1.88**	BI296610	glutamate-ammonia ligase (glutamine synthase)
Gls	67	145	**2.15**	M22586	glutaminase
Gapdh	1258	3188	**2.53**	X_Rat_GAPD	glyceraldehyde-3-phosphate dehydrogenase
Gpd1	112	66	**0.59**	BF399697	Glycerol-3-phosphate dehydrogenase 1 (soluble)
Gsk3b	16	115	**7.03**	BF287444	glycogen synthase kinase 3 beta
Gda	830	1718	**2.07**	AF245172	guanine deaminase
Hba-a1 /// LOC	4846	2874	**0.59**	AI179404	hemoglobin alpha, adult chain 1 /// hemoglobin alpha 2
Hk1	93	222	**2.40**	_Rat_Hexokin	hexokinase 1

Hcn2	41	110	**2.67**	AW532988	hyperpolarization activated cyclic nucleotide-gated pota
Hprt	43	78	**1.81**	BE117366	Hypoxanthine guanine phosphoribosyl transferase
Kpnb1	18	92	**5.01**	NM_017063	karyopherin (importin) beta 1
Kcnip2	17	93	**5.40**	AF269283	Kv channel-interacting protein 2
Lypla2	63	132	**2.11**	NM_031342	lysophospholipase 2
Man1a_predicte	399	237	**0.59**	AA892549	mannosidase 1, alpha (predicted)
Man2a2_predic	295	192	**0.65**	BE099244	Mannosidase 2, alpha 2 (predicted)
Magi2	36	168	**4.69**	AF130819	membrane associated guanylate kinase, WW and PDZ
Maoa	235	752	**3.20**	D00688	monoamine oxidase A
Mgll	15	216	**14.29**	AY081195	monoglyceride lipase
Asah1	80	123	**1.54**	NM_053407	N-acylsphingosine amidohydrolase 1
Por	26	133	**5.12**	AI407454	P450 (cytochrome) oxidoreductase
Pex2	54	114	**2.13**	BF413248	Peroxin 2
Ppap2b	30	571	**18.84**	AW253995	phosphatidic acid phosphatase type 2B
Ptdss1	349	611	**1.75**	AA851302	phosphatidylserine synthase 1
Plcl1	1062	696	**0.66**	NM_053456	phospholipase C-like 1
Prps1	183	292	**1.59**	M29392	phosphoribosyl pyrophosphate synthetase 1
Prps2	124	203	**1.64**	NM_012634	phosphoribosyl pyrophosphate synthetase 2
Kctd1	212	140	**0.66**	AI501338	Potassium channel tetramerisation domain containing 1
Kctd12_predicte	105	162	**1.54**	AA800908	Potassium channel tetramerisation domain containing 1
Kcnj3	37	86	**2.35**	U09243	potassium inwardly-rectifying channel, subfamily J, mei
Kcnd2	326	900	**2.76**	AW528891	potassium voltage gated channel, Shal-related family, r
Kcnd3	177	274	**1.55**	NM_031739	potassium voltage gated channel, Shal-related family, r
Kcna1	129	511	**3.97**	M26161	potassium voltage-gated channel, shaker-related subfa
Kcng2	56	89	**1.58**	AI407741	potassium voltage-gated channel, subfamily G, membe
Kcnq3	43	108	**2.48**	BF400850	Potassium voltage-gated channel, subfamily Q, membe
Psap	523	937	**1.79**	NM_013013	prosaposin
Pin1_predicted	539	892	**1.65**	BM388765	protein (peptidyl-prolyl cis/trans isomerase) NIMA-intera
Ahcyl1_predicte	199	486	**2.45**	BF397074	S-adenosylhomocysteine hydrolase-like 1 (predicted)
Srpk2_predicted	24	262	**10.93**	BE113419	serine/arginine-rich protein specific kinase 2 (predicted)
LOC365090	45	109	**2.44**	AW140661	similar to 5-nucleotidase, cytosolic II
LOC500282	173	306	**1.77**	BG671292	similar to ADP-ribosylation factor-like 10C
LOC682926 /// l	*91*	*136*	***1.50***	*BF557618*	*similar to chromatin modifying protein 1B*
RGD1311444_p	427	768	**1.80**	BF557088	similar to intracellular membrane-associated calcium-in
LOC679692 /// l	43	168	**3.89**	BE115518	similar to lysophosphatidylglycerol acyltransferase 1
LOC498358	48	128	**2.67**	AW918899	similar to solute carrier family 30 (zinc transporter), mei
LOC499587	276	1370	**4.96**	AW529244	similar to solute carrier family 7, member 14
RGD1564893_p	151	100	**0.66**	AI410604	similar to surfeit 5 isoform b (predicted)
Scn3a	37	158	**4.33**	NM_013119	sodium channel, voltage-gated, type III, alpha polypept
Slc1a3	129	477	**3.70**	AF265360	solute carrier family 1 (glial high affinity glutamate trans
Slc16a7	65	134	**2.05**	X97445	solute carrier family 16 (monocarboxylic acid transporte
Slc17a7	85	271	**3.19**	NM_053859	solute carrier family 17 (sodium-dependent inorganic pl
Slc2a13	68	279	**4.08**	NM_133611	solute carrier family 2 (facilitated glucose transporter), r
Slc24a2	233	1304	**5.60**	AF021923	solute carrier family 24 (sodium/potassium/calcium exc
Slc25a12_predi	200	124	**0.62**	AI578095	Solute carrier family 25 (mitochondrial carrier, Aralar), n
Slc27a4	161	97	**0.60**	BI286134	solute carrier family 27 (fatty acid transporter), member
Slc3a1	277	983	**3.55**	NM_017216	solute carrier family 3, member 1
Slc35a1_predic	56	88	**1.58**	BE103444	solute carrier family 35 (CMP-sialic acid transporter), m
Slc4a4	8	143	**19.07**	AF210250	solute carrier family 4, member 4
Slc5a3	765	455	**0.60**	BE116021	solute carrier family 5 (inositol transporters), member 3
Slco3a1	51	109	**2.15**	AF239219	solute carrier organic anion transporter family, member
St3gal4	34	87	**2.58**	BF289499	ST3 beta-galactoside alpha-2,3-sialyltransferase 4
Scd2	*98*	*559*	***5.73***	*BE107760*	*stearoyl-Coenzyme A desaturase 2*
Sc5d	151	290	**1.92**	AB052846	sterol-C5-desaturase (fungal ERG3, delta-5-desaturase
Snap91	173	95	**0.55**	AW531009	Synaptosomal-associated protein, 91kDa homolog (mo
Syt1	50	260	**5.22**	AI413003	synaptotagmin I
Syt4	27	312	**11.76**	L38247	synaptotagmin IV
Syt11	21	89	**4.15**	NM_031667	synaptotagmin XI
Txnrd1	53	86	**1.64**	U63923	thioredoxin reductase 1
---	*77*	*221*	***2.87***	*AI029275*	*Transcribed locus, weakly similar to XP_001054001.1*
Ugt8	82	154	**1.88**	L21698	UDP galactosyltransferase 8
Galnt1	41	104	**2.53**	NM_024373	UDP-N-acetyl-alpha-D-galactosamine:polypeptide N-ac
Galnt2_predicte	156	282	**1.81**	BE111638	UDP-N-acetyl-alpha-D-galactosamine:polypeptide N-ac
Vip	250	159	**0.64**	AI412212	vasoactive intestinal polypeptide
Vdac1	59	283	**4.79**	AF268467	voltage-dependent anion channel 1

	**Proteolysis**				
Sample	F3-Cont	F3-Vinc	**Vin/Con**		
Gene Symbol	Raw	Raw	**Ratio**	Genbank	Gene Title

Cpd	174	846	**4.86**	NM_012836	carboxypeptidase D
Dcun1d4_predi	43	132	**3.05**	BF288456	DCN1, defective in cullin neddylation 1, domain contain
Dpp6	80	212	**2.66**	NM_022850	dipeptidylpeptidase 6
Dpp8_predicted	54	111	**2.06**	AI175505	dipeptidylpeptidase 8 (predicted)
Laptm4a	500	1185	**2.37**	AW525776	lysosomal-associated protein transmembrane 4A
LOC290864	56	93	**1.66**	AI408662	similar to Ubiquitin carboxyl-terminal hydrolase 4 (Ubiq
LOC300472	48	78	**1.63**	BE097616	similar to Vacuolar protein sorting 26 homolog (VPS26
LOC316395	63	191	**3.02**	BE119116	similar to HECT, C2 and WW domain containing E3 ubi
LOC679539 /// l	230	533	**2.31**	AW142658	ubiquitin-conjugating enzyme E2 variant 1 (predicted) /
**LOC686268 /// l**	**138**	**287**	**2.07**	**BE105606**	**similar to SUMO/sentrin specific protease 5 (predic**
Mmp14	22	81	**3.62**	X83537	matrix metallopeptidase 14 (membrane-inserted)
Mmp16	64	104	**1.64**	NM_080776	matrix metalloproteinase 16
Pja2	334	994	**2.98**	AI600019	praja 2, RING-H2 motif containing
Prss22_predicte	55	109	**1.97**	BM389391	protease, serine, 22 (predicted)
RGD1560364_p	147	77	**0.53**	BM384685	similar to vacuolar protein sorting 13C protein (predicte
RGD1561386_p	28	93	**3.31**	BE116890	similar to CBL E3 ubiquitin protein ligase (Signal transd
RGD1563278_p	18	148	**8.06**	BF402371	similar to Ubiquitin ligase protein DZIP3 (DAZ-interactin
RGD1565984_p	86	57	**0.66**	BE097603	similar to Ubiquitin carboxyl-terminal hydrolase 35 (Ubi
**Rnf187_predic**	**32**	**93**	**2.88**	**AI502527**	**ring finger protein 187 (predicted)**
Rnf6_predicte	***22***	***117***	***5.25***	***BI296352***	***ring finger protein (C3H2C3 type) 6 (predicted)***
Saps1_predicte	116	184	**1.59**	BF399333	SAPS domain family, member 1 (predicted)
Senp6_predicte	96	147	**1.54**	H32530	SUMO/sentrin specific peptidase 6 (predicted)
Siah1a	76	116	**1.53**	NM_080905	seven in absentia 1A
Smurf2_predicte	9	106	**12.01**	BF393807	SMAD specific E3 ubiquitin protein ligase 2 (predicted)
Timp2	31	111	**3.54**	BF523128	tissue inhibitor of metalloproteinase 2
Tpp1	191	480	**2.51**	NM_031357	tripeptidyl peptidase I
Ube2d2	128	391	**3.05**	BF283846	Ubiquitin-conjugating enzyme E2D 2
Ube2g1	113	240	**2.12**	AF099093	ubiquitin-conjugating enzyme E2G 1 (UBC7 homolog, (
Ube2n	723	456	**0.63**	BI294702	ubiquitin-conjugating enzyme E2N
Ubl3	128	217	**1.69**	BG672206	ubiquitin-like 3
Usp1	16	110	**6.90**	AW525342	ubiquitin specific peptdiase 1
Usp32_predicte	20	84	**4.28**	AI231505	ubiquitin specific protease 32 (predicted)
Usp33	156	333	**2.13**	BE108244	Ubiquitin specific peptidase 33
Usp47_predicte	*119*	*197*	***1.66***	*AI407830*	*ubiquitin specific protease 47 (predicted)*
Usp7	156	381	**2.45**	AI175593	Ubiquitin specific protease 7 (herpes virus-associated)
Usp8_predicted	224	361	**1.61**	AI137190	ubiquitin specific protease 8 (predicted)
Wsb2	106	211	**2.00**	AA848210	WD repeat and SOCS box-containing 2

Receptors & Binding Proteins

Sample	F3-Cont	F3-Vinc	**Vin/Con**		
Gene Symbol	Raw	Raw	**Ratio**	Genbank	Gene Title
Atrn	455	301	**0.66**	AB038388	attractin
Abca1	*35*	*124*	***3.58***	*AI502114*	*ATP-binding cassette, sub-family A (ABC1), member 1*
Aebp1_predicte	64	97	**1.51**	BI278482	AE binding protein 1 (predicted)
Agtrap	158	103	**0.65**	BE349785	angiotensin II, type I receptor-associated protein
Apba2	317	513	**1.62**	NM_031780	amyloid beta (A4) precursor protein-binding, family A, m
Appbp2	43	122	**2.84**	BE113611	amyloid beta precursor protein (cytoplasmic tail) bindin
Bmpr1a	70	124	**1.77**	NM_030849	bone morphogenetic protein receptor, type 1A
Bmpr2	8	176	**22.08**	BE118651	bone morphogenic protein receptor, type II (serine/thre
Chp /// RGD156	58	129	**2.23**	AB070350	calcium binding protein p22 /// similar to calcium bindin
Crebl2	*56*	*163*	***2.91***	*BE102391*	*cAMP responsive element binding protein-like 2*
Ednrb	21	230	**11.00**	X57764	endothelin receptor type B
Egfr	8	87	**11.17**	M37394	epidermal growth factor receptor
Epha7	72	114	**1.58**	U21955	Eph receptor A7
Esrrg	47	97	**2.06**	AA996810	Estrogen-related receptor gamma
Fgfr1	103	233	**2.26**	S54008	Fibroblast growth factor receptor 1
Gabbr1	7	161	**22.23**	Y10369	gamma-aminobutyric acid (GABA) B receptor 1
Gabrb3	25	117	**4.69**	NM_017065	gamma-aminobutyric acid (GABA-A) receptor, subunit b
Gabrg1	48	103	**2.13**	NM_080586	gamma-aminobutyric acid A receptor, gamma 1
Gpr21_predicte	622	368	**0.59**	BG374688	G protein-coupled receptor 21 (predicted)
Gpr37l1	356	632	**1.78**	AF087947	G protein-coupled receptor 37-like 1
Gpr85	232	367	**1.58**	AF203907	G protein-coupled receptor 85
Gpr89_predicte	128	213	**1.67**	BI275972	G protein-coupled receptor 89 (predicted)
Glrb	128	195	**1.53**	NM_053296	glycine receptor, beta subunit
Grm3	247	725	**2.93**	AW522430	glutamate receptor, metabotropic 3
Grm5	59	572	**9.76**	AW526330	Glutamate receptor, metabotropic 5
Htr4	253	148	**0.58**	BF401317	5-hydroxytryptamine (serotonin) receptor 4
**Igfbp2**	**156**	**266**	**1.71**	**NM_013122**	**insulin-like growth factor binding protein 2**
Itm2b	15	100	**6.75**	AW534352	Integral membrane protein 2B

LOC498912	170	288	**1.69**	AA859108	similar to Alpha- and gamma-adaptin-binding protein p3
LOC681927 /// l	66	109	**1.65**	BF419095	similar to SEC24 related gene family, member C
LOC688018	74	506	**6.82**	BM389412	similar to SH3-domain binding protein 3
LOC689560 /// l	9	80	**9.30**	BF418135	ryanodine receptor 2, cardiac /// ryanodine receptor 2 ((
Lphn1	181	501	**2.76**	AF111099	latrophilin 1
Lrp6_predicted	18	125	**6.86**	BM391816	low density lipoprotein receptor-related protein 6 (predi
Nab1	51	78	**1.53**	NM_022856	Ngfi-A binding protein 1
Narg1_predicte	20	249	**12.33**	AW527638	NMDA receptor-regulated gene 1 (predicted)
Ncald	85	239	**2.82**	BF420172	neurocalcin delta
Nisch	182	342	**1.88**	BE108587	nischarin
Nrp2	593	364	**0.61**	AA859669	Neuropilin 2
Nrxn1	230	993	**4.32**	NM_021767	neurexin 1
Nrxn3	271	170	**0.63**	NM_053817	neurexin 3
Oprl1	123	81	**0.66**	NM_031569	opioid receptor-like 1
Osbp2_predicte	140	213	**1.53**	AI112698	oxysterol binding protein 2 (predicted)
Osbpl1a	35	121	**3.41**	AI137224	oxysterol binding protein-like 1A
Otub1_predicte	155	331	**2.13**	BI274378	OTU domain, ubiquitin aldehyde binding 1 (predicted)
P34	41	102	**2.47**	NM_134398	p34 protein
Pgrmc2	84	179	**2.14**	AI137488	progesterone receptor membrane component 2
Plxdc2_predicte	34	242	**7.14**	BE116194	plexin domain containing 2 (predicted)
Plxnb1_predicte	58	95	**1.64**	BI294858	plexin B1 (predicted)
RGD1308847_p	37	81	**2.19**	AI716277	similar to SPla/RYanodine receptor SPRY (1J970) (pre
RGD1559552_p	24	243	**10.01**	BF396678	similar to peroxisome proliferator-activated receptor bin
RGD1560070_p	92	901	**9.75**	BG380409	similar to ataxin 2-binding protein 1 isoform 2 (predicted
RGD1560587_p	238	590	**2.48**	AW525048	similar to Eph receptor A4 (predicted)
Rimbp2	66	170	**2.59**	BE102826	RIM binding protein 2
Rora_predicted	153	230	**1.50**	AI235414	RAR-related orphan receptor alpha (predicted)
Rtn4r	46	101	**2.20**	NM_053613	reticulon 4 receptor
Sec14l2	32	95	**2.96**	NM_053801	SEC14-like 2 (S. cerevisiae)
Sec61a2_predic	840	532	**0.63**	BG381529	Sec61, alpha subunit 2 (S. cerevisiae) (predicted)
Sh3bgrl_predict	261	411	**1.57**	BG672648	SH3-binding domain glutamic acid-rich protein like (pre
**Stxbp1**	**65**	**162**	**2.48**	**U06069**	**syntaxin binding protein 1**
Tbl1xr1_predict	27	112	**4.12**	AA944938	transducin (beta)-like 1X-linked receptor 1 (predicted)
Thra	15	97	**6.44**	M18028	thyroid hormone receptor alpha
Thrap2_predicte	132	436	**3.31**	BE109224	thyroid hormone receptor associated protein 2 (predicte
Wbp4	32	146	**4.58**	BG663444	WW domain binding protein 4

	**Signaling**				
Sample	F3-Cont	F3-Vinc	**Vin/Con**		
Gene Symbol	Raw	Raw	**Ratio**	Genbank	Gene Title
Abi1	311	1190	**3.83**	NM_024397	abl-interactor 1
Adnp	37	83	**2.25**	NM_022681	activity-dependent neuroprotective protein
Adrbk1	65	156	**2.40**	NM_012776	adrenergic receptor kinase, beta 1
**Agtpbp1_predi**	**48**	**103**	**2.13**	**BF553179**	**ATP/GTP binding protein 1 (predicted)**
Ak1	47	82	**1.75**	NM_024349	adenylate kinase 1
Akap13	72	178	**2.45**	AW526712	A kinase (PRKA) anchor protein 13
Anp32a	80	164	**2.05**	NM_012903	acidic (leucine-rich) nuclear phosphoprotein 32 family, r
Anp32e	98	149	**1.53**	AI008642	acidic (leucine-rich) nuclear phosphoprotein 32 family, r
Apln	44	121	**2.73**	NM_031612	apelin, AGTRL1 ligand
Arhgap1_predic	24	80	**3.37**	AI535143	Rho GTPase activating protein 1 (predicted)
Arhgap5	23	162	**7.07**	BM384008	Rho GTPase activating protein 5
Ascc3l1	81	143	**1.77**	AI502051	Activating signal cointegrator 1 complex subunit 3-like 1
Brinp2	89	154	**1.73**	BF551863	BMP/retinoic acid-inducible neural-specific protein 2
**Camk2a**	**565**	**867**	**1.53**	**BM384558**	**calcium/calmodulin-dependent protein kinase II, alp**
Camk2b	1041	2482	**2.38**	NM_021739	calcium/calmodulin-dependent protein kinase II, beta
Camk2g	165	930	**5.62**	NM_133605	calcium/calmodulin-dependent protein kinase II gamma
Camk2n1	191	400	**2.09**	BE111167	Calcium/calmodulin-dependent protein kinase II inhibito
Canx	93	167	**1.79**	BI297860	calnexin
Cap1	172	434	**2.53**	NM_022383	CAP, adenylate cyclase-associated protein 1 (yeast)
Cap2	272	806	**2.96**	NM_053874	CAP, adenylate cyclase-associated protein, 2 (yeast)
Cd47	247	385	**1.56**	NM_019195	CD47 antigen (Rh-related antigen, integrin-associated s
Cdc42bpb	23	78	**3.35**	AF021936	Cdc42 binding protein kinase beta
Centg2_predict	275	650	**2.37**	BF558819	centaurin, gamma 2 (predicted)
Centg3_predict	4	88	**24.82**	AW252124	centaurin, gamma 3 (predicted)
**Cit**	**98**	**269**	**2.75**	**AA957183**	**Citron**
Cnksr2	60	124	**2.06**	AF102853	connector enhancer of kinase suppressor of Ras 2
Csda	162	87	**0.54**	AA866458	cold shock domain protein A
Csnk1a1	158	253	**1.60**	U77583	casein kinase 1, alpha 1
Csnk1d	83	141	**1.71**	L07578	casein kinase 1, delta

Ctnnb1	70	162	**2.33**	NM_053357	catenin (cadherin associated protein), beta 1
Ehd4	62	103	**1.67**	BF395171	EH-domain containing 4
Ensa	117	311	**2.65**	BI289922	endosulfine alpha
Epha5	27	175	**6.49**	BF416432	EphA5
Farp1_predicted	16	118	**7.16**	AI547942	FERM, RhoGEF (Arhgef) and pleckstrin domain protein
Freq	25	77	**3.11**	NM_024366	frequenin homolog (Drosophila)
Gapvd1_predict	161	247	**1.54**	BE102596	GTPase activating protein and VPS9 domains 1 (predic
Garnl1	92	140	**1.52**	AF041107	GTPase activating RANGAP domain-like 1
Gnao	189	726	**3.85**	AF413212	guanine nucleotide binding protein, alpha o
Gnb1	1029	526	**0.51**	NM_030987	guanine nucleotide binding protein, beta 1
Gnb2	384	657	**1.71**	AF277892	guanine nucleotide binding protein, beta polypeptide 2
Gria2	568	1541	**2.72**	M85035	glutamate receptor, ionotropic, AMPA2
Gria3	17	84	**4.93**	M85036	glutamate receptor, ionotropic, AMPA3 (alpha 3)
Grinl1a	104	197	**1.90**	BG664035	glutamate receptor, ionotropic, N-methyl D-aspartate-lik
Grip1	77	51	**0.67**	AI408747	Glutamate receptor interacting protein 1
Hipk2_predicted	73	123	**1.69**	BM392321	homeodomain interacting protein kinase 2 (predicted)
Hpcal4	82	171	**2.09**	BI296503	Hippocalcin-like 4
Hspa2	194	129	**0.67**	BF410146	heat shock 70kDa protein 2
Hspa4	62	153	**2.46**	AF077354	heat shock protein 4
Hspca	295	2016	**6.82**	BG671521	heat shock protein 1, alpha
Hspcb	249	904	**3.63**	BI285700	heat shock 90kDa protein 1, beta
Ifi27l	1002	585	**0.58**	NM_130743	interferon, alpha-inducible protein 27-like
Kalrn	60	194	**3.22**	NM_032062	kalirin, RhoGEF kinase
Khdrbs1	70	131	**1.87**	AF393783	KH domain containing, RNA binding, signal transductio
LOC289606	164	328	**2.00**	BF404116	similar to Gamma-aminobutyric-acid receptor alpha-2 s
LOC365949 /// l	75	247	**3.29**	AI111738	similar to nemo like kinase /// similar to nemo like kinas
LOC497978	103	234	**2.26**	AI101639	similar to diacylglycerol kinase epsilon
LOC498351	295	190	**0.64**	BG670238	similar to signal recognition particle,72 kDa subunit
LOC678826 /// l	548	883	**1.61**	BI295776	similar to sorbin and SH3 domain containing 1 isoform
LOC679252	16	136	**8.73**	AI070638	similar to Myeloid/lymphoid or mixed-lineage leukemia
LOC680419	218	132	**0.61**	AA963364	Similar to Ras suppressor protein 1
LOC682488 /// l	87	178	**2.04**	AI711306	similar to Ras-related protein Rab-1B
LOC683917 /// l	95	353	**3.70**	AI102520	similar to gamma-aminobutyric acid (GABA(A)) recepto
Lsg1	116	30	**0.26**	AA899663	large subunit GTPase 1 homolog (S. cerevisiae)
Pygm	33	90	**2.70**	AI717476	muscle glycogen phosphorylase
Map4k4_predict	162	91	**0.56**	BE117850	mitogen-activated protein kinase kinase kinase kinase
Mapk1	101	221	**2.18**	NM_053842	mitogen activated protein kinase 1
Mapk10	168	263	**1.56**	NM_012806	mitogen activated protein kinase 10
Mapk3	207	355	**1.71**	AF155236	mitogen activated protein kinase 3
Mapk8ip	144	282	**1.96**	AF092450	mitogen activated protein kinase 8 interacting protein
Mapk8ip3	82	224	**2.72**	BF553848	mitogen-activated protein kinase 8 interacting protein 3
Mapk9	40	136	**3.38**	NM_017322	mitogen-activated protein kinase 9
MGC125015	55	125	**2.28**	AA996838	similar to PAK/PLC-interacting protein 1
Nek7_predicted	69	182	**2.65**	BE117215	NIMA (never in mitosis gene a)-related expressed kinas
Nkiras1_predict	284	622	**2.19**	AA997472	NFKB inhibitor interacting Ras-like protein 1 (predicted)
Notch1	55	85	**1.55**	BF389398	Notch gene homolog 1 (Drosophila)
Ntrk2	1283	766	**0.60**	BE102996	Neurotrophic tyrosine kinase, receptor, type 2
Nucks	66	273	**4.11**	NM_022799	nuclear ubiquitous casein kinase and cyclin-dependent
Pacsin1	31	430	**13.84**	NM_017294	protein kinase C and casein kinase substrate in neuron
Pak3	220	421	**1.91**	NM_019210	p21 (CDKN1A)-activated kinase 3
Pde10a	260	587	**2.26**	NM_022236	phosphodiesterase 10A
Pde4b	129	296	**2.29**	AF202733	phosphodiesterase 4B, cAMP specific
Pik3c2a_predict	198	321	**1.62**	AI059449	phosphatidylinositol 3-kinase, C2 domain containing, al
Pik3r2	122	235	**1.93**	NM_022185	phosphatidylinositol 3-kinase, regulatory subunit, polyp
Pip5k1a_predic	41	107	**2.65**	BG153281	phosphatidylinositol-4-phosphate 5-kinase, type 1, alph
Pip5k2a	40	87	**2.14**	NM_053926	phosphatidylinositol-4-phosphate 5-kinase, type II, alph
Pkia	123	199	**1.62**	AA996685	protein kinase inhibitor, alpha
Plaa	110	213	**1.95**	NM_053866	phospholipase A2, activating protein
Plekhb2_predic	*10*	*113*	***11.39***	*AW254369*	*pleckstrin homology domain containing, family B (evect*
**Plekhh1_predi**	**78**	**25**	**0.32**	**BI275435**	**pleckstrin homology domain containing, family H (v**
Plekhj1	137	83	**0.60**	AA848429	pleckstrin homology domain containing, family J memb
Ppp1ca	906	1416	**1.56**	NM_031527	protein phosphatase 1, catalytic subunit, alpha isoform
Ppp1r12a	19	168	**8.94**	BF406594	protein phosphatase 1, regulatory (inhibitor) subunit 12
Ppp1r3c	69	159	**2.30**	AW530361	protein phosphatase 1, regulatory (inhibitor) subunit 3C
Ppp1r7	139	248	**1.78**	H32309	protein phosphatase 1, regulatory (inhibitor) subunit 7
Ppp2r1a	57	226	**3.97**	BF394544	protein phosphatase 2 (formerly 2A), regulatory subunit
Ppp2r5e_predic	105	211	**2.01**	BG664036	protein phosphatase 2, regulatory subunit B (B56), eps
Ppp3ca	*20*	*76*	***3.90***	*BF388224*	*Protein phosphatase 3, catalytic subunit, alpha isoform*
Ppp3r1	87	379	**4.37**	NM_017309	protein phosphatase 3, regulatory subunit B, alpha isof

Ppp5c	114	185	**1.62**	NM_031729	protein phosphatase 5, catalytic subunit
Prkaa2	22	187	**8.41**	NM_023991	protein kinase, AMP-activated, alpha 2 catalytic subuni
Prkcbp1	230	602	**2.62**	BM391419	protein kinase C binding protein 1
Prkce	618	1719	**2.78**	AA799421	protein kinase C, epsilon
Prkce	*89*	*34*	***0.38***	*BI301465*	*Protein kinase C, epsilon*
Prkwnk1	52	81	**1.55**	NM_053794	protein kinase, lysine deficient 1
**Ptgds**	**2735**	**4345**	**1.59**	**J04488**	**prostaglandin D2 synthase**
Ptp4a2	327	985	**3.02**	NM_053475	protein tyrosine phosphatase 4a2
Ptpn11	81	45	**0.55**	AI535048	Protein tyrosine phosphatase, non-receptor type 11
Ptpns1	34	99	**2.96**	D38468	protein tyrosine phosphatase, non-receptor type substr
Ptprd	48	289	**6.08**	L19933	protein tyrosine phosphatase, receptor type, D
Ptprf	51	263	**5.16**	X83505	protein tyrosine phosphatase, receptor type, F
Ptprj	*139*	*231*	***1.67***	*NM_017269*	*protein tyrosine phosphatase, receptor type, J*
Rab10	136	219	**1.62**	NM_017359	RAB10, member RAS oncogene family
Rab11b	139	298	**2.15**	D01046	RAB11B, member RAS oncogene family
Rab14	96	706	**7.37**	NM_053589	RAB14, member RAS oncogene family
Rab15	41	96	**2.31**	BF554320	RAB15, member RAS onocogene family
Rab5a	80	189	**2.36**	NM_022692	RAB5A, member RAS oncogene family
Rab5b_predicte	71	129	**1.82**	AA996576	RAB5B, member RAS oncogene family (predicted)
Rab6a	112	271	**2.43**	AA686757	RAB6A, member RAS oncogene family
Rab7	175	314	**1.79**	NM_023950	RAB7, member RAS oncogene family
Ralgps2	21	94	**4.51**	BF525299	Ral GEF with PH domain and SH3 binding motif 2
Ramp2	252	166	**0.66**	NM_031646	receptor (calcitonin) activity modifying protein 2
Rapgef6_predic	29	146	**5.04**	BI289486	Rap guanine nucleotide exchange factor (GEF) 6 (pred
Rasa1	126	217	**1.73**	NM_013135	RAS p21 protein activator 1
Rasgrf2	69	183	**2.64**	NM_053721	RAS protein-specific guanine nucleotide-releasing facto
Rasgrp1	82	241	**2.95**	AF081196	RAS guanyl releasing protein 1
Rasl10b_predic	58	102	**1.76**	AW919370	RAS-like, family 10, member B (predicted)
Rfng	61	116	**1.89**	NM_021849	radical fringe gene homolog (Drosophila)
RGD1309388_r	127	561	**4.43**	BF544981	similar to DIP13 alpha (predicted)
RGD1311455_r	90	230	**2.55**	BE108260	Similar to MAPK-interacting and spindle-stabilizing prot
RGD1560049_r	57	529	**9.24**	AW520764	similar to Dual specificity protein phosphatase 3 (T-DSI
RGD1561090_r	86	659	**7.62**	AI412803	similar to protein tyrosine phosphatase, receptor type, I
RGD1561817_r	85	129	**1.51**	BG666454	similar to Traf2 and NCK interacting kinase, splice varia
RGD1564560_r	27	342	**12.73**	AA850780	similar to RCK (predicted)
Rgs17_predicte	124	476	**3.83**	AI229118	regulator of G-protein signaling 17 (predicted)
Rgs4	68	176	**2.58**	U27767	regulator of G-protein signaling 4
RICS_predictec	*95*	*248*	***2.61***	*BE097238*	*RhoGAP involved in beta-catenin-N-cadherin and NMD*
Rims1	114	224	**1.97**	NM_052829	regulating synaptic membrane exocytosis 1
Riok3_predictec	40	79	**1.98**	BE126478	RIO kinase 3 (yeast) (predicted)
Rnd3	29	98	**3.37**	AI598323	Rho family GTPase 3
Sbf1_predicted	36	156	**4.39**	BF554377	SET binding factor 1 (predicted)
Set_predicted	601	1057	**1.76**	BG381708	SET translocation (predicted)
Sipa1l1	78	180	**2.30**	AF026504	signal-induced proliferation-associated 1 like 1
Snx10	39	105	**2.73**	AI007889	sorting nexin 10
Snx12_predicte	63	229	**3.65**	BF389280	Sorting nexin 12 (predicted)
Snx27	34	489	**14.57**	AA900057	sorting nexin family member 27
Snx4_predicted	67	179	**2.66**	AI029221	Sorting nexin 4 (predicted)
Shoc2	36	167	**4.66**	AI112329	soc-2 (suppressor of clear) homolog (C. elegans)
Sorcs3_predicte	35	95	**2.73**	BF409626	sortilin-related VPS10 domain containing receptor 3 (pr
Synj2	75	138	**1.86**	AY034051	synaptojanin 2
Taok1	34	145	**4.21**	AF084205	TAO kinase 1
Tbl1x_predictec	449	294	**0.65**	BE108225	transducin (beta)-like 1 X-linked (predicted)
Tiam1	22	87	**3.91**	BM389265	T-cell lymphoma invasion and metastasis 1
Tollip_predicted	85	143	**1.68**	BE112982	toll interacting protein (predicted)
Ywhab	214	493	**2.31**	BG380730	tyrosine 3-monooxygenase/tryptophan 5-monooxygena

Transcription

Sample	F3-Cont	F3-Vinc	**Vin/Con**		
Gene Symbol	Raw	Raw	**Ratio**	Genbank	Gene Title
Ahctf1_predicte	49	78	**1.60**	BF396355	AT hook containing transcription factor 1 (predicted)
Ahdc1_predicte	32	109	**3.40**	BG376923	AT hook, DNA binding motif, containing 1 (predicted)
Ank3	116	194	**1.68**	AJ428573	ankyrin 3, epithelial
Ankrd52_predic	52	294	**5.60**	AA964250	ankyrin repeat domain 52 (predicted)
Arid2_predicted	46	110	**2.37**	BI289741	AT rich interactive domain 2 (Arid-rfx like) (predicted)
Arid4a_predicte	24	138	**5.70**	BF391127	AT rich interactive domain 4A (Rbp1 like) (predicted)
Arid4b	66	135	**2.07**	NM_053421	AT rich interactive domain 4B (Rbp1 like)
Basp1	749	1185	**1.58**	NM_022300	brain abundant, membrane attached signal protein 1
Bcl11b_predicte	14	120	**8.85**	BF398531	B-cell leukemia/lymphoma 11B (predicted)

Bcr_predicted	91	627	**6.87**	BE120831	breakpoint cluster region (predicted)
Bhlhb2	498	299	**0.60**	AI548256	Basic helix-loop-helix domain containing, class B2
Blcap	53	101	**1.89**	NM_133582	bladder cancer associated protein homolog (human)
Cbfa2t1_predict	55	107	**1.96**	BE105678	CBFA2T1 identified gene homolog (human) (predicted)
Cdc42se1	252	163	**0.65**	BI284508	CDC42 small effector 1
Ddx17	67	141	**2.11**	BI296754	DEAD (Asp-Glu-Ala-Asp) box polypeptide 17
Ddx24	70	652	**9.32**	AW252511	DEAD (Asp-Glu-Ala-Asp) box polypeptide 24
Ddx3x	32	117	**3.64**	BF391513	DEAD/H (Asp-Glu-Ala-Asp/His) box polypeptide 3, X-lir
Dhx36_predicte	27	832	**31.27**	BE114021	DEAH (Asp-Glu-Ala-His) box polypeptide 36 (predicted
Dmtf1	807	529	**0.66**	BE098802	cyclin D binding myb-like transcription factor 1
**Dnaja4**	**17**	**79**	**4.64**	**BF565278**	**DnaJ (Hsp40) homolog, subfamily A, member 4**
Dnajb1_predicte	137	226	**1.65**	BM384926	DnaJ (Hsp40) homolog, subfamily B, member 1 (predic
Dnajb6	10	75	**7.88**	AW528827	DnaJ (Hsp40) homolog, subfamily B, member 6
Dnajc7	126	367	**2.92**	BI285682	DnaJ (Hsp40) homolog, subfamily C, member 7
Dnd1	36	89	**2.48**	BF552813	dead end homolog 1 (zebrafish)
Etv1_predicted	72	131	**1.83**	AI112936	Ets variant gene 1 (predicted)
Fbxo9	160	252	**1.58**	BG669135	F-box only protein 9
Fbxw2_predicte	49	79	**1.63**	AW915859	F-box and WD-40 domain protein 2 (predicted)
Foxo1a	13	153	**11.98**	BI295511	forkhead box O1A
Fubp1	121	77	**0.64**	AI233709	Far upstream element (FUSE) binding protein 1
Fyn	191	358	**1.88**	NM_012755	fyn proto-oncogene
Gtf2i	83	146	**1.76**	AI715835	general transcription factor II I
Hip1	282	159	**0.56**	BE110671	huntingtin interacting protein 1
Impad1	119	287	**2.41**	BF396528	inositol monophosphatase domain containing 1
Ing3	58	169	**2.92**	BI296751	inhibitor of growth family, member 3
Jun	179	331	**1.84**	BI288619	Jun oncogene
Kit	33	76	**2.29**	NM_022264	v-kit Hardy-Zuckerman 4 feline sarcoma viral oncogene
Klf6	109	186	**1.71**	NM_031642	Kruppel-like factor 6
Klhl24	116	451	**3.90**	BF550390	kelch-like 24 (Drosophila)
LOC303057	31	116	**3.71**	AI411326	similar to step II splicing factor SLU7; DNA segment, Cl
LOC309957	81	437	**5.41**	BI281877	similar to myocyte enhancer factor 2C
LOC362154 /// I	215	749	**3.49**	BF550329	brain zinc finger protein /// similar to brain Zn-finger pro
LOC500591	127	720	**5.66**	BF564825	similar to calmodulin-binding transcription activator 1
LOC678886 /// I	405	687	**1.70**	BE109666	similar to Enhancer of rudimentary homolog
LOC679725 /// I	35	133	**3.81**	BI296626	similar to MASK-4E-BP3 protein
LOC680802 /// I	20	83	**4.11**	BE101875	similar to Zinc finger protein 45 (BRC1744)
LOC685277	131	721	**5.50**	BE104102	Similar to liver-specific bHLH-Zip transcription factor
**LOC685374**	**100**	**151**	**1.52**	**BG671371**	**similar to ankyrin repeat domain 13c**
LOC685707 /// I	31	83	**2.64**	BF387266	neuron navigator 1 (predicted) /// similar to neuron navi
LOC685865	280	178	**0.64**	BG371744	Similar to zinc finger protein 458
LOC686480 /// I	237	91	**0.38**	AW528215	similar to Protein C12orf11 (Sarcoma antigen NY-SAR-
LOC687516	112	180	**1.61**	BI296193	similar to zinc finger protein 146
LOC688133	255	170	**0.67**	BE114154	similar to mbt domain containing 1
LOC688144 /// I	65	137	**2.12**	BE105705	similar to ankyrin repeat domain 40
LOC690038	65	113	**1.74**	AI009167	Similar to Bcl2-associated athanogene 2
Mcf2l	20	80	**4.00**	NM_053951	mcf.2 transforming sequence-like
Mll	74	120	**1.62**	BG671918	myeloid/lymphoid or mixed-lineage leukemia
Mll5	33	227	**6.77**	BE117891	myeloid/lymphoid or mixed-lineage leukemia 5 (trithora
Mpp5_predictec	39	235	**6.08**	AI072027	membrane protein, palmitoylated 5 (MAGUK p55 subfa
Mpp6_predictec	89	193	**2.17**	BE097259	membrane protein, palmitoylated 6 (MAGUK p55 subfa
Mta1	53	103	**1.93**	AJ132046	metastasis associated 1
Mtpn	129	334	**2.59**	AI711244	myotrophin
Mxd4_predictec	34	113	**3.35**	BI274201	Max dimerization protein 4 (predicted)
Mxi1	52	103	**1.96**	NM_013160	Max interacting protein 1
Mycl1	108	186	**1.72**	BI300996	v-myc myelocytomatosis viral oncogene homolog 1, lur
Ncor1	37	240	**6.54**	BE100543	nuclear receptor co-repressor 1
Nfia	36	80	**2.23**	D78019	nuclear factor I/A
Nfib	178	842	**4.73**	BE099050	nuclear factor I/B
Nfix	48	290	**6.09**	BF420722	nuclear factor I/X
Nfyc	38	84	**2.23**	NM_012866	nuclear transcription factor-Y gamma
Nol6_predicted	74	114	**1.53**	AI409184	nucleolar protein family 6 (RNA-associated) (predicted)
Nolc1	114	175	**1.54**	M94288	nucleolar and coiled-body phosphoprotein 1
Nono	121	222	**1.84**	BF555394	non-POU domain containing, octamer-binding
Npat_predicted	101	67	**0.66**	AI014116	nuclear protein in the AT region (predicted)
Npat_predicted	122	64	**0.52**	BF391717	Nuclear protein in the AT region (predicted)
Nr1h2	62	114	**1.84**	AI169222	nuclear receptor subfamily 1, group H, member 2
Nr2f2	10	79	**7.83**	NM_080778	nuclear receptor subfamily 2, group F, member 2
Nr2f6	56	91	**1.63**	NM_139113	nuclear receptor subfamily 2, group F, member 6
Nr4a2	61	118	**1.94**	U72345	nuclear receptor subfamily 4, group A, member 2

Nr4a3	525	1096	**2.09**	NM_031628	nuclear receptor subfamily 4, group A, member 3
**Nrip3_predicte**	**135**	**237**	**1.76**	**BI288541**	**Nuclear receptor interacting protein 3 (predicted)**
NTF2	70	117	**1.67**	BE109988	nuclear transport factor 2
Nucb1	259	397	**1.53**	Z36277	nucleobindin 1
Os-9	132	88	**0.66**	AI169058	amplified in osteosarcoma
Plag1	69	115	**1.66**	BI290063	pleiomorphic adenoma gene 1
Pogz_predicted	34	75	**2.23**	AI177507	pogo transposable element with ZNF domain (predictec
Psip1	72	111	**1.54**	BF397366	PC4 and SFRS1 interacting protein 1
pur-beta	52	92	**1.79**	BI284455	transcription factor Pur-beta
Rala	87	344	**3.96**	NM_031093	v-ral simian leukemia viral oncogene homolog A (ras re
Recc1	137	476	**3.47**	BM391856	replication factor C 1
Rexo4	44	102	**2.31**	AI237143	REX4, RNA exonuclease 4 homolog (S. cerevisiae)
RGD1305133	36	352	**9.69**	BE116128	similar to Ab2-008
RGD1306327	169	457	**2.71**	AW530286	similar to downregulated in renal cell carcinoma
RGD1307526	292	190	**0.65**	BE114469	similar to modulator of estrogen induced transcription
RGD1310712_p	68	127	**1.87**	BI297740	similar to EMSY protein (predicted)
RGD1562348_p	338	811	**2.40**	BE117929	similar to ankyrin repeat domain protein 17 isoform b (p
RGD1562639_p	49	86	**1.74**	BG670537	similar to c-myc promoter binding protein (predicted)
RGD1562686_p	37	163	**4.41**	BI288731	similar to genetic suppressor element 1 (predicted)
RGD1563119_p	*125*	*841*	***6.75***	*BE104219*	*similar to MADS box transcription enhancer factor 2, pc*
RGD1564056_p	2	77	**51.63**	AI500735	similar to cellular repressor of E1A-stimulated genes 2
RGD1565099_p	10	174	**17.42**	AI045857	similar to BTEB3 protein (predicted)
RGD1565496_p	180	401	**2.22**	BF283779	similar to Butyrate-induced transcript 1 (predicted)
RGD1565549_p	88	307	**3.47**	BE113614	similar to polybromo-1 (predicted)
RGD1565591_p	140	262	**1.87**	BG380847	similar to Ski protein (predicted)
RGD1565646_p	32	121	**3.74**	BF548737	similar to SOX2 protein (predicted)
Rtel1	83	53	**0.64**	BI274548	regulator of telomere elongation helicase 1
Sdccag33_pred	131	62	**0.48**	BF411100	serologically defined colon cancer antigen 33 (predictec
Sertad2	59	146	**2.46**	BI295862	SERTA domain containing 2
Sp1	39	163	**4.22**	AI705174	Sp1 transcription factor
Srebf1	37	100	**2.74**	AF286470	sterol regulatory element binding factor 1
Srebf2	763	482	**0.63**	AI170663	sterol regulatory element binding factor 2 (predicted)
Sox4_predicted	66	101	**1.53**	BI297183	SRY-box containing gene 4 (predicted)
Tcf20	108	168	**1.55**	BF394639	transcription factor 20
Tcf4	269	1307	**4.86**	NM_053369	transcription factor 4
Thoc2_predicte	26	139	**5.26**	AI556851	THO complex 2 (predicted)
Tnks2_predicte	51	121	**2.39**	H32233	tankyrase, TRF1-interacting ankyrin-related ADP-ribose
Tox_predicted	64	107	**1.69**	AI101139	thymocyte selection-associated HMG box gene (predic
Tpr	9	98	**11.44**	BE118639	translocated promoter region
Tspyl4	629	1359	**2.16**	BI281738	TSPY-like 4
Ubn1_predicted	10	108	**10.94**	AI578566	ubinuclein 1 (predicted)
Ubtf	51	90	**1.77**	AI407688	upstream binding transcription factor, RNA polymerase
Usf1	59	129	**2.20**	NM_031777	upstream transcription factor 1
Vezf1_predictec	41	77	**1.86**	BI284190	vascular endothelial zinc finger 1 (predicted)
Zbtb20_predicte	155	543	**3.50**	BF415114	zinc finger and BTB domain containing 20 (predicted)
Zbtb4_predictec	1082	681	**0.63**	BM384146	zinc finger and BTB domain containing 4 (predicted)
Zcchc7_predicte	74	285	**3.84**	AI535316	Zinc finger, CCHC domain containing 7 (predicted)
Zfhx1b	28	176	**6.28**	BG377397	zinc finger homeobox 1b
Zfhx2	10	83	**7.96**	BI284196	zinc finger homeobox 2
Zfp179	553	368	**0.67**	AF054586	zinc finger protein 179
Zfp180	542	880	**1.62**	U41164	zinc finger protein 180
Zfp238	29	112	**3.89**	NM_022678	zinc finger protein 238
Zfp265	111	182	**1.64**	AF013965	zinc finger protein 265
Zfp365	70	157	**2.23**	BF408799	zinc finger protein 365
Zfp91	60	710	**11.78**	BE111631	zinc finger protein 91
Znf142_predicte	46	93	**2.00**	BE117672	Zinc finger protein 142 (clone pHZ-49) (predicted)
Znf292	11	218	**19.22**	BF388585	zinc finger protein 292
---	104	427	**4.13**	BE099838	Non-coding RNA expressed in the brain, repeat sequer
---	1036	3621	**3.49**	AA963228	Non-coding RNA expressed in the brain, repeat sequer

Translation & Protein Modification

Sample	F3-Cont	F3-Vinc	**Vin/Con**		
Gene Symbol	Raw	Raw	**Ratio**	Genbank	Gene Title
Adarb1	32	117	**3.67**	NM_012894	adenosine deaminase, RNA-specific, B1
Arf1	478	955	**2.00**	NM_022518	ADP-ribosylation factor 1
Arf3	129	298	**2.32**	NM_080904	ADP-ribosylation factor 3
Arl1	350	554	**1.58**	NM_022385	ADP-ribosylation factor-like 1
Arl2bp	81	223	**2.74**	BF567478	ADP-ribosylation factor-like 2 binding protein
Arl6ip5	322	584	**1.82**	NM_023972	ADP-ribosylation factor-like 6 interacting protein 5

Brunol4_predict	*107*	*55*	***0.52***	*AW524497*	*Bruno-like 4, RNA binding protein (Drosophila) (predicte*
Cct6a	33	76	**2.34**	AA875047	Chaperonin subunit 6a (zeta)
Cugbp1	271	474	**1.75**	BF408840	CUG triplet repeat, RNA binding protein 1
Cugbp2	94	209	**2.23**	AF090695	CUG triplet repeat, RNA binding protein 2
Dazap2	263	425	**1.62**	AI102612	DAZ associated protein 2
Eif2s3x	32	97	**3.07**	BG662620	eukaryotic translation initiation factor 2, subunit 3, struc
Eif4a1	340	597	**1.76**	BI284436	eukaryotic translation initiation factor 4A1
Eif4ebp2	189	311	**1.64**	BF419161	eukaryotic translation initiation factor 4E binding protein
Eif4g2 /// Eif4g2	230	406	**1.77**	BG672378	eukaryotic translation initiation factor 4 gamma, 2 /// eu
Eif5	116	928	**8.03**	BE107346	eukaryotic translation initiation factor 5
Eif5a	590	1119	**1.90**	BI283681	eukaryotic translation initiation factor 5A
Eif5b /// LOC68	77	147	**1.90**	BE117773	eukaryotic translation initiation factor 5B /// similar to Eu
Eprs	49	282	**5.79**	BF553211	glutamyl-prolyl-tRNA synthetase
Etf1	183	293	**1.60**	BF285301	eukaryotic translation termination factor 1
Hnrpa2b1_pred	64	213	**3.33**	BG666358	heterogeneous nuclear ribonucleoprotein A2/B1 (predic
Hnrpa3	103	181	**1.76**	AI409455	Heterogeneous nuclear ribonucleoprotein A3
Hnrpu	829	1264	**1.52**	AI177494	heterogeneous nuclear ribonucleoprotein U
Hnrpul1_predict	69	108	**1.56**	BG381219	heterogeneous nuclear ribonucleoprotein U-like 1 (prec
Khsrp	251	750	**2.98**	BI295086	KH-type splicing regulatory protein
Larp5_predicted	266	170	**0.64**	AI228249	La ribonucleoprotein domain family, member 5 (predicte
LOC297481	106	238	**2.24**	AI575608	similar to eukaryotic translation initiation factor 4E mem
LOC502603	38	146	**3.86**	AI145768	similar to splicing factor p54
LOC680726	163	248	**1.52**	AW144676	similar to RNA binding motif, single stranded interacting
LOC686858 /// l	73	110	**1.51**	AW531791	similar to small nuclear RNA activating complex, polype
Pabpn1	358	555	**1.55**	AW525563	poly(A) binding protein, nuclear 1
Papola_predicte	61	299	**4.88**	BE108853	poly (A) polymerase alpha (predicted)
Pum1_predicted	54	140	**2.58**	BI282129	pumilio 1 (Drosophila) (predicted)
Qki	143	240	**1.68**	BE102226	Quaking homolog, KH domain RNA binding (mouse)
RAMP4	79	138	**1.75**	AI103695	ribosome associated membrane protein 4
Rbm14	27	75	**2.76**	BG670091	RNA binding motif protein 14
Rbm25_predicte	54	210	**3.92**	AW434972	RNA binding motif protein 25 (predicted) /// similar to RI
Rbm5	261	777	**2.98**	BI293987	RNA binding motif protein 5
Rbm9_predicted	90	543	**6.04**	BE111095	RNA binding motif protein 9 (predicted)
RGD1560397_p	447	808	**1.81**	BF284305	similar to RNA-binding protein Musashi2-S (predicted)
Rnase4	191	290	**1.52**	NM_020082	ribonuclease, RNase A family 4
Rnpc2	185	390	**2.11**	BG372903	RNA-binding region (RNP1, RRM) containing 2
Rps6ka5_predic	21	154	**7.26**	AI045780	ribosomal protein S6 kinase, polypeptide 5 (predicted)
Serbp1	1544	2586	**1.68**	AF388527	Serpine1 mRNA binding protein 1
Sf3b1	66	1024	**15.56**	BM384277	splicing factor 3b, subunit 1
Sf3b2_predicted	39	99	**2.52**	BE098910	splicing factor 3b, subunit 2 (predicted)
Tsn	90	181	**2.02**	NM_021762	translin

Miscellaneous & Unknown

Sample	F3-Cont	F3-Vinc	**Vin/Con**		
Gene Symbol	Raw	Raw	**Ratio**	Genbank	Gene Title
---	93	200	**2.14**	AW535280	Retroviral-like ovarian specific transcript 30-1 mRNA
---	249	468	**1.88**	BI303933	Transcribed locus, strongly similar to XP_226779.4 zin
Aff4_predicted	5	86	**16.45**	AI556957	AF4/FMR2 family, member 4 (predicted)
Dmxl1_predicte	37	100	**2.68**	AA924048	Dmx-like 1 (predicted)
Fyttd1	16	107	**6.53**	AI501447	forty-two-three domain containing 1
Gramd3	101	260	**2.58**	C06752	GRAM domain containing 3
LOC289378	117	70	**0.60**	BI288621	similar to B0432.8
LOC308398	49	101	**2.06**	BE107128	similar to F28C1.3a
LOC363544	379	241	**0.64**	AI012506	similar to 2610111M03Rik protein
LOC498962	118	74	**0.63**	BM387366	similar to C50H11.1
LOC681715 /// l	48	177	**3.67**	BF416086	similar to CG9047-PA, isoform A
LOC683839 /// l	98	217	**2.22**	AW921279	hypothetical protein LOC683839 /// hypothetical protein
LOC686503 /// l	88	200	**2.27**	AA944330	similar to CDC42 small effector 2 (predicted) /// hypothe
LOC687166 /// l	51	120	**2.37**	BF420090	similar to gene trap ROSA b-geo 22
LOC690789 /// l	81	210	**2.59**	AI104518	similar to product is unknown~seizure-related gene (pre
Lysmd3	78	175	**2.26**	AI071181	LysM, putative peptidoglycan-binding, domain containir
Mfhas1_predict	49	140	**2.88**	AI136864	malignant fibrous histiocytoma amplified sequence 1 (p
MGC94335	45	78	**1.72**	BF404452	similar to hypothetical protein FLJ22555
Nudcd1_predict	72	116	**1.61**	AI408455	NudC domain containing 1 (predicted)
Ociad1	99	59	**0.60**	BI275477	OCIA domain containing 1
Perq1_predicted	16	101	**6.13**	AW526967	PERQ amino acid rich, with GYF domain 1 (predicted)
PNAS-4	154	500	**3.25**	AW913871	CGI-146 protein
RGD1306101_p	37	233	**6.27**	BE095778	similar to 4933407C03Rik protein (predicted)
RGD1306694_p	87	142	**1.64**	BF523573	similar to hypothetical protein (predicted)

RGD1307615_p	25	142	**5.80**	AI407061	similar to hypothetical protein FLJ13045 (predicted)
RGD1307729_p	68	113	**1.65**	BF419602	similar to KIAA0853 protein (predicted)
RGD1307907_p	*158*	*99*	***0.63***	*AI146080*	*similar to hypothetical protein FLJ14681 (predicted)*
RGD1307981_p	78	144	**1.85**	BF408881	similar to cisplatin resistance-associated overexpressed
RGD1308329_p	178	288	**1.62**	BM390702	similar to KIAA0869 protein (predicted)
RGD1308795_p	42	129	**3.05**	AW143311	similar to hypothetical protein FLJ12994 (predicted)
RGD1309054_p	137	248	**1.82**	AI231159	similar to FKSG26 protein (predicted)
RGD1309450_p	58	91	**1.57**	BI291600	similar to KIAA2010 protein (predicted)
RGD1309550	222	340	**1.53**	BI295047	similar to hypothetical protein D12Ertd771e
RGD1309752	15	159	**10.91**	AW524173	Similar to hypothetical protein D630010C10
RGD1309995_p	22	113	**5.20**	BF398054	similar to CG13957-PA (predicted)
RGD1310052_p	34	77	**2.26**	BF561001	similar to hypothetical protein FLJ40362 (predicted)
RGD1310139_p	167	107	**0.64**	AI144739	Similar to KIAA0303 (predicted)
RGD1310433_p	50	105	**2.11**	AW254450	similar to mKIAA1757 protein (predicted)
RGD1310474_p	9	110	**12.14**	BF567629	similar to KIAA0423 (predicted)
RGD1311595	138	569	**4.13**	BE107859	similar to KIAA2026 protein
RGD1311678	187	674	**3.61**	AW524670	Similar to 4921517L17Rik protein
**RGD1311958_p**	**104**	**388**	**3.74**	**BF563206**	**similar to 6430514L14Rik protein (predicted)**
RGD1312005_p	72	132	**1.83**	BF392349	similar to DD1 (predicted)
RGD1559605_p	***17***	***409***	***24.57***	***BE101933***	***Similar to hypothetical protein FLJ25477 isoform 2***
RGD1559693_p	69	147	**2.11**	BF400799	similar to Hypothetical protein 6330514E13 (predicted)
RGD1559930_p	20	151	**7.50**	AW920445	similar to mKIAA0256 protein (predicted)
RGD1560834_p	115	256	**2.23**	BE108367	similar to FRBZ1 protein (FRBZ1) (predicted)
RGD1560924_p	37	161	**4.38**	AI011501	similar to C230080I20Rik protein (predicted)
RGD1561500_p	259	171	**0.66**	BM390695	similar to hypothetical protein FLJ14800 (predicted)
RGD1561597_p	56	174	**3.13**	BE118049	similar to mKIAA0518 protein (predicted)
RGD1561653_p	60	706	**11.79**	AI059295	similar to HECT domain containing 1 (predicted)
RGD1562123_p	100	247	**2.47**	BE117893	similar to hypothetical protein (predicted)
RGD1562407_p	*93*	*193*	***2.07***	*BG665671*	*similar to WAC (predicted)*
RGD1563072_p	126	237	**1.88**	BI290787	similar to hypothetical protein FLJ38984 (predicted)
RGD1563612_p	174	344	**1.98**	BE108162	similar to testhymin (predicted)
RGD1563838_p	34	77	**2.29**	BG665533	similar to leucine zipper protein 2 (predicted)
RGD1563977_p	113	69	**0.61**	AI145746	Similar to protein 4.1G (predicted)
RGD1564625_p	87	139	**1.61**	BF397371	similar to transmembrane protein TM9SF3 (predicted)
RGD1564852_p	73	119	**1.63**	AI575906	similar to hypothetical protein FLJ14503 (predicted)
RGD1564943_p	96	243	**2.52**	BE118876	similar to 4930429A08Rik protein (predicted)
RGD1564964_p	18	266	**15.04**	BE116698	similar to WD repeat domain 11 protein (predicted)
RGD1564983_p	29	77	**2.67**	BE107672	similar to leucine rich repeat containing 10 (predicted)
RGD1565095_p	82	669	**8.14**	AA893212	Similar to hypothetical protein MGC52110 (predicted)
RGD1565267_p	48	147	**3.07**	BM387829	similar to PS1D protein (predicted)
RGD1565556_p	374	1171	**3.13**	AI409823	similar to cajalin 2 isoform a (predicted)
RGD1565602_p	76	182	**2.38**	BM383996	similar to PLU1 (predicted)
RGD1566064_p	69	282	**4.08**	BI289641	similar to KIAA1096 protein (predicted)
RGD1566117_p	135	928	**6.87**	AI555865	similar to hypothetical protein FLJ23033 (predicted)
RGD1566201_p	38	150	**3.96**	BE118739	similar to mKIAA0960 protein (predicted)
Rtn4	68	505	**7.48**	AF051335	reticulon 4
Scoc	223	399	**1.79**	AI029749	short coiled-coil protein
Sel1h	36	86	**2.38**	BI285936	Sel1 (suppressor of lin-12) 1 homolog (C. elegans)
Sgta	113	202	**1.79**	NM_022703	small glutamine-rich tetratricopeptide repeat (TPR)-con
Slitrk1_predicte	41	94	**2.29**	AW523107	SLIT and NTRK-like family, member 1 (predicted)
Slitrk3_predicte	35	105	**2.96**	AA819827	SLIT and NTRK-like family, member 3 (predicted)
Svop	143	255	**1.79**	NM_134404	SV2 related protein
Tmem16c_pred	25	98	**3.98**	AI144648	transmembrane protein 16C (predicted)
**Tmem24**	**537**	**295**	**0.55**	**BG378195**	**transmembrane protein 24**
Trim23	79	126	**1.60**	L04760	tripartite motif protein 23
Ttc3_predicted	242	989	**4.08**	BF388771	tetratricopeptide repeat domain 3 (predicted)
Ttc9c	75	139	**1.86**	BG668930	Tetratricopeptide repeat domain 9C
Ttyh1_predictec	1113	2975	**2.67**	AI412746	tweety homolog 1 (Drosophila) (predicted)
Wdr37_predicte	105	712	**6.77**	BG662814	WD repeat domain 37 (predicted)
Wdr48_predicte	82	134	**1.63**	BE101118	WD repeat domain 48 (predicted)
Wdr59	81	135	**1.66**	AW527783	WD repeat domain 59

	**EST's**				
Sample	F3-Cont	F3-Vinc	**Vin/Con**		
Gene Symbol	Raw	Raw	**Ratio**	Genbank	Gene Title
---	180	667	**3.71**	BF406608	Transcribed locus, strongly similar to XP_223397.3 sin
---	12	84	**7.08**	BE109363	Transcribed locus, strongly similar to XP_579796.1 hyp
Fam31b_predic	24	134	**5.57**	BM385853	Family with sequence similarity 31, member B (predicte
LOC366300	598	1122	**1.88**	AI170706	hypothetical LOC366300

LOC682182 /// l	51	77	**1.52**	BE095833	similar to RIKEN cDNA 1110067D22 (predicted) /// simi
RGD1304711_p	354	222	**0.63**	AW526713	Similar to RIKEN cDNA 4921521J11 (predicted)
RGD1305387	76	120	**1.58**	BI288527	similar to RIKEN cDNA 2610207I16
RGD1305492_p	48	96	**2.01**	BG670905	similar to RIKEN cDNA 1700034P14 (predicted)
RGD1305671_p	29	186	**6.46**	BE116226	Similar to expressed sequence AI317237 (predicted)
RGD1305755	***51***	***285***	***5.62***	***AI179665***	***Similar to RIKEN cDNA 5033406L14***
RGD1305903_p	31	125	**4.05**	AI556398	similar to RIKEN cDNA G630024C07 gene (predicted)
RGD1306053	49	91	**1.85**	BF392629	similar to RIKEN cDNA 1200016B17
RGD1306067	61	243	**4.00**	AW141642	similar to chromosome 20 open reading frame 6
RGD1306284	273	180	**0.66**	AA866227	similar to RIKEN cDNA 1110005A03
RGD1306739_p	135	83	**0.61**	AI171288	similar to RIKEN cDNA 1700040L02 (predicted)
RGD1306873	33	137	**4.16**	AI717725	similar to RIKEN cDNA 2210010N04 gene
RGD1308290_p	53	96	**1.81**	AA894335	similar to RIKEN cDNA 5730454B08 (predicted)
RGD1309104_p	116	395	**3.40**	BF388757	similar to RIKEN cDNA 1700025G04 gene (predicted)
RGD1309385_p	39	238	**6.14**	BG372587	similar to RIKEN cDNA E030034P13 (predicted)
RGD1310127	139	88	**0.64**	AA894060	similar to cDNA sequence BC017158
RGD1310351_p	47	238	**5.04**	BI296537	similar to RIKEN cDNA 4732418C07 (predicted)
RGD1310686	223	347	**1.56**	BI294949	similar to chromosome 16 open reading frame 5
RGD1310722_p	42	690	**16.34**	AW534218	similar to RIKEN cDNA D130059P03 gene (predicted)
RGD1311086	221	146	**0.66**	BI288424	similar to RIKEN cDNA 2610029K21
RGD1311456_p	55	266	**4.83**	AI045965	similar to RIKEN cDNA B230380D07 (predicted)
RGD1311783_p	265	469	**1.77**	BG668881	similar to RIKEN cDNA 2010012O05 (predicted)
RGD1311835	*81*	*136*	***1.69***	*BI284801*	*similar to RIKEN cDNA 1110021N07*
**RGD1359529**	**67**	**166**	**2.48**	**BE096504**	**similar to chromosome 1 open reading frame 63**
RGD1359691	137	48	**0.35**	BF398271	hypothetical LOC287534
RGD1559896_p	31	85	**2.77**	AW253651	similar to RIKEN cDNA 2310022B05 (predicted)
RGD1560479_p	23	211	**9.24**	BF394331	similar to RIKEN cDNA 5330439J01 (predicted)
RGD1560913_p	*5*	*164*	***30.87***	*AA799328*	*similar to expressed sequence AW413625 (predicted)*
RGD1560957_p	137	85	**0.62**	BE119096	similar to RIKEN cDNA 5730466H23 (predicted)
RGD1562563_p	150	283	**1.89**	BE116089	Similar to RIKEN cDNA G430041M01 (predicted)
RGD1562618_p	74	136	**1.85**	BG381652	similar to RIKEN cDNA 6030419C18 gene (predicted)
RGD1563001_p	70	343	**4.90**	BI304125	similar to RIKEN cDNA 2010106G01 (predicted)
RGD1563441_p	28	77	**2.74**	BF406306	similar to RIKEN cDNA A030009H04 (predicted)
RGD1563912_p	441	868	**1.97**	BI282028	RGD1563912 (predicted)
RGD1564227_p	39	158	**4.06**	BI296242	similar to RIKEN cDNA 5730557B15 (predicted)
RGD1564379_p	66	100	**1.52**	BF284939	RGD1564379 (predicted)
RGD1564778_p	131	259	**1.97**	AI168933	similar to RIKEN cDNA 4121402D02 (predicted)
RGD1564957_p	36	138	**3.89**	BF387898	similar to RIKEN cDNA 3110007P09 (predicted)
RGD1565957_p	514	325	**0.63**	BI294688	similar to RIKEN cDNA 1110061N23 (predicted)
---	200	126	**0.63**	AF023090	Transcribed locus
---	131	885	**6.75**	BF398122	Transcribed locus
---	31	76	**2.48**	AI029767	Transcribed locus
---	66	125	**1.91**	AA956307	Transcribed locus
---	25	84	**3.35**	BF398673	Transcribed locus
---	9	115	**13.55**	BE117514	Transcribed locus
---	189	120	**0.63**	AI146156	Transcribed locus
---	954	583	**0.61**	BM389272	Transcribed locus
---	252	469	**1.86**	BF420311	Transcribed locus
---	327	892	**2.72**	BM384203	Transcribed locus
---	43	232	**5.43**	BF394499	Transcribed locus
---	17	90	**5.26**	BF403330	Transcribed locus
---	87	51	**0.59**	BE106592	Transcribed locus
---	88	177	**2.01**	H34328	Transcribed locus
---	45	220	**4.93**	BG668689	Transcribed locus
---	44	167	**3.79**	BF394561	Transcribed locus
---	81	36	**0.44**	BI300158	Transcribed locus
---	205	1136	**5.56**	BF546710	Transcribed locus
---	70	183	**2.60**	BF522208	Transcribed locus
---	32	109	**3.38**	AA848370	Transcribed locus
---	217	135	**0.62**	BF390904	Transcribed locus
---	122	77	**0.64**	AW524239	Transcribed locus
---	79	156	**1.98**	BE119960	Transcribed locus
---	106	64	**0.60**	BF415784	Transcribed locus
---	54	247	**4.59**	BF404490	Transcribed locus
---	32	187	**5.85**	BF404446	Transcribed locus
---	335	200	**0.60**	BF391580	Transcribed locus
---	41	90	**2.21**	BF405168	Transcribed locus
---	83	258	**3.11**	BF393011	Transcribed locus
---	206	93	**0.45**	BE120509	Transcribed locus

---	313	204	**0.65**	AW521804	Transcribed locus
---	73	113	**1.54**	BF411608	Transcribed locus
---	145	289	**1.99**	AW526127	Transcribed locus
---	48	247	**5.16**	AW529817	Transcribed locus
---	26	76	**2.93**	BM958510	Transcribed locus
---	27	81	**2.97**	BF396580	Transcribed locus
**---**	**11**	**78**	**6.85**	**BE105421**	**Transcribed locus**
---	45	83	**1.83**	AI113308	Transcribed locus
---	263	476	**1.81**	BE098012	Transcribed locus
---	600	314	**0.52**	H34760	Transcribed locus
---	*43*	*252*	***5.82***	*BF545930*	*Transcribed locus*
---	23	153	**6.80**	BF548061	Transcribed locus
---	1301	856	**0.66**	BE099398	Transcribed locus
---	106	42	**0.40**	AW920217	Transcribed locus
---	38	81	**2.14**	BF291053	Transcribed locus
---	1181	1892	**1.60**	BF544005	Transcribed locus
---	110	271	**2.47**	AW522341	Transcribed locus
---	360	574	**1.59**	BF390608	Transcribed locus
---	66	141	**2.15**	AI102173	Transcribed locus
---	26	105	**4.00**	AW531516	Transcribed locus
---	32	86	**2.70**	AI044545	Transcribed locus
---	36	94	**2.63**	AI601993	Transcribed locus
---	254	149	**0.59**	BE096857	Transcribed locus
---	388	225	**0.58**	BG380768	Transcribed locus
---	112	297	**2.65**	AW523015	Transcribed locus
---	196	311	**1.59**	AW529108	Transcribed locus
---	955	608	**0.64**	BG668719	Transcribed locus
---	62	108	**1.73**	H31734	Transcribed locus
---	34	691	**20.25**	AI145951	Transcribed locus
---	173	115	**0.66**	AW529067	Transcribed locus
---	71	127	**1.80**	AI575856	Transcribed locus
---	74	196	**2.65**	AI412813	Transcribed locus
---	53	142	**2.68**	BG664080	Transcribed locus
---	127	265	**2.08**	AI575519	Transcribed locus
---	113	300	**2.66**	AA956982	Transcribed locus
---	7	172	**26.30**	AI030231	Transcribed locus
---	328	188	**0.57**	AI071202	Transcribed locus
---	89	35	**0.39**	BF392359	Transcribed locus
---	8	197	**23.43**	BF400601	Transcribed locus
---	33	336	**10.30**	BE120522	Transcribed locus
---	42	81	**1.92**	AA956085	Transcribed locus
---	23	136	**5.82**	BI303858	Transcribed locus
---	192	86	**0.45**	BE106361	Transcribed locus
---	64	331	**5.16**	BF549454	Transcribed locus
---	13	112	**8.85**	BE096515	Transcribed locus
---	12	79	**6.83**	BE111692	Transcribed locus
---	153	361	**2.36**	AI556642	Transcribed locus
---	26	124	**4.76**	BI295733	Transcribed locus
---	84	143	**1.71**	AA850595	Transcribed locus
---	79	161	**2.05**	BF406435	Transcribed locus
---	29	139	**4.79**	AA956757	Transcribed locus
---	82	287	**3.51**	BM391661	Transcribed locus
---	135	343	**2.54**	BI296013	Transcribed locus
---	135	89	**0.66**	AI227988	Transcribed locus
---	75	46	**0.61**	BI280337	Transcribed locus
---	30	93	**3.12**	BE096618	Transcribed locus
---	200	122	**0.61**	BF389889	Transcribed locus
---	54	115	**2.14**	BI291162	Transcribed locus
---	59	118	**2.00**	AI043817	Transcribed locus
---	*173*	*84*	***0.48***	*BF399309*	*Transcribed locus*
---	81	54	**0.66**	BF405616	Transcribed locus
---	20	107	**5.45**	BI294889	Transcribed locus
---	23	80	**3.53**	BE107619	Transcribed locus
---	89	170	**1.91**	BF419818	Transcribed locus
---	34	354	**10.29**	BI300794	Transcribed locus
---	183	113	**0.62**	BF283381	Transcribed locus
---	131	228	**1.74**	AI231225	Transcribed locus
---	*78*	*41*	***0.53***	*BF391128*	*Transcribed locus*
---	34	92	**2.74**	BI295124	Transcribed locus

---	*70*	*253*	***3.62***	*AA899937*	*Transcribed locus*
---	186	287	**1.55**	BF393945	Transcribed locus
---	253	161	**0.64**	BE107167	Transcribed locus
---	62	96	**1.53**	AI232217	Transcribed locus
---	84	55	**0.66**	BF283404	Transcribed locus
---	302	171	**0.57**	AI229933	Transcribed locus
---	273	144	**0.53**	AW915115	Transcribed locus
---	*15*	*134*	***8.86***	*BF406304*	*Transcribed locus*
---	***112***	***322***	***2.88***	***AA944136***	***Transcribed locus***
---	166	105	**0.63**	BF562962	Transcribed locus
---	257	603	**2.35**	BF546770	Transcribed locus
---	348	198	**0.57**	BF284027	Transcribed locus
---	144	314	**2.18**	AI229321	Transcribed locus
---	15	90	**6.07**	H31285	Transcribed locus
---	176	315	**1.79**	AA874903	Transcribed locus
---	59	188	**3.21**	BE100612	Transcribed locus
---	204	129	**0.63**	AI146262	Transcribed locus
---	55	121	**2.19**	BF416395	Transcribed locus
---	127	61	**0.48**	AA817920	Transcribed locus
---	3	122	**40.67**	BG668477	Transcribed locus
---	81	135	**1.66**	AI072068	Transcribed locus
---	55	178	**3.25**	AW532389	Transcribed locus
---	51	253	**4.95**	BF409092	Transcribed locus
---	38	81	**2.14**	AA997253	Transcribed locus
---	21	81	**3.87**	BE096277	Transcribed locus
---	48	93	**1.94**	BE120904	Transcribed locus
---	159	597	**3.75**	AA925373	Transcribed locus
---	36	145	**3.99**	BM390588	Transcribed locus
---	28	106	**3.77**	AI229409	Transcribed locus
---	250	156	**0.62**	BE108597	Transcribed locus
---	92	147	**1.59**	BF559356	Transcribed locus
---	115	1006	**8.77**	BE109132	Transcribed locus
---	170	292	**1.72**	BG667918	Transcribed locus
---	74	133	**1.80**	BG671630	Transcribed locus
---	79	158	**1.99**	BF555825	Transcribed locus
---	140	89	**0.64**	AI072798	Transcribed locus
**---**	**85**	**213**	**2.50**	**AA925807**	**Transcribed locus**
---	20	156	**7.90**	AI071698	Transcribed locus
---	90	47	**0.53**	BG665568	Transcribed locus
---	544	352	**0.65**	AI230360	Transcribed locus
---	***190***	***76***	***0.40***	***AI102821***	***Transcribed locus***
---	*175*	*111*	***0.64***	*AA926109*	*Transcribed locus*
---	556	1131	**2.03**	BM958512	Transcribed locus
---	117	191	**1.63**	AW921244	Transcribed locus
---	38	348	**9.25**	BI281615	Transcribed locus
---	206	865	**4.19**	BG372598	Transcribed locus
---	339	219	**0.65**	BF563716	Transcribed locus
---	868	1987	**2.29**	BF555795	Transcribed locus
---	80	52	**0.65**	AW523077	Transcribed locus
---	90	375	**4.16**	BE108047	Transcribed locus
---	87	141	**1.63**	AI237079	Transcribed locus
---	232	361	**1.55**	AI176342	Transcribed locus
---	*46*	*100*	***2.17***	*AA859319*	*Transcribed locus*
---	74	212	**2.86**	BE120748	Transcribed locus
---	107	603	**5.66**	BE105050	Transcribed locus
---	155	83	**0.54**	AA875457	Transcribed locus
---	55	260	**4.77**	AI717668	Transcribed locus
---	*110*	*59*	***0.54***	*AI070489*	*Transcribed locus*
---	843	1377	**1.63**	BF285731	Transcribed locus
---	162	92	**0.57**	BI280114	Transcribed locus
---	171	91	**0.53**	BE105488	Transcribed locus
---	*70*	*124*	***1.78***	*BE110067*	*Transcribed locus*
---	372	764	**2.05**	AW530378	Transcribed locus
---	47	115	**2.45**	BE121056	Transcribed locus
---	26	189	**7.40**	AI555166	Transcribed locus
---	28	97	**3.51**	AI060117	Transcribed locus
---	*15*	*83*	***5.37***	*AI145015*	*Transcribed locus*
---	120	315	**2.63**	AA819045	Transcribed locus
---	60	170	**2.86**	AW524106	Transcribed locus

---	305	144	**0.47**	BF409213	Transcribed locus
---	16	392	**24.17**	BE108751	Transcribed locus
---	74	322	**4.37**	BE114458	Transcribed locus
---	31	94	**3.02**	BF391155	Transcribed locus
---	12	146	**12.61**	AW534519	Transcribed locus
---	47	96	**2.04**	AW534466	Transcribed locus
---	94	728	**7.72**	AI145433	Transcribed locus
---	18	87	**4.84**	BF416420	Transcribed locus
---	28	94	**3.38**	BF390757	Transcribed locus
---	81	132	**1.63**	AI406475	Transcribed locus
---	8	148	**18.44**	AW530527	Transcribed locus
---	19	138	**7.30**	BM386352	Transcribed locus
---	63	130	**2.08**	AI103408	Transcribed locus
**---**	**68**	**1546**	**22.67**	**AI555855**	**Transcribed locus**
---	551	335	**0.61**	H31701	Transcribed locus
---	97	203	**2.09**	AI229240	Transcribed locus
---	10	198	**19.54**	BF402498	Transcribed locus
---	84	55	**0.65**	AI058315	Transcribed locus
---	81	146	**1.79**	BE102139	Transcribed locus
---	*84*	*53*	***0.63***	*AI137306*	*Transcribed locus*
---	150	243	**1.62**	BM386302	Transcribed locus
---	158	430	**2.72**	AI535567	Transcribed locus
---	96	413	**4.29**	BF420785	Transcribed locus
---	123	193	**1.57**	AI011930	Transcribed locus
---	38	78	**2.06**	AI233902	Transcribed locus
---	34	81	**2.40**	BI288184	Transcribed locus
---	79	163	**2.07**	BM383595	Transcribed locus
---	28	108	**3.91**	BF392753	Transcribed locus
---	79	167	**2.11**	BE108208	Transcribed locus
---	86	140	**1.63**	AI717163	Transcribed locus
---	204	355	**1.74**	AW534671	Transcribed locus
---	37	170	**4.55**	BF397054	Transcribed locus
---	41	120	**2.92**	AA900904	Transcribed locus
---	61	164	**2.71**	AA818967	Transcribed locus
---	45	119	**2.64**	BF407470	Transcribed locus
---	85	134	**1.57**	BE107074	Transcribed locus
---	146	234	**1.60**	AI179450	Transcribed locus
---	7	79	**10.61**	BG379394	Transcribed locus
---	190	330	**1.74**	BF411826	Transcribed locus
---	116	1031	**8.89**	AI511069	Transcribed locus
---	48	434	**9.05**	AW252020	Transcribed locus
---	195	129	**0.66**	BI276118	Transcribed locus
**---**	**263**	**706**	**2.69**	**AI112113**	**Transcribed locus**
---	73	161	**2.21**	AW531387	Transcribed locus
---	55	139	**2.51**	BF399121	Transcribed locus
---	21	110	**5.16**	BF284914	Transcribed locus
---	657	1034	**1.57**	BI292687	Transcribed locus
---	43	86	**2.01**	AA859337	Transcribed locus
---	465	1380	**2.97**	AW535380	Transcribed locus
---	96	157	**1.64**	BE106526	Transcribed locus
---	46	78	**1.68**	BF548480	Transcribed locus
---	84	51	**0.60**	BI291457	Transcribed locus
---	103	62	**0.60**	BF418563	Transcribed locus
---	51	94	**1.86**	BE099568	Transcribed locus
---	204	344	**1.69**	BI295869	Transcribed locus
---	470	1446	**3.08**	AI170377	Transcribed locus
---	140	330	**2.37**	BM386385	Transcribed locus
---	136	241	**1.77**	BF412303	Transcribed locus
---	53	589	**11.20**	AW532489	Transcribed locus
---	87	232	**2.66**	BF283340	Transcribed locus
---	113	333	**2.96**	BF550404	Transcribed locus
---	163	92	**0.57**	BF416276	Transcribed locus
---	40	85	**2.14**	BG378933	Transcribed locus
---	116	281	**2.42**	AA955579	Transcribed locus
---	21	96	**4.67**	BF396151	Transcribed locus
---	622	406	**0.65**	BG378070	Transcribed locus
---	100	66	**0.66**	BI286851	Transcribed locus
---	51	117	**2.29**	BE108174	Transcribed locus
---	25	112	**4.59**	BE108246	Transcribed locus

---	288	153	**0.53**	AW143156	Transcribed locus
---	*66*	*129*	***1.96***	*AI101372*	*Transcribed locus*
---	194	359	**1.86**	BF415701	Transcribed locus
---	315	190	**0.60**	BI282767	Transcribed locus
---	23	200	**8.64**	BF402633	Transcribed locus
---	56	221	**3.98**	BE109509	Transcribed locus
---	23	168	**7.45**	AI385171	Transcribed locus
---	77	371	**4.81**	BF408438	Transcribed locus
---	63	382	**6.06**	BM389190	Transcribed locus
---	24	90	**3.72**	BG372400	Transcribed locus
---	40	98	**2.44**	AI101245	Transcribed locus
---	82	193	**2.34**	BE111820	Transcribed locus
---	264	141	**0.53**	AI407719	Transcribed locus
---	53	198	**3.73**	BF400811	Transcribed locus
---	333	517	**1.55**	BM384026	Transcribed locus
---	333	646	**1.94**	BM388843	Transcribed locus
---	75	251	**3.34**	BF400779	Transcribed locus
---	119	76	**0.64**	AI232806	Transcribed locus
---	30	1207	**39.95**	AI228978	Transcribed locus
---	117	62	**0.53**	BG381647	Transcribed locus
---	202	135	**0.67**	AA859010	Transcribed locus
---	716	473	**0.66**	AI171776	Transcribed locus
---	212	607	**2.87**	BE109208	Transcribed locus
---	250	156	**0.62**	BM386212	Transcribed locus
---	206	129	**0.63**	AI598550	Transcribed locus
---	104	232	**2.23**	AA799420	Transcribed locus
---	111	70	**0.63**	BG373057	Transcribed locus
---	***147***	***364***	***2.48***	***AI103530***	***Transcribed locus***
---	57	342	**5.95**	AI555608	Transcribed locus
---	81	123	**1.52**	AW914907	Transcribed locus
---	122	193	**1.58**	BF544403	Transcribed locus
---	52	83	**1.60**	BF542239	Transcribed locus
---	375	206	**0.55**	BI303277	Transcribed locus
---	74	137	**1.86**	AW920828	Transcribed locus
---	67	115	**1.71**	AI407047	Transcribed locus, moderately similar to XP_00114552
---	70	171	**2.46**	BE104676	Transcribed locus, strongly similar to NP_001029332.1
---	693	405	**0.58**	AI454332	Transcribed locus, strongly similar to XP_001081628.1
---	281	498	**1.77**	BF390195	CDNA clone IMAGE:7320582
---	51	135	**2.68**	BF562934	CDNA clone IMAGE:7320582
---	77	962	**12.45**	AI008409	CDNA clone IMAGE:7321089
---	237	145	**0.61**	AI237047	CDNA clone IMAGE:7365681
---	126	80	**0.63**	BF398091	CDNA clone IMAGE:7374368
---	37	203	**5.48**	BE106331	CDNA clone IMAGE:7461178
---	295	469	**1.59**	BI294768	---
---	381	161	**0.42**	BM384537	---
---	46	86	**1.85**	BF547596	---
---	55	99	**1.79**	AI547718	---
---	23	147	**6.29**	AI705744	---
---	195	370	**1.90**	H33235	---
---	120	390	**3.24**	AI103026	---
---	294	688	**2.34**	BE116953	---
---	162	258	**1.59**	BI282114	---
---	302	461	**1.53**	BI275155	---
---	74	271	**3.67**	AW527270	---
---	236	151	**0.64**	BM385286	---
---	109	283	**2.60**	BG372713	---
---	207	849	**4.10**	AA997406	---
---	248	493	**1.99**	BF400907	---
---	1710	3508	**2.05**	_Rat_beta-ac	---
---	240	784	**3.27**	_Rat_beta-ac	---
---	543	1920	**3.54**	_Rat_beta-ac	---
---	126	211	**1.68**	BF556405	---
**---**	**49**	**82**	**1.67**	**AI172311**	**---**
---	2376	1570	**0.66**	AI145313	---
---	14	77	**5.36**	AI112375	---
---	94	61	**0.65**	AA858748	---
---	143	70	**0.49**	BF522861	---
---	382	181	**0.47**	BF545849	---
---	133	200	**1.50**	BE120211	---

---	539	347	**0.64**	BG380279	---
---	51	1212	**23.99**	AI103917	---
---	84	52	**0.62**	AI030449	---
---	102	712	**6.97**	AI101194	---
---	51	139	**2.75**	BI289762	---
---	1779	1185	**0.67**	BE115454	---
---	11	127	**11.37**	BF398431	---
---	37	114	**3.04**	BG668744	---
---	66	100	**1.51**	BE097409	---
---	45	85	**1.89**	AW535011	---
---	42	167	**4.02**	AW527250	---
---	131	84	**0.64**	BE116860	---
---	51	153	**2.98**	BE119914	---
---	*45*	*85*	***1.91***	*BI288579*	---
---	170	345	**2.02**	AW921158	---
---	100	152	**1.53**	BF396974	---
---	129	75	**0.58**	AA859524	---
---	177	96	**0.54**	BE117273	---
---	77	129	**1.68**	AI715113	---
---	246	152	**0.62**	BG373537	---
---	14	78	**5.60**	BF415798	---
---	53	158	**2.97**	BI293026	---
---	47	104	**2.23**	BF397998	---
---	50	80	**1.60**	BF400933	---
---	105	58	**0.55**	BI280367	---
---	131	271	**2.06**	BF544968	---
---	19	106	**5.58**	AA851046	---
---	22	283	**12.82**	BF548081	---
---	403	249	**0.62**	BM383406	---
---	89	53	**0.60**	BG380561	---
---	573	1349	**2.36**	BE101133	---
---	131	86	**0.66**	AI574734	---
---	238	146	**0.61**	BE105500	---
---	27	95	**3.48**	BF386160	---
---	29	171	**5.84**	BE115821	---
---	160	106	**0.66**	BF413298	---
---	76	18	**0.24**	BE111542	---
---	35	134	**3.82**	AI501579	---
---	*101*	*59*	***0.58***	*BF410240*	---
---	87	53	**0.60**	AI013683	---
---	178	277	**1.56**	BF397301	---
---	282	180	**0.64**	AI145935	---
---	88	43	**0.49**	BF419655	---
---	54	192	**3.56**	BF419406	---
---	28	76	**2.73**	BF390754	---
**---**	**55**	**97**	**1.76**	**BF403869**	**---**
---	107	950	**8.92**	AI575254	---
---	45	930	**20.86**	AI710284	---
---	420	279	**0.67**	BF420262	---
---	670	433	**0.65**	BF403383	---
---	29	376	**12.95**	AI045904	---
---	240	151	**0.63**	AI104117	---
---	29	97	**3.37**	AW920849	---
---	306	1144	**3.74**	BF567766	---
---	52	116	**2.21**	AA684862	---
---	18	112	**6.11**	AI059349	---
---	18	105	**5.94**	AI705744	---
---	17	81	**4.74**	BF559198	---
---	5	86	**15.98**	AI234943	---

Note - The bolded genes are similar between male and female gene sets.
The italic genes are similar within the same sex between amygdala and hippocampus.
