# Supplementary material for: Transgenerational Epigenetic Programming of the Brain Transcriptome and Anxiety Behavior
Source: PLoS One. 2008 Nov 18;3(11):e3745. doi: 10.1371/journal.pone.0003745 (PMC2581440; doi:10.1371/journal.pone.0003745)
Supplement: Table S6 — Performance in Light: Dark Box with Low Light Condition. (0.06 MB PDF) [file pone.0003745.s008.pdf]

Supplemental Table S6. Performance in Light:Dark Box with Low Light Condition

Sex	Latency to enter dark		Time on light side		Transitions	
	**Young**	**Aged**	**Young**	**Aged**	**Young**	**Aged**
**Females**						
Control	14 ± 5	31 ± 14	125 ± 7	163 ± 20	9 ± 1	10 ± 1
Vinclozolin	8 ± 4	19 ± 4	120 ± 7	146 ± 13	13 ± 1*	10 ± 1
**Males**						
Control	31 ± 9	21 ± 4	85 ± 21	98 ± 15	6 ± 1	7 ± 1
Vinclozolin	38 ± 9	41 ± 10‡	111 ± 22	123 ± 30	6 ± 1	4 ± 1*

***P < 0.05, compared with Control.**
‡P < 0.10, compared with Control.

Treated young, but not old, female rats increased the number of transitions between the light and dark compartments. Young male rats showed no differences in behavior between treated and controls on any of the measures, however, in aged males, vinclozolin generation rats had a significant decrease in the number of transitions between the light and dark compartments. There was also a trend for these aged rats to demonstrate an increased latency to enter the dark side of the box, which may have been related to their lower motor activity.
